# Morphing Natural Product Platensimycin via Heck, Sonogashira, and One-Pot Sonogashira/Cycloaddition Reactions to Produce Antibiotics with In Vivo Activity

**DOI:** 10.3390/antibiotics11040425

**Published:** 2022-03-23

**Authors:** Youchao Deng, Yuling Li, Zhongqing Wen, Claudia H. Ruiz, Xiang Weng, Michael D. Cameron, Yanwen Duan, Yong Huang

**Affiliations:** 1 Xiangya International Academy of Translational Medicine, Central South University, Changsha 410013, China; dengyouchao1206@163.com (Y.D.); lyl1614787460@163.com (Y.L.); wen17375898093@163.com (Z.W.); 202120919@mail.sdu.edu.cn (X.W.); 2 Departments of Molecular Medicine, The Scripps Research Institute, Jupiter, FL 33458, USA; cruiz@scripps.edu (C.H.R.); cameron@scripps.edu (M.D.C.); 3 Hunan Engineering Research Center of Combinatorial Biosynthesis and Natural Product Drug Discovery, Changsha 410011, China; 4 National Engineering Research Center of Combinatorial Biosynthesis for Drug Discovery, Changsha 410011, China

**Keywords:** platensimycin, *Staphylococcus aureus*, antibiotic, FabF, FabB, C–C cross-coupling reactions

## Abstract

Type II fatty acid synthases are promising drug targets against major bacterial pathogens. Platensimycin (PTM) is a potent inhibitor against *β*-ketoacyl-[acyl carrier protein] synthase II (FabF) and *β*-ketoacyl-[acyl carrier protein] synthase I (FabB), while the poor pharmacokinetics has prevented its further development. In this work, thirty-two PTM derivatives were rapidly prepared via Heck, Sonogashira, and one-pot Sonogashira/cycloaddition cascade reactions based on the Gram-scale synthesis of 6-iodo PTM (**4**). About half of the synthesized compounds were approximately equipotent to PTM against the tested *Staphylococcus aureus* strains. Among them, the representative compounds **4**, **A4**, and **B8** exhibited different plasma protein binding affinity or stability in the human hepatic microsome assay and showed improved in vivo efficacy over PTM in a mouse peritonitis model. In addition, **A4** was also effective in an *S. aureus*-infected skin mouse model. Our study not only significantly expands the known PTM derivatives with improved antibacterial activities in vivo, but showcased that C–C cross-coupling reactions are useful tools to functionalize natural product drug leads.

## 1. Introduction

The antimicrobial resistance crisis is becoming an important public health issue worldwide, mainly due to the overuse of conventional antibiotics and the slow discovery of novel classes of antibiotics [1,2]. Based on the recent survey of over 1400 major hospitals in China, 29.4% of the reported 305,778 *S. aureus* strains are resistant to methicillin [3]. Even in non-hospital environments, methicillin-resistant *Staphylococcus aureus* (MRSA) strains were also identified from communities due to cross-circulation, including public schools and busy subways of a metropolitan city [4]. The discovery of new generations of antibiotics with novel modes of action is thus urgently needed. However, antibiotic discovery remains very challenging despite great strides in the implementation of new antibiotic discovery platforms in the last several years [5]. In addition, many promising antimicrobial hits were initially discovered, but their developments were slowed or even abandoned due to unfavorable pharmacokinetic properties or safety issues.

**Figure 1:**
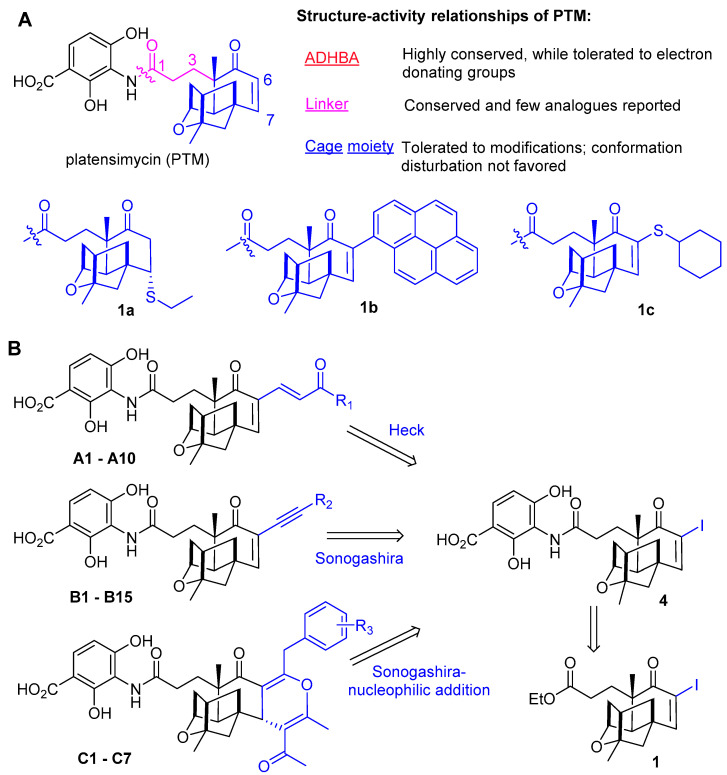
The strategy to modify PTM terpene cage. (**A**) The structure–activity relationships of PTM. (**B**) The retrosynthetic analysis for preparation of 6-acrylyl or alkynyl PTM (**A1**–**A10**, **B1**–**15**) through Heck and Sonogashira reactions and cyclized analogues through a Sonogashira/cycloaddition cascade reaction (**C1**–**C7**).

Platensimycin (PTM) is a recently discovered antibacterial drug leads against many Gram-positive pathogens, such as MRSA and vancomycin-resistant *Enterococci* (VRE) (Figure 1) [6]. It inhibits the biosynthesis of essential fatty acids by strongly binding to the bacterial condensation enzymes *β*-ketoacyl-[acyl carrier protein] synthase II (FabF) and *β*-ketoacyl-[acyl carrier protein] synthase I (FabB) in type II fatty acid synthases (FASII). However, the undesirable pharmacokinetics of PTM is a major issue that prevents it from entering clinics [7]. Previous structure–activity relationship studies of PTM revealed the determinants for its potent antibacterial activity, suggesting that modification of the PTM ketolide is a rapid approach to diversify the PTM structure while retaining its antibiotic activity [8,9,10,11]. However, to the best of our knowledge, most of these PTM analogues were only tested in vitro, and the in vivo fate remains largely unknown in comparison to PTM.

We have recently embarked on a journey of semi-synthesis and biological evaluation of PTM derivatives based on the established pilot-scale production of PTM from microbial fermentation [12]. Several PTM derivatives, such as PTM-2t (**1a**) [13,14], 6-pyrenyl PTM (**1b**) [15], and 6-cyclohexylthio PTM (**1c**) [16], were discovered to show improved in vivo efficacy compared to PTM in a lethal peritonitis mouse model induced by MRSA infection. However, there were still residual MRSA in the mouse kidney after systematic treatments despite these improvements, suggesting the requirement of further improvement of the PTM scaffold. The discovery of alternative PTM derivatives may realize the potential of PTM by targeting FASII against infectious pathogens.

Heck and Sonogashira reactions are widely used to diversify molecular scaffolds, due to their mild reaction conditions and high yields, as well as the availability of a wide range of substrates [17]. One-pot synthesis also shows tremendous advantages in efficiency and environmental sustainability in organic synthesis, and has been instrumental in preparing many bioactive natural or non-natural molecules [18,19,20]. In this work, a new synthetic route was developed to prepare thirty-two PTM derivatives through Heck and Sonogashira reactions, as well as one-pot Sonogashira/cycloaddition cascade reactions (Figure 1B). Notably, seven highly substituted 4*H*-pyrans were constructed through the one-pot reaction in a highly stereochemically controlled fashion. The minimum inhibitory concentrations (MICs) of these new compounds were next determined and about half of them were as potent as PTM against the tested MRSA strains. The selected compounds, including **4**, **A4**, and **B8**, were further evaluated using plasma protein binding and human hepatic microsome assays, as well as molecular docking. Compounds **4**, **A4**, and **B8** showed improved in vivo efficacy in a lethal mouse peritonitis model over PTM, and **A4** was also effective in an *S. aureus*-infected mouse skin model. Our study significantly increases the reservoir of potent FabF/FabB inhibitors, which may become potential antibiotics against Gram-positive pathogens in the future.

## 2. Results

### 2.1. Semisynthesis of PTM Derivatives

**Scheme 1:**
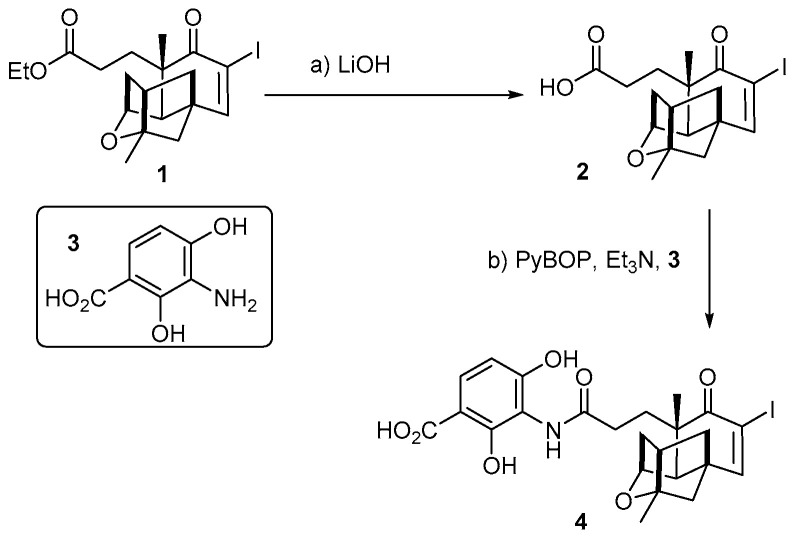
The synthesis of 6-iodo-PTM (**4**). (**a**) LiOH (2 M, 3.0 eq. in MeOH and water, rt; >95%; (**b**) **3**, PyBOP 1.0 eq., Et_3_N 3.0 eq. in the mixture solvent of DMF and DCM, stirred under rt, >80%.

We have recently prepared dozens of PTM derivatives using Suzuki cross-coupling reactions using 6-iodo platensic ethylester (**1**) as the substrate (Scheme 1) [15]. However, there were three more steps involved to obtain the final products after the cross-coupling reactions, which makes rapid functionalization of PTM challenging. Therefore, we envision that 6-iodo PTM (**4**) may be a more suitable intermediate than **1** for derivatization of PTM using C–C cross-coupling reactions since no further deprotection or amide-forming reactions were needed.

In order to synthesize the core intermediate **4**, compound **1** was conveniently obtained using our previous Gram-scale synthetic protocol (Scheme 1) [15]. Next, **1** was selectively hydrolyzed with 2 M LiOH in MeOH/H_2_O, which generated 6-iodo platensic acid (**2**) in a quantitative yield (Figures S1 and S39). The use of MeOH/H_2_O co-solvent is important for the hydrolysis reaction since inefficient hydrolysis of **1** was observed when tetrahydrofuran/H_2_O was used. Further, 3-amino-2,4-dihydroxybenzoic acid (ADHBA) (**3**) was prepared as previously reported [11,21]. An efficient protection-group free strategy for the amide formation between **2** and **3** was next developed using PyBOP as the catalyst, which resulted in the preparation of **4** in a gram-scale synthesis (Figures S2 and S40). Note that we could not obtain **4** using many other common amide coupling catalysts, such as HATU or HBTU [9,10,11].

Next, PTM derivatives **A1**–**A10** were synthesized through classical Heck reaction conditions using Pd(OAc)_2_ as the catalyst, as well as **4** and acrylic esters or acrylic amides as substrates (Scheme 2). The yields for the formation of less polar **A1**–**A5** were relatively higher than those of **A6**–**A10**, suggesting their different reactivity in the cross-coupling reactions (Figures S3–S12 and S41). PTM derivatives **B1**–**B15** were prepared through Sonogashira-type sp^2^–sp cross-couplings in moderate to excellent yields of 65–90% (Figures S13–S27 and S42). No obvious differences in reaction activity were witnessed among alkyl and aryl substituted alkynes. Further, 4*H*-pyrans have been found in several compounds with antibacterial activities [22]. Hu et al. used a Michael-nucleophilic domino reaction to construct functionalized 4H-pyrans using α-(alkyne)-cyclohexanone and acetylacetone [23]. Concerning the high efficiency of a Sonogashira reaction in preparing analogues **B1**–**B15**, a one-pot synthetic strategy was then devised to generate **C1**–**C7** with yields of >70% in the presence of PdCl_2_(PPh_3_)_2_ and AgOAc (Figures S28–S38 and S43).

**Scheme 3:**
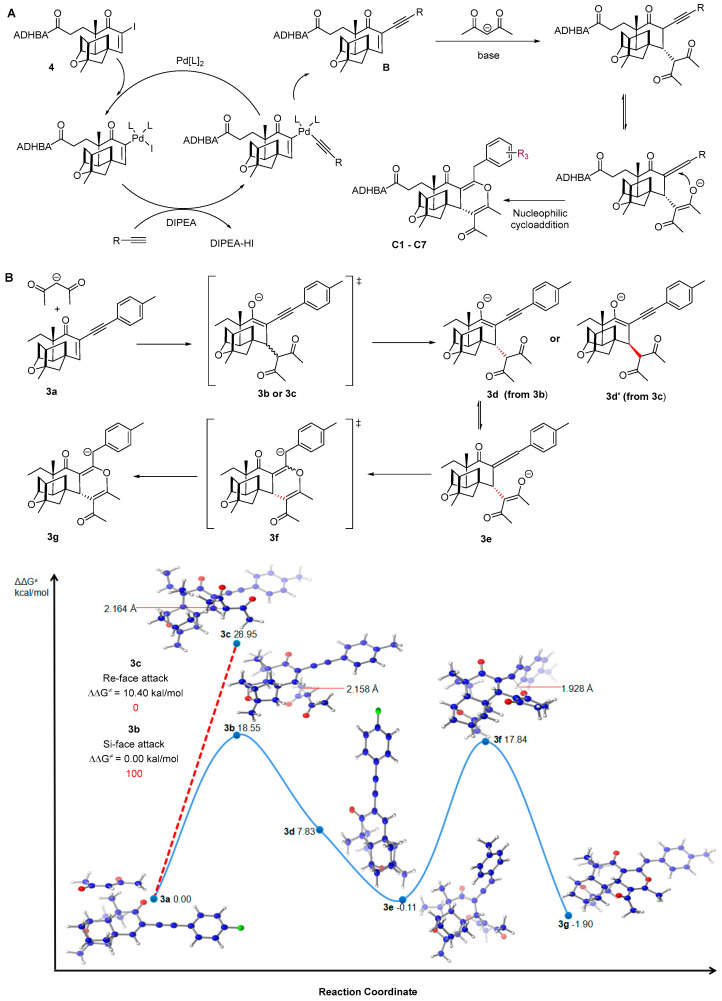
The mechanistic study for the one-pot synthesis of **C1**–**C7**. (**A**) The proposed mechanism for the formation of **C1**–**C7**. (**B**) Density functional theory-based calculation for the asymmetric Michael reaction of the deprotonated acetylacetone to PTM surrogate **3a**, followed by cyclization en route to the formation of **3g**. M06-2X/6-31 + G(d,p)/CPCM (DMSO). All energies in kcal mol*^−^*^1^ and bond distances in Å.

PTM derivatives **C1**–**C7** were obtained only in single isomers, indicating the high stereochemical control of the cascade reactions (Table S1 and Figures S28–S38). A putative reaction mechanism was firstly proposed, involving generating intermediate **B** from **4** through a classical Sonogashira reaction pathway (Scheme 3A). Then, acetylacetone was stereoselectively added to the enone of **B** via Michael reaction, catalyzed by CsCO_3_. Using density functional theory-based computation with the simplified surrogate **3a**, acetylacetone would prefer *Si*-face attack against **B** due to the significant steric effects of PTM ketolide cage moiety (Scheme 3B) [13,24]. The Gibbs free energy of the putative transition state intermediate 3b is 18.55 kcal/mol, while that of **3c** from *Re*-face attack is 28.95 kcal/mol. Next, a more stable allenyl ketone intermediate **3e** may be formed from **3d**. Finally, the product **3g** could be generated through intramolecular cyclic nucleophilic addition. Although the energy barrier of the intramolecular cyclic nucleophilic addition was 17.95 kcal/mol, the reaction proceeded at 90 °C and would be sufficient to overcome this reaction barrier. To the best of our knowledge, it is the first report that highly substituted 4H-pyrans were constructed through a one-pot Sonogashira-nucleophilic cascade reaction in a highly stereochemically controlled fashion [25,26].

### 2.2. In Vitro Evaluation of the Antibacterial Activities

The in vitro antibacterial activities of the synthesized PTM derivatives **A1**–**A10**, **B1**–**B15**, and **C1**–**C7** were evaluated against S. aureus ATCC 29213, two MRSA strains, and two methicillin-sensitive *S. aureus* (MSSA) strains, as well as the Gram-negative pathogens *Klebsiella pneumoniae* and *Escherichia coli*, isolated from local hospitals [27]. All of the tested compounds showed potent antibacterial activities against *S. aureus*, while they had no antibacterial activities against the tested *K. pneumoniae* and *E. coli* strains. Impressively, the MICs of 14 of them were 1 μg/mL against the tested *S. aureus* strains, which was equipotent to PTM. The alkyne derivatives **B1**–**B15** were generally more potent than compounds **A** and **C** series since 11 of them had a MIC of 1 μg/mL while the MICs of the remaining four compounds were 4–8 μg/mL. These α-alkyne PTM derivatives may have less steric hindrance on the interactions between PTM cage moiety and FabF, suggesting that the steric effect at the α-position of PTM was a key factor to affect the antibacterial activity. Except **A5** in series **A**, the acrylic ester analogues **A1**–**A4** (MICs: 1–16 μg/mL) were more potent than the acrylic amide analogues **A6**–**A10** (MICs: 8–64 μg/mL), which indicates that both long alkyl side chain and amides were not favored for the antibacterial activity. The MICs of **C1**–**C7** were 16–32 μg/mL, less potent than compounds in **A** and **B** series. The presence of a 4*H*-pyran moiety in these PTM derivatives is deleterious for the antibacterial activity, which probably changes the conformation of the terpene cage moiety. Therefore, these results suggest that only proper modification of the PTM cage moiety would lead to potent derivatives [11].

### 2.3. Molecular Docking Studies

**Figure 2:**
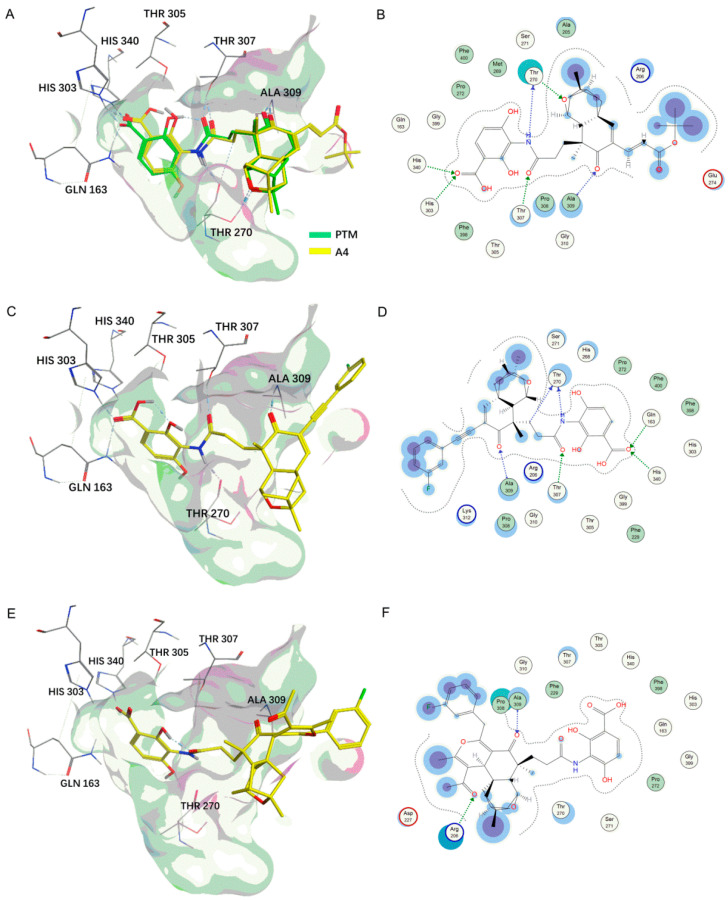
The predicted docking mode of **A4**, **B8**, and **C5** with ecFabF. (**A**) **A4** (yellow) overlaps with PTM (green). (**B**) The interaction map of **A4** with FabF shown in a two-dimensional mode. (**C**–**F**) The docking mode of **B8** and **C5** with ecFabF.

**Figure 3:**
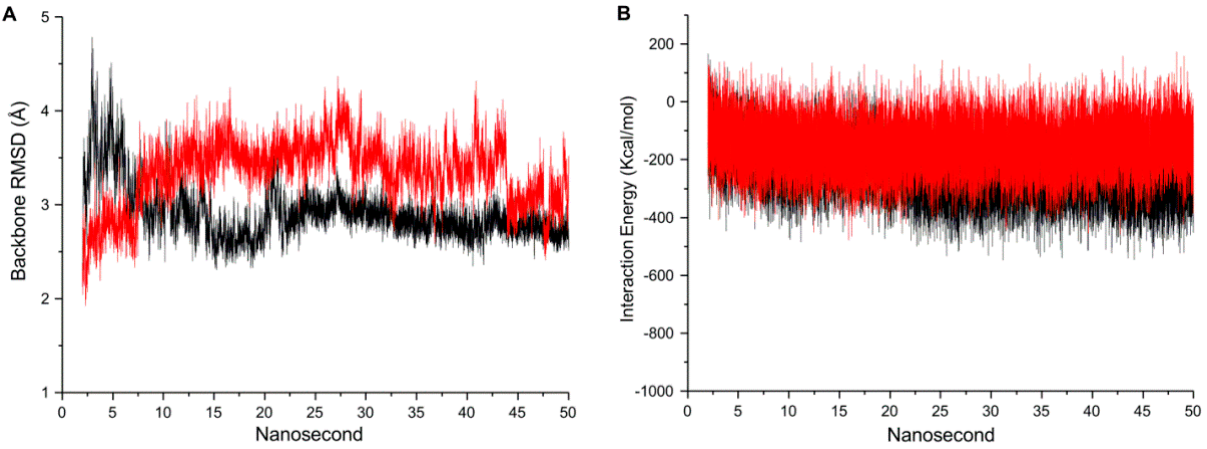
Molecular dynamics simulation. (**A**) The RMSD values of the FabF backbone and (**B**) the nonbond interaction energies between protein and ligand in the complexes between 2 and 50 ns. **A4** was in red and PTM was in black.

We selected representative compounds **A4**, **B8**, and **C5** to study their interactions with the amino acid residues in the active pocket of FabF using the *Escherichia coli* FabF(C163Q)-PTM complex (PDB ID: 2GFX) as a template (Figure 2). All docking experiments were performed using MOE (Molecular Operating Environment) platform based on our previous report [15,16,28]. Although the three compounds could be docked to the active site of FabF, **A4** had the most similar docking interactions with PTM in both the ADHBA or the ketolide part in comparison to **B8** and **C5**. Compound **A4** has several hydrogen-bonding interactions with His303, His 340, Thr270, Thre307, and Ala309 in the FabF active site. In addition, the presence of a tert-butyl group of **A4** may increase its binding affinity to FabF through hydrophobic interactions (Figure 2A and Figure 3B). Compound **B8** also had a PTM-like docking mode with FabF, especially the benzoic acid moiety (Figure 2C and Figure 3D). The predicted binding modes of **A4** and **B8** with FabF suggest that they could bind to FabF effectively. In contrast, the docking mode of **C5** with FabF was quite different from PTM due to the disappearance of most hydrogen-bonding interactions with FabF (Figure 2E and Figure 3F). This suggests that **C5** may bind to FabF poorly, which correlated with its attenuated antibacterial activity (Table 1).

### 2.4. Molecular Dynamics Simulation

To evaluate the stabilities of predicted FabF–ligand complexes under dynamic conditions, a molecular dynamics simulation was next conducted using PTM and **A4** [16,29]. All of the predicted FabF–PTM or FabF–**A4** complexes were finished with 50 ns simulation and their stabilities were evaluated by the RMSD of protein backbone using the generated trajectory data, in comparison with the co-crystal structure of ecFabF(C163Q)–PTM (Scheme 3A). The backbone root mean square deviation (RMSD) values of **A4**–FabF and PTM–FabF complex were similar, which suggested **A4** may bind to FabF and induce similar structural fluctuations with PTM. The binding stabilities of the predicted two complexes were further evaluated by the nonbond interaction energies composed of electrostatics and Van der Waals forces. Compound **A4** had similar nonbond energies with PTM, with the nonbond energy of −151.16 and −232.04 kcal/mol for **A4** and PTM, respectively (Figure 3B). Therefore, compound **A4** and other synthesized analogues may bind to FabF in a similar mode with PTM.

The metabolic stability of selected representative PTM derivatives was next evaluated using pooled human liver microsomes (Table 2) [14]. Compounds **A5**, **A10**, and **B8**, as well as **1b**, exhibited improved stability compared to PTM in human liver microsomes. The unique structure features in these compounds, such as the hydrophobic C-18 alkyl chain in **A5** and the pyrenyl or 3-F substituted phenylethynyl groups in **1b** or **B8**, may contribute to their metabolic stability. The long half-life of **A10** (>120 min) may be partially owing to its mopholine moiety. The 6-iodo PTM (**4**) was equally stable with PTM, while **1a**, **A4**, and **C6** were less stable. The short half-life of **A4** may be due to the hydrolysis of its ester bond despite that it was relatively stable in LB agar (Figure S44). In addition, all of the tested PTM derivatives, except **1a**, had higher plasma protein binding than PTM in the plasma protein binding assay. It is probably consistent with their increased lipophilicity.

### 2.5. Evaluation of the Antibacterial Activities of 4, A4, and B8 in a Mouse Peritonitis Model

**Figure 4:**
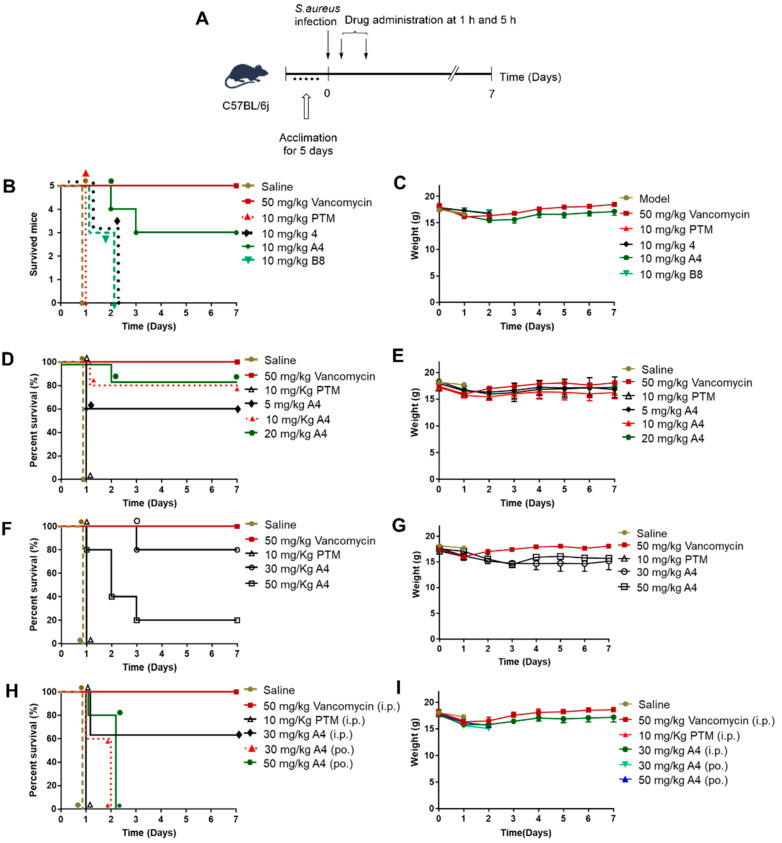
In vivo antimicrobial activity of PTM derivatives. (**A**) The general procedures of the mouse peritonitis model. (**B**) Therapeutic efficacy of compound **4**, **A4**, **B8**, and PTM on MRSA infected mice. Saline was used as the negative control and vancomycin as the antibiotic control. There were five mice in each group. The infected mice were inspected two times and their survival was tracked for 7 days. (**C**) The average weights of each group were recorded once a day. (**D**,**F**,**H**) Therapeutic efficacy of **A4** in different dosages, using the same procedures. (**E**,**G**,**I**) The average weights of each group.

The in vivo antibacterial activity of compounds **4**, **A4**, and **B8** was evaluated in a mouse model of lethal peritonitis using vancomycin and PTM as controls [14,15,16]. The selection of these representative PTM derivatives was based on their potent in vitro anti-staphylococcus activity, various types of modification on PTM ketolide, as well as different behaviors in plasma protein binding and hepatic microsome assays. The C57B/6J mice were inoculated intraperitoneally with 2 × 10^7^ CFU of MRSA, followed by intraperitoneal treatment with double doses of saline or designated antibiotics at 1 h and 5 h after bacterial injection, and inspected for 7 days (Figure 4) [30]. The mice (n = 5) in the saline-treated or PTM-treated groups (10 mg/kg) died after MRSA infection within 24 h, while 100% of the mice survived in the vancomycin-treated group (50 mg/kg). Further, 40% of the mice treated by compounds **4** and **B8** (10 mg/kg) also died within 24 h, while the remaining mice survived within 48 h, suggesting that **4** and **B8** have slightly improved anti-staphylococcus activity over PTM in vivo. Importantly, 60% of the mice in **A4** group (10 mg/kg) were rescued after 7 days, suggesting that **A4** has better in vivo efficacy than PTM (Figure 4B,C).

Next, the in vivo efficacy of **A4** was evaluated in five different dosages (5, 10, 20, 30, and 50 mg/kg) using PTM and vancomycin as controls (Figure 4D,E). Intriguingly, the survival rate of the infected mice treated by **A4** in the dosages of 5, 10, 20, and 30 mg/kg were similar (60%, 60%, 80%, 60%), while only 20% of the mice survived by the treatment of **A4** (50 mg/kg). We observed that the death of the infected mice treated by **A4** in the high dose groups of 30 and 50 mg/kg may be due to its toxicity while unrelated to bacterial infection. Finally, **A4** (30 and 50 mg/kg) was given to the infected mice by oral gavage and most of the treated mice survived within 48 h, suggesting that **A4** also had certain anti-staphylococcus activity in vivo when given by the oral administration. We have previously observed a similar toxicity issue using 6-pyrenyl PTM (**1b**) in a dosage of 50 mg/kg [15]. Taken together, these results suggest that there might be a relatively narrow therapeutic window to treat systematic bacterial infection using PTM derivatives. Further mechanistic study would be needed to delineate if the toxicity is only related to the off-target effects of **A4** and **1b** per se or other PTM derivatives. These results will be reported in due course.

### 2.6. Evaluation of the Antibacterial Activities in Skin Infection Model

MRSA-induced skin infection is a serious issue that threatens the public health. Considering the toxicity of **A4** in systematic treatment in the MRSA-infection model, we next evaluated its efficacy in an MRSA-infected skin model. The reason is that the antibiotics would act on the pathogens of the wounded skin directly and may not enter the systemic circulation, which would reduce its potential toxicity. We have previously evaluated PTM in an MRSA-infection skin model, which was comparable to mupirocin despite its poor pharmacokinetics [14].

**Figure 5:**
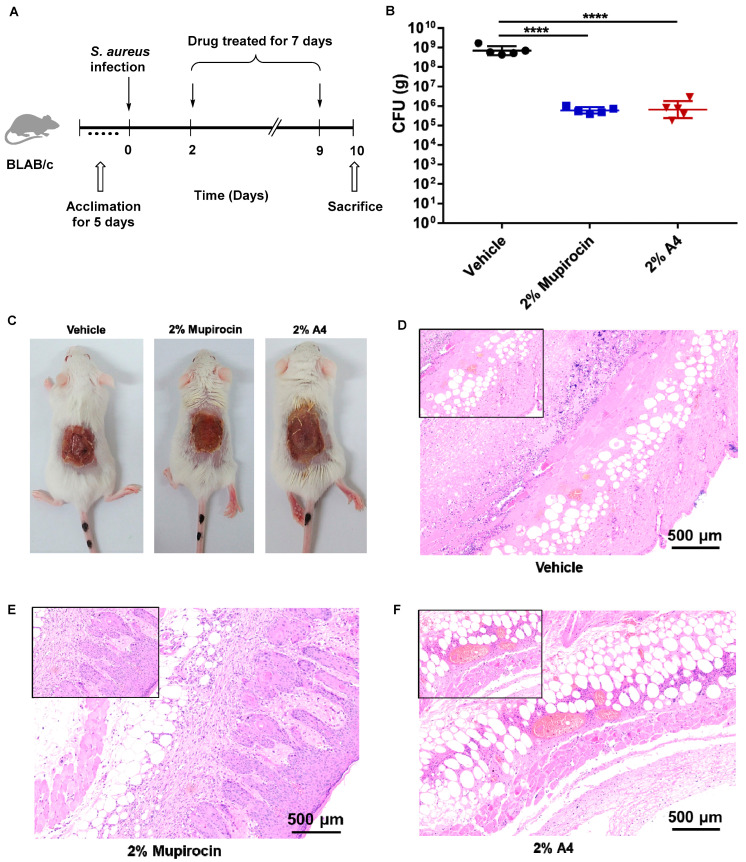
The antibacterial activity of PTM and **A4** in MRSA-induced mice skin infection. (**A**) General procedures for evaluating the therapeutic efficacy of **A4** in a mouse skin infection model. Mupirocin was used as the positive control. The infected mice were treated twice a day for 7 days. (**B**) The total bacterial loads in the skin lesions were determined. Statistical analysis was calculated by the Mann–Whitney test. **** *p* < 0.0001. (**C**) Wounds of BALB/c mice treated and untreated with compounds after 7 days. (**D**–**F**) HE staining histological appearance of *S. aureus*-infected skin lesion on day 10. Biopsy specimens were taken immediately after the termination of the experiment, fixed in formalin, and embedded in paraffin. The biopsy specimens were stained with hematoxylin and eosin. Each point represents data from a single mouse. Mean values are presented; n = 5.

The skin of female BABL/c mice was first infected with MRSA, which then was treated with **A4** (2 mg) or mupirocin (2 mg) twice a day for 7 days (Figure 5A). After the mice were sacrificed, *S. aureus* strains on their treated skin were obtained, cultured, and counted (n = 5/group). Compared to the untreated mice with *S. aureus* loads of (7.9 ± 0.9) × 10^8^ CFU/g, the MRSA on the skin of the mice treated by **A4** was reduced ~ 790-fold with the load of *S. aureus* of (1.0 ± 0.3) × 10^6^ CFU/g (Figure 5B). The mice treated by mupirocin had the load of *S. aureus* of (6.5 ± 2.4) × 10^5^ CFU/g, which was only slightly better than **A4**. We also observed the presence of a secondary scald on the skin of the infected mice, in which the detachment of the epidermis from the dermis was obvious (Figure 5C). In contrast, both mupirocin- and **A4**-treated mice showed the healing of the skin. These observations were consistent with the hematoxylin and eosin (H&E) staining (Figure 5D–F).

## 3. Materials and Methods

### 3.1. General Experimental Procedure

The commercial reagents were used as received. All ^1^H and ^13^C NMR spectra were recorded on a Brucker 500 MHz or 400 MHz spectrometer. Chemical shifts were reported in ppm relative to the internal standard tetramethylsilane (*δ* = 0 ppm) for ^1^H NMR and deuterio chloroform (δ = 77.00 ppm) for ^13^C NMR spectroscopy. The following abbreviations were used to designate chemical shift multiplicities: s = singlet, d =doublet, t = triplet, q = quartet, m = multiplet, br = broad. HRMS spectra were recorded on an Agilent 6500 series Q-TOF instrument. All compounds examined possessed a purity of at least 95%.

### 3.2. Chemistry

#### 3.2.1. Synthesis of 6-Iodo Platensic Acid (**2**)

6-iodo platensic acid ethylester (**1**) was prepared according to our previous report [15]. To a solution of iodo-PTMA ethylester **1** (2.0 g, 4.5 mmol) in methanol/water (10 mL/30 mL) was added 2 M LiOH (6.8 mL, 13.5 mmol). The mixture was stirred at room temperature for 4 h, and then neutralized with 2 M HCl. Methanol was removed in vacuo and then extracted with ethyl acetate (3 × 100 mL). The organic phase was washed with brine and water and dried with anhydrous sodium sulfate. Last, the solvent was distilled under vacuo to give an oil product 6-iodo platensic acid (**2**) with good purity (>95% yield), which was subjected to the next condensation reaction without any purification.

#### 3.2.2. Synthesis of 6-Iodo PTM (**4**)

3-Amino-2,4-dihydroxybenzoic acid (ADHBA) (**3**) was prepared according to the previous report [21]. To a solution of **2** (1.0 g, 2.4 mmol) in anhydrous dichloromethane (DCM)/dimethylformamide (DMF) (20 mL/60 mL) were added trimethylamine (TEA) (1.0 mL, 7.2 mmol) and PyBOP (1.3 g, 2.4 mmol). After the mixture was stirred at room temperature for 5 min, ADHBA (**3**) (486.0 mg, 2.9 mmol) was added and stirred for additional 25 min. Next, the reaction was quenched with 0.1 M HCl and extracted with DCM (3 × 100 mL). The organic phase was washed with 0.1 M HCl (3 × 100 mL), brine (3 × 100 mL), water (3 × 100 mL), and dried with anhydrous sodium sulfate. Last, the solvent was removed under vacuo to give a crude oil product, which was subjected to silica gel column chromatography (eluent: ethylacetate (EtOAc)/petroleum ether (PE)/ acetic acid (AcOH), 60:40:0.25) to give 6-iodo PTM (**4**) (980.0 mg, 72% yield).

#### 3.2.3. General Procedure for Synthesis of Heck Reaction Products (**A1**–**A10**)

To a stirred solution of **4** (100.0 mg, 0.176 mmol) in CH_3_CN (15 mL) were sequentially added ethyl acrylate (0.88 mmol, 5.0 molar equiv), TEA (35.0 μL, 0.264 mmol, 1.5 molar equiv), Pd(OAc)_2_ (3.8 mg, 0.017 mmol, 0.1 molar equiv), and Ph_3_P (4.5 mg, 0.017 mmol). After stirring at 80 °C for 2 h, the reaction mixture was worked up by dilution with brine and extracted with EtOAc. After drying and evaporation under vacuo, the residue was subjected to silica gel column chromatography (eluent: EtOAc/PE/AcOH 60:40:0.25 to EtOAc/AcOH 100:0.25) to the corresponding products.

#### 3.2.4. General Procedure for Synthesis of Sonogashira Reaction Products (**B1**–**B12**)

To a stirred solution of **4** (100.0 mg, 0.176 mmol) in tetrahydrofuran (THF) (15 mL) were added phenylacetylene (0.264 mmol, 1.5 molar equiv), diisopropylamine (DIPA) (63 μL), CuI (4.0 mg, 0.02 mmol, 0.1 molar equiv), and Pd(PPh_3_)_2_Cl_2_ (10.0 mg, 0.02 mmol, 0.1 molar equiv). After stirring for 4 h at room temperature, the reaction was worked up by dilution with brine and extraction with EtOAc. The organic phase was dried and evaporated, and the residue was purified by silica gel column chromatography (eluent: EtOAc/PE/AcOH 60:40:0.25 to EtOAc/AcOH 100:0.25) to the corresponding products.

#### 3.2.5. General Procedure for Preparing One-Pot Synthesis Products (**C1**–**C7**)

To a stirred solution of **4** (100.0 mg, 0.176 mmol) in dry DMF (10 mL) were added alkyne (0.264 mmol, 1.5 molar equiv), N,N-Diisopropylethylamine (DIPEA) (50 μL), AgOAc (7.0 mg, 0.04 mmol, 0.2 molar equiv), and Pd(PPh3)_2_Cl_2_ (10.0 mg, 0.02 mmol, 0.1 molar equiv). After stirring for 30 min at room temperature, acetylacetone (36.0 mg, 0.352 mmol, 2.0 molar equiv) and cesium carbonate (287.0 mg, 0.88 mmol, 5.0 molar equiv) were added. The mixture was then stirred under 90 °C for 5 h. Finally, the reaction was worked up by dilution with brine, acidified with 2 M HCl, and extracted with EtOAc. The organic phase was dried and evaporated, and the residue was purified by silica gel column chromatography (eluent: EtOAc/PE/AcOH 60:40:0.25 to EtOAc/AcOH 100:0.25) to the corresponding products.

#### 3.2.6. Characterization Data of **2**, **4**, **A1**–**A10**, **B1**–**B15**, and **C1**–**C7**

##### Characterization Data of **2**

^1^H NMR (500 MHz, Chloroform-*d*) δ 7.27 (s, 1H), 4.38 (s, 1H), 2.40 (dd, *J* = 12.2, 5.0 Hz, 2H), 2.33–2.27 (m, 2H), 2.24–2.17 (m, 1H), 2.06–2.03 (m, 2H), 1.96 (s, 1H), 1.90 (dd, *J* = 11.2, 3.3 Hz, 1H), 1.76 (ddd, *J* = 20.9, 12.6, 8.8 Hz, 2H), 1.60 (dd, *J* = 11.3, 4.1 Hz, 1H), 1.41 (s, 3H), 1.22 (s, 3H).

^13^C NMR (126 MHz, CDCl_3_) δ 196.23, 178.18, 162.36, 102.25, 87.11, 76.33, 54.48, 50.17, 47.03, 45.83, 44.32, 42.89, 40.32, 31.37, 28.90, 24.55, 22.83.

HRMS (ESI) *m/z* calculated for C_17_H_22_IO_4_, [M + H]^+^ 417.0563; Found: 417.0564.

##### Characterization Data of **4**

^1^H NMR (500 MHz, Chloroform-*d*) δ 11.66 (s, 1H), 11.09 (s, 1H), 8.03 (s, 1H), 7.60 (d, *J* = 8.9 Hz, 1H), 7.35 (s, 1H), 6.50 (d, *J* = 8.9 Hz, 1H), 4.63 (s, 1H), 2.72 (ddd, *J* = 15.7, 12.0, 4.6 Hz, 1H), 2.60 (s, 1H), 2.54 (q, *J* = 8.7, 6.9 Hz, 2H), 2.49–2.42 (m, 1H), 2.17 (dd, *J* = 12.0, 4.1 Hz, 2H), 2.07 (s, 1H), 2.05–2.01 (m, 1H), 1.93–1.82 (m, 2H), 1.71 (d, *J* = 11.5 Hz, 1H), 1.53 (s, 3H), 1.33 (s, 3H).

^13^C NMR (126 MHz, CDCl_3_) δ 196.54, 173.14, 172.52, 162.11, 155.00, 154.11, 128.26, 114.21, 111.12, 103.70, 102.07, 88.03, 76.66, 54.37, 50.25, 47.18, 45.74, 44.43, 42.68, 40.18, 31.71, 31.00, 24.55, 22.56.

HRMS (ESI) *m/z* calculated for C_24_H_27_IO_7_, [M + H]^+^ 568.0832; Found: 568.0822.

##### Characterization Data of **A1**

^1^H NMR (500 MHz, CDCl_3_) δ 8.08 (s, 1H), 7.59 (d, *J* = 9.0 Hz, 1H), 7.37 (d, *J* = 16.0 Hz, 1H), 6.74 (s, 1H), 6.59 (d, *J* = 16.1 Hz, 1H), 6.48 (d, *J* = 8.9 Hz, 1H), 4.64 (s, 1H), 3.76 (s, 3H), 2.76–2.63 (m, 1H), 2.53 (dt, *J* = 13.8, 6.8 Hz, 3H), 2.16 (ddd, *J* = 15.4, 12.2, 4.6 Hz, 2H), 2.06 (d, *J* = 10.4 Hz, 2H), 2.01 (dd, *J* = 11.4, 3.3 Hz, 1H), 1.85 (ddd, *J* = 28.9, 13.7, 6.7 Hz, 2H), 1.74 (d, *J* = 11.4 Hz, 1H), 1.53 (s, 3H), 1.29 (s, 3H).

^13^C NMR (126 MHz, CDCl_3_) δ 200.94, 173.39, 172.36, 167.46, 154.96, 154.42, 154.12, 139.40, 131.37, 128.21, 121.16, 114.22, 111.05, 103.79, 88.43, 76.71, 54.81, 51.75, 46.85, 46.34, 45.32, 44.74, 43.24, 40.27, 31.09, 30.97, 23.96, 22.58.

HRMS (ESI) *m/z* calculated for C_28_H_32_NO_9_, [M + H]^+^ 526.2077; Found: 526.2073.

##### Characterization Data of **A2**

^1^H NMR (500 MHz, MeOD) δ 7.67 (d, *J* = 8.4 Hz, 1H), 7.45 (d, *J* = 16.1 Hz, 1H), 7.00 (s, 1H), 6.65 (d, *J* = 16.1 Hz, 1H), 6.42 (d, *J* = 8.6 Hz, 1H), 4.54 (s, 1H), 4.32–4.14 (m, 2H), 3.88–3.72 (m, 2H), 2.48 (d, *J* = 9.9 Hz, 2H), 2.43 (dd, *J* = 11.1, 4.4 Hz, 1H), 2.40–2.32 (m, 1H), 2.16–2.12 (m, 1H), 2.11 (s, 2H), 1.92 (dt, *J* = 13.7, 5.1 Hz, 2H), 1.86 (td, *J* = 6.9, 3.5 Hz, 2H), 1.82 (d, *J* = 11.1 Hz, 1H), 1.46 (s, 3H), 1.28 (s, 3H).

^13^C NMR (126 MHz, MeOD) δ 201.52, 174.26, 167.35, 155.80, 139.91, 130.88, 129.10, 120.02, 112.49, 107.72, 87.52, 76.59, 65.71, 59.69, 54.50, 46.72, 46.19, 45.43, 44.73, 42.79, 40.06, 31.53, 30.58, 23.24, 21.82.

HRMS (ESI) *m/z* calculated for C_29_H_34_NO_10_, [M + H]^+^ 556.2183; Found: 556.2182.

##### Characterization Data of **A3**

^1^H NMR (500 MHz, CDCl_3_) δ 8.06 (s, 1H), 7.60 (d, *J* = 8.8 Hz, 1H), 7.37 (d, *J* = 16.0 Hz, 1H), 6.74 (s, 1H), 6.59 (d, *J* = 16.1 Hz, 1H), 6.49 (d, *J* = 8.9 Hz, 1H), 4.62 (s, 1H), 4.21–4.10 (m, 2H), 2.68 (ddd, *J* = 15.2, 11.9, 4.9 Hz, 1H), 2.53 (d, *J* = 5.9 Hz, 1H), 2.49 (d, *J* = 16.7 Hz, 2H), 2.17 (ddd, *J* = 20.3, 11.4, 4.2 Hz, 2H), 2.07 (d, *J* = 8.1 Hz, 2H), 2.00 (d, *J* = 11.3 Hz, 1H), 1.90–1.80 (m, 2H), 1.73 (d, *J* = 11.3 Hz, 1H), 1.65 (p, *J* = 6.7 Hz, 2H), 1.53 (s, 3H), 1.46–1.39 (m, 2H), 1.30 (s, 4H), 0.94 (t, *J* = 7.3 Hz, 3H).

^13^C NMR (126 MHz, CDCl_3_) δ 201.03, 173.31, 171.22, 167.06, 154.85, 154.24, 139.00, 132.10, 131.44, 128.55, 128.20, 121.69, 114.20, 110.97, 88.25, 76.66, 64.44, 54.86, 46.85, 46.34, 45.40, 44.73, 43.28, 40.32, 31.21, 31.07, 30.69, 23.93, 22.64, 19.15, 13.71.

HRMS (ESI) *m/z* calculated for C_31_H_38_NO_9_, [M + H]^+^ 568.2547; Found: 568.2545.

##### Characterization Data of **A4**

^1^H NMR (500 MHz, CDCl_3_) δ 11.68 (s, 1H), 11.12 (s, 1H), 8.07 (s, 1H), 7.60 (d, *J* = 8.9 Hz, 1H), 7.28 (d, *J* = 16.1 Hz, 1H), 6.72 (s, 1H), 6.51 (d, *J* = 3.8 Hz, 1H), 6.48 (d, *J* = 3.3 Hz, 1H), 4.63 (s, 1H), 2.68 (ddd, *J* = 14.4, 12.2, 4.9 Hz, 1H), 2.57–2.53 (m, 1H), 2.51 (d, *J* = 8.6 Hz, 2H), 2.49–2.43 (m, 1H), 2.17 (ddd, *J* = 21.1, 11.4, 7.3 Hz, 2H), 2.06 (d, *J* = 8.5 Hz, 1H), 1.99 (dd, *J* = 11.5, 3.6 Hz, 1H), 1.90–1.80 (m, 2H), 1.73 (d, *J* = 11.4 Hz, 1H), 1.53 (s, 3H), 1.49 (s, 9H), 1.29 (s, 3H).

^13^C NMR (126 MHz, CDCl_3_) δ 201.12, 173.39, 172.51, 166.24, 155.02, 154.14, 153.62, 137.92, 131.59, 128.21, 123.56, 114.24, 111.10, 103.74, 88.35, 80.62, 76.70, 54.84, 46.83, 46.28, 45.38, 44.73, 43.25, 40.29, 31.18, 31.07, 28.11, 23.95, 22.61.

HRMS (ESI) *m/z* calculated for C_31_H_37_NNaO_9_, [M+Na]^+^ 590.2366; Found: 590.2369

##### Characterization Data of **A5**

^1^H NMR (500 MHz, CDCl_3_) δ 8.07 (s, 1H), 7.59 (d, *J* = 9.0 Hz, 1H), 7.36 (d, *J* = 16.0 Hz, 1H), 6.74 (s, 1H), 6.59 (d, *J* = 16.0 Hz, 1H), 6.51–6.43 (m, 1H), 4.64 (s, 1H), 4.18–4.10 (m, 2H), 2.67 (d, *J* = 14.5 Hz, 1H), 2.54 (s, 1H), 2.51 (s, 2H), 2.47 (d, *J* = 3.5 Hz, 1H), 2.17 (ddd, *J* = 20.6, 12.0, 4.2 Hz, 2H), 2.08 (d, *J* = 9.3 Hz, 1H), 2.00 (d, *J* = 10.2 Hz, 1H), 1.86 (td, *J* = 12.7, 5.4 Hz, 2H), 1.74 (d, *J* = 11.4 Hz, 1H), 1.68–1.63 (m, 2H), 1.53 (s, 3H), 1.28 (d, *J* = 17.0 Hz, 35H), 0.88 (t, *J* = 6.8 Hz, 3H).

^13^C NMR (126 MHz, CDCl_3_) δ 200.96, 173.36, 172.34, 167.13, 154.94, 154.23, 154.12, 139.05, 131.44, 128.21, 121.68, 114.20, 111.02, 103.85, 88.41, 76.71, 64.80, 54.83, 46.84, 46.33, 45.36, 44.74, 43.25, 40.29, 31.92, 31.13, 31.00, 29.69, 29.65, 29.60, 29.53, 29.36, 29.28, 28.67, 25.93, 23.95, 22.69, 22.58, 14.13.

##### Characterization Data of **A6**

^1^H NMR (400 MHz, MeOD) δ 7.68 (d, *J* = 8.8 Hz, 1H), 7.27 (d, *J* = 15.8 Hz, 1H), 6.91 (s, 1H), 6.78 (d, *J* = 15.9 Hz, 1H), 6.46 (d, *J* = 8.9 Hz, 1H), 4.54 (s, 1H), 3.33 (t, *J* = 1.7 Hz, 2H), 2.48 (d, *J* = 5.4 Hz, 2H), 2.41 (dd, *J* = 9.1, 4.7 Hz, 1H), 2.39–2.31 (m, 1H), 2.12 (s, 2H), 2.03 (s, 1H), 2.01 (s, 1H), 1.97–1.92 (m, 1H), 1.92–1.86 (m, 2H), 1.82 (d, *J* = 11.2 Hz, 1H), 1.47 (s, 3H), 1.30 (s, 3H).

^13^C NMR (101 MHz, MeOD) δ 201.67, 174.35, 172.19, 169.68, 169.68, 158.11, 154.88, 136.47, 131.17, 129.16, 122.71, 112.44, 108.07, 104.69, 87.49, 76.63, 54.52, 46.73, 46.10, 45.48, 44.72, 42.78, 40.04, 31.48, 30.48, 23.23, 21.79.

HRMS (ESI) *m/z* calculated for C_27_H_31_N_2_O_8_, [M + H]^+^ 511.2080; Found: 511.2079.

##### Characterization Data of **A7**

^1^H NMR (500 MHz, CDCl_3_) δ 8.12 (s, 1H), 7.61 (d, *J* = 8.9 Hz, 1H), 7.35 (d, *J* = 15.4 Hz, 1H), 7.17 (d, *J* = 15.4 Hz, 1H), 6.66 (s, 1H), 6.51 (d, *J* = 8.9 Hz, 1H), 4.61 (s, 1H), 3.16 (s, 3H), 3.06 (s, 3H), 2.74–2.60 (m, 1H), 2.52 (d, *J* = 6.1 Hz, 1H), 2.49 (t, *J* = 6.8 Hz, 2H), 2.22–2.12 (m, 2H), 2.09–2.01 (m, 2H), 1.98 (dd, *J* = 11.5, 3.4 Hz, 1H), 1.90–1.82 (m, 2H), 1.73 (d, *J* = 11.4 Hz, 1H), 1.53 (s, 3H), 1.30 (s, 3H).

^13^C NMR (126 MHz, CDCl_3_) δ 201.84, 173.54, 172.67, 167.64, 156.43, 155.00, 154.30, 138.04, 131.33, 128.23, 121.00, 114.27, 111.02, 104.04, 88.06, 76.60, 54.84, 47.18, 46.38, 45.39, 44.70, 43.31, 40.38, 37.61, 36.09, 31.40, 31.35, 23.93, 22.71.

HRMS (ESI) *m/z* calculated for C_29_H_35_N_2_O_8_, [M + H]^+^ 539.2393; Found: 539.2396.

##### Characterization Data of **A8**

^1^H NMR (500 MHz, MeOD) δ 7.67 (d, *J* = 8.9 Hz, 1H), 7.22 (d, *J* = 15.8 Hz, 1H), 6.87 (s, 1H), 6.72 (d, *J* = 15.8 Hz, 1H), 6.46 (d, *J* = 8.9 Hz, 1H), 4.53 (t, *J* = 3.0 Hz, 1H), 3.37 (s, 1H), 3.33 (p, *J* = 1.7 Hz, 1H), 2.50–2.45 (m, 2H), 2.45–2.31 (m, 2H), 2.15–2.07 (m, 3H), 2.01 (s, 4H), 1.95–1.85 (m, 3H), 1.81 (d, *J* = 11.1 Hz, 1H), 1.46 (s, 3H), 1.29 (d, *J* = 7.9 Hz, 3H), 1.19 (s, 3H), 1.18 (s, 3H).

^13^C NMR (126 MHz, MeOD) δ 201.74, 174.34, 173.80, 172.22, 166.38, 157.98, 154.49, 135.26, 131.32, 129.13, 123.53, 112.50, 108.11, 104.79, 87.48, 76.64, 54.54, 46.75, 46.07, 45.51, 44.72, 42.81, 41.20, 40.05, 31.53, 30.52, 23.25, 21.82, 21.23, 19.36.

HRMS (ESI) *m/z* calculated for C_30_H_37_N_2_O_8_, [M + H]^+^ 553.2550; Found: 553.2548.

##### Characterization Data of **A9**

^1^H NMR (500 MHz, CDCl_3_) δ 8.15 (s, 1H), 7.59 (d, *J* = 8.9 Hz, 1H), 7.08 (d, *J* = 15.6 Hz, 1H), 6.74 (d, *J* = 15.6 Hz, 1H), 6.63 (s, 1H), 6.48 (d, *J* = 8.9 Hz, 1H), 5.78 (s, 1H), 4.57 (s, 1H), 2.64 (dd, *J* = 17.7, 8.6 Hz, 1H), 2.50 (d, *J* = 6.5 Hz, 1H), 2.48 (d, *J* = 5.3 Hz, 1H), 2.44 (d, *J* = 10.2 Hz, 2H), 2.20–2.11 (m, 1H), 2.11–2.08 (m, 1H), 2.03 (d, *J* = 11.6 Hz, 1H), 1.98–1.91 (m, 1H), 1.87–1.78 (m, 2H), 1.69 (d, *J* = 11.3 Hz, 1H), 1.50 (s, 3H), 1.39 (s, 9H), 1.26 (s, 3H).

^13^C NMR (126 MHz, CDCl_3_) δ 201.85, 173.57, 172.54, 165.94, 155.59, 154.95, 154.29, 135.31, 131.22, 128.25, 125.64, 114.24, 110.97, 104.06, 88.10, 76.59, 58.36, 54.81, 51.57, 47.07, 46.30, 45.42, 44.69, 43.28, 40.36, 31.41, 28.74, 23.92, 22.68.

HRMS (ESI) *m/z* calculated for C_31_H_39_N_2_O_8_, [M + H]^+^ 567.2706; Found: 567.2711.

##### Characterization Data of **A10**

^1^H NMR (500 MHz, DMSO-*d*_6_) δ 9.10 (s, 1H), 7.52 (d, *J* = 8.7 Hz, 1H), 7.26 (d, *J* = 15.5 Hz, 1H), 7.17 (s, 1H), 7.15 (d, *J* = 15.6 Hz, 1H), 6.31 (d, *J* = 8.6 Hz, 1H), 4.41 (s, 1H), 3.56 (d, *J* = 24.9 Hz, 8H), 2.39 (q, *J* = 6.3 Hz, 2H), 2.32 (s, 1H), 2.20–2.09 (m, 2H), 2.00 (dd, *J* = 11.5, 4.5 Hz, 2H), 1.96–1.90 (m, 1H), 1.84 (dd, *J* = 11.9, 6.9 Hz, 1H), 1.79 (dd, *J* = 11.4, 3.0 Hz, 1H), 1.74 (d, *J* = 10.5 Hz, 2H), 1.38 (s, 3H), 1.17 (s, 3H).

^13^C NMR (126 MHz, DMSO-*d*_6_) δ 201.99, 172.62, 172.45, 165.01, 159.55, 154.86, 136.91, 130.95, 130.10, 128.96, 119.74, 113.42, 113.31, 107.18, 87.00, 75.98, 66.80, 66.64, 54.98, 46.69, 46.26, 45.56, 44.75, 43.26, 32.05, 30.82, 30.78, 24.41, 23.40.

HRMS (ESI) *m/z* calculated for C_31_H_37_N_2_O_9_, [M + H]^+^ 581.2499; Found: 581.2500.

##### Characterization Data of **B1**

^1^H NMR (500 MHz, Chloroform-*d*) δ 8.13 (s, 1H), 7.60 (d, *J* = 8.9 Hz, 1H), 6.92 (s, 1H), 6.49 (d, *J* = 8.9 Hz, 1H), 4.73–4.49 (m, 1H), 3.13 (s, 1H), 2.64 (ddd, *J* = 16.6, 13.1, 4.8 Hz, 1H), 2.52 (d, *J* = 6.5 Hz, 1H), 2.47 (d, *J* = 14.2 Hz, 2H), 2.43 (dd, *J* = 7.1, 4.4 Hz, 1H), 2.20–2.12 (m, 1H), 2.05 (d, *J* = 11.9 Hz, 1H), 1.98 (dd, *J* = 11.4, 3.5 Hz, 1H), 1.91 (d, *J* = 5.6 Hz, 1H), 1.86 (dq, *J* = 16.4, 6.8, 5.8 Hz, 2H), 1.69 (d, *J* = 11.5 Hz, 1H), 1.50 (s, 3H), 1.31 (s, 3H).

^13^C NMR (126 MHz, CDCl_3_) δ 199.84, 173.45, 172.92, 158.56, 155.12, 154.28, 128.33, 121.55, 114.23, 111.13, 103.77, 88.05, 80.87, 77.96, 76.49, 54.71, 46.68, 46.24, 45.24, 44.65, 43.09, 40.30, 31.27, 31.22, 24.14, 22.65.

HRMS (ESI) *m/z* calculated for C_26_H_28_NO_7_, [M + H]^+^ 466.1886; Found: 466.1864.

##### Characterization Data of **B2**

^1^H NMR (400 MHz, CDCl_3_) δ 11.78 (s, 1H), 11.16 (s, 1H), 8.12 (s, 1H), 7.61 (d, *J* = 8.9 Hz, 1H), 6.77 (s, 1H), 6.50 (d, *J* = 9.0 Hz, 1H), 4.62 (s, 1H), 2.64 (t, *J* = 7.5 Hz, 1H), 2.51 (s, 2H), 2.40 (t, *J* = 7.1 Hz, 2H), 2.17–2.14 (m, 1H), 2.12 (d, *J* = 4.8 Hz, 1H), 2.08–2.01 (m, 2H), 1.97 (dd, *J* = 11.4, 3.2 Hz, 1H), 1.84 (dd, *J* = 12.3, 7.0 Hz, 1H), 1.68 (d, *J* = 11.4 Hz, 1H), 1.60–1.55 (m, 2H), 1.51 (s, 3H), 1.47–1.44 (m, 2H), 1.33 (s, 3H), 1.27 (s, 2H), 0.93 (t, *J* = 7.3 Hz, 3H).

^13^C NMR (101 MHz, CDCl_3_) δ 200.87, 173.45, 172.50, 155.99, 154.99, 154.21, 128.17, 122.65, 114.25, 111.03, 103.81, 94.50, 87.98, 74.70, 54.85, 46.64, 46.16, 45.32, 44.68, 43.17, 40.32, 31.42, 31.32, 30.59, 24.27, 22.71, 22.06, 19.15, 13.65.

HRMS (ESI) *m/z* calculated for C_30_H_36_NO_7_, [M + H]^+^ 522.2492; Found: 522.2489.

##### Characterization Data of **B3**

^1^H NMR (500 MHz, CDCl_3_) δ 11.86 (s, 1H), 8.16 (s, 1H), 7.61 (d, *J* = 8.9 Hz, 1H), 6.78 (s, 1H), 6.50 (d, *J* = 9.0 Hz, 1H), 4.61 (s, 1H), 2.60 (dd, *J* = 34.1, 10.4 Hz, 2H), 2.50 (d, *J* = 6.7 Hz, 2H), 2.47–2.38 (m, 1H), 2.13 (d, *J* = 14.2 Hz, 2H), 2.05 (d, *J* = 12.1 Hz, 2H), 1.96 (dd, *J* = 11.4, 2.9 Hz, 1H), 1.89 (dd, *J* = 13.1, 4.3 Hz, 1H), 1.84 (dd, *J* = 11.7, 6.9 Hz, 1H), 1.67 (d, *J* = 11.3 Hz, 1H), 1.51 (s, 3H), 1.33 (s, 3H), 1.30 (s, 9H).

^13^C NMR (126 MHz, CDCl_3_) δ 201.06, 173.48, 172.37, 156.20, 154.95, 154.23, 128.16, 122.52, 114.24, 110.99, 103.91, 102.29, 87.91, 76.51, 73.20, 54.84, 46.59, 46.21, 45.34, 44.67, 43.18, 40.34, 31.47, 31.28, 30.80, 28.88, 24.23, 22.73.

HRMS (ESI) *m/z* calculated for C_30_H_36_NO_7_, [M + H]^+^ 522.2492;Found: 522.2488.

##### Characterization Data of **B4**

^1^H NMR (400 MHz, CDCl_3_) δ 8.14 (s, 1H), 7.62 (d, *J* = 9.0 Hz, 1H), 6.76 (s, 1H), 6.51 (d, *J* = 8.9 Hz, 1H), 4.60 (s, 1H), 2.64 (td, *J* = 13.7, 12.8, 4.8 Hz, 1H), 2.58–2.51 (m, 1H), 2.49 (d, *J* = 7.0 Hz, 2H), 2.43 (dd, *J* = 12.7, 4.0 Hz, 1H), 2.20–2.11 (m, 2H), 2.07 (s, 1H), 2.03 (s, 1H), 1.95 (dd, *J* = 11.5, 3.3 Hz, 1H), 1.92–1.86 (m, 1H), 1.83 (dd, *J* = 12.0, 6.8 Hz, 1H), 1.67 (d, *J* = 11.4 Hz, 1H), 1.50 (s, 3H), 1.31 (s, 3H), 0.84 (ddt, *J* = 11.5, 5.1, 2.7 Hz, 4H).

^13^C NMR (126 MHz, CDCl_3_) δ 200.99, 173.48, 172.69, 156.13, 155.06, 154.25, 128.20, 122.49, 114.26, 111.08, 103.78, 97.50, 87.94, 76.51, 69.99, 54.84, 46.64, 46.17, 45.29, 44.66, 43.18, 40.33, 31.48, 31.35, 24.24, 22.72, 8.68, 8.68.

HRMS (ESI) *m/z* calculated for C_29_H_32_NO_7_, [M + H]^+^ 506.2179; Found: 506.2176.

##### Characterization Data of **B5**

^1^H NMR (500 MHz, CDCl_3_) δ 11.76 (s, 1H), 11.15 (s, 1H), 8.12 (s, 1H), 7.60 (d, *J* = 8.9 Hz, 1H), 6.76 (s, 1H), 6.50 (d, *J* = 8.9 Hz, 1H), 4.61 (s, 1H), 2.69–2.59 (m, 1H), 2.49 (d, *J* = 4.5 Hz, 2H), 2.47–2.42 (m, 1H), 2.38 (t, *J* = 7.3 Hz, 2H), 2.11 (s, 1H), 2.06 (s, 1H), 1.98–1.94 (m, 1H), 1.91 (dd, *J* = 13.4, 4.6 Hz, 1H), 1.83 (dd, *J* = 11.9, 7.0 Hz, 1H), 1.68 (d, *J* = 11.4 Hz, 1H), 1.61–1.57 (m, 2H), 1.50 (s, 3H), 1.46 (s, 2H), 1.39 (d, *J* = 7.9 Hz, 2H), 1.32 (s, 3H), 1.27 (s, 2H), 0.90 (t, *J* = 7.1 Hz, 3H).

^13^C NMR (101 MHz, CDCl_3_) δ 200.86, 173.46, 172.60, 156.00, 155.02, 154.23, 128.18, 122.64, 114.26, 111.03, 103.79, 94.55, 87.96, 87.04, 76.55, 74.68, 54.85, 46.64, 46.16, 45.33, 44.67, 43.18, 40.33, 31.45, 31.35, 31.16, 28.25, 24.26, 22.72, 22.22, 19.44, 13.99.

HRMS (ESI) *m/z* calculated for C_31_H_38_NO_7_, [M + H]^+^ 536.2648; Found: 536.2646.

##### Characterization Data of **B6**

^1^H NMR (500 MHz, CDCl_3_) δ 8.20 (s, 1H), 7.58 (d, *J* = 8.9 Hz, 1H), 6.81 (s, 1H), 6.48 (d, *J* = 8.8 Hz, 1H), 4.58 (s, 1H), 3.49 (s, 1H), 2.64–2.56 (m, 1H), 2.50 (d, *J* = 6.2 Hz, 1H), 2.49–2.41 (m, 2H), 2.19–2.11 (m, 2H), 2.10 (s, 2H), 2.03 (d, *J* = 11.7 Hz, 1H), 1.97 (d, *J* = 10.9 Hz, 2H), 1.92–1.78 (m, 2H), 1.77–1.69 (m, 2H), 1.67 (d, *J* = 11.4 Hz, 2H), 1.62 (d, *J* = 12.2 Hz, 2H), 1.57 (d, *J* = 6.6 Hz, 2H), 1.50 (s, 3H), 1.30 (s, 3H).

^13^C NMR (126 MHz, CDCl_3_) δ 200.67, 173.55, 172.50, 157.10, 155.05, 154.35, 128.24, 121.90, 114.23, 111.08, 103.90, 96.68, 87.97, 78.37, 76.44, 68.97, 54.74, 50.63, 46.64, 46.23, 45.23, 44.66, 43.17, 40.33, 39.74, 39.66, 31.40, 31.22, 25.18, 24.20, 23.28, 22.71.

HRMS (ESI) *m/z* calculated for C_32_H_36_NO_7_, [M + H]^+^ 546.2492; Found: 546.2489.

##### Characterization Data of **B7**

^1^H NMR (400 MHz, CDCl_3_) δ 11.80 (s, 1H), 11.18 (s, 1H), 8.15 (s, 1H), 7.57 (d, *J* = 8.9 Hz, 1H), 7.54 (dd, *J* = 6.5, 3.2 Hz, 2H), 7.35 (d, *J* = 2.1 Hz, 2H), 7.33 (d, *J* = 1.7 Hz, 1H), 6.95 (s, 1H), 6.50 (d, *J* = 9.0 Hz, 1H), 4.66 (s, 1H), 2.75–2.64 (m, 1H), 2.57 (dd, *J* = 10.4, 5.0 Hz, 2H), 2.54–2.47 (m, 1H), 2.24–2.15 (m, 2H), 2.14–2.06 (m, 2H), 2.03 (dd, *J* = 11.4, 3.4 Hz, 1H), 1.99–1.85 (m, 2H), 1.74 (d, *J* = 11.4 Hz, 1H), 1.54 (s, 3H), 1.38 (s, 3H).

^13^C NMR (126 MHz, CDCl_3_) δ 200.42, 176.28, 173.37, 172.57, 156.97, 155.06, 154.22, 131.81, 128.70, 128.30, 128.21, 122.42, 114.25, 111.07, 103.74, 93.00, 88.05, 83.53, 76.54, 54.92, 46.76, 46.41, 45.34, 44.73, 43.28, 40.36, 31.43, 24.24, 22.71, 20.62.

HRMS (ESI) *m/z* calculated for C_32_H_32_NO_7_, [M + H]^+^ 542.2179; Found: 542.2177.

##### Characterization Data of **B8**

^1^H NMR (500 MHz, CDCl_3_) δ 8.14 (s, 1H), 7.58 (d, *J* = 9.0 Hz, 1H), 7.29 (d, *J* = 7.2 Hz, 2H), 7.23 (d, *J* = 9.1 Hz, 1H), 7.05 (t, *J* = 7.0 Hz, 1H), 6.96 (s, 1H), 6.49 (d, *J* = 8.9 Hz, 1H), 4.66 (s, 1H), 2.75–2.64 (m, 1H), 2.55 (d, *J* = 8.5 Hz, 2H), 2.54–2.47 (m, 1H), 2.18 (d, *J* = 11.4 Hz, 2H), 2.10 (d, *J* = 14.0 Hz, 1H), 2.06 (s, 1H), 2.05–1.99 (m, 1H), 1.98–1.86 (m, 2H), 1.74 (d, *J* = 11.3 Hz, 1H), 1.53 (s, 3H), 1.37 (s, 3H).

^13^C NMR (126 MHz, CDCl_3_) δ 173.33, 172.34, 163.25, 161.29, 157.64, 154.96, 154.19, 129.96, 127.70, 127.68, 124.35, 122.12, 118.46, 115.99, 114.21, 111.02, 103.85, 91.65, 91.62, 88.16, 84.41, 76.53, 54.87, 46.75, 46.44, 45.28, 44.72, 43.24, 40.32, 31.35, 31.29, 24.22, 22.66.

HRMS (ESI) *m/z* calculated for C_32_H_31_FNO_7_, [M + H]^+^ 560.2085; Found: 560.2083.

##### Characterization Data of **B9**

^1^H NMR (500 MHz, CDCl_3_) δ 8.18 (s, 1H), 7.69 (dd, *J* = 12.2, 7.4 Hz, 1H), 7.59 (d, *J* = 8.7 Hz, 1H), 7.49 (t, *J* = 7.6 Hz, 1H), 7.10 (t, *J* = 7.8 Hz, 1H), 6.91 (s, 1H), 6.86 (s, 1H), 6.67 (d, *J* = 7.9 Hz, 1H), 6.49 (d, *J* = 8.9 Hz, 1H), 4.61 (s, 1H), 2.64 (d, *J* = 10.2 Hz, 1H), 2.51 (d, *J* = 6.8 Hz, 2H), 2.20–2.12 (m, 2H), 2.10 (s, 1H), 2.06 (s, 2H), 2.01–1.97 (m, 1H), 1.94 (dd, *J* = 12.9, 4.0 Hz, 1H), 1.91–1.84 (m, 1H), 1.71 (d, *J* = 11.3 Hz, 1H), 1.51 (s, 3H), 1.34 (s, 3H).

^13^C NMR (126 MHz, CDCl_3_) δ 200.50, 173.47, 172.52, 157.00, 154.94, 154.30, 146.10, 132.11, 129.24, 128.23, 123.12, 122.30, 118.15, 115.91, 114.24, 110.96, 104.00, 93.25, 87.95, 83.00, 76.49, 54.90, 46.75, 46.37, 45.34, 44.70, 43.27, 40.37, 31.53, 31.42, 24.22, 22.73.

HRMS (ESI) *m/z* calculated for C_32_H_33_N2O_7_, [M + H]^+^ 557.2288; Found: 557.2290.

##### Characterization Data of **B10**

^1^H NMR (400 MHz, CDCl_3_) δ 8.17 (s, 1H), 7.57 (d, *J* = 8.9 Hz, 1H), 7.43 (d, *J* = 8.1 Hz, 2H), 7.14 (d, *J* = 8.0 Hz, 2H), 6.93 (s, 1H), 6.49 (d, *J* = 8.9 Hz, 1H), 4.64 (s, 1H), 2.74–2.63 (m, 1H), 2.59 (dd, *J* = 12.2, 3.5 Hz, 1H), 2.54 (d, *J* = 6.1 Hz, 2H), 2.49 (dd, *J* = 15.5, 5.3 Hz, 1H), 2.37 (s, 3H), 2.17 (dd, *J* = 11.6, 2.9 Hz, 2H), 2.10 (d, *J* = 7.8 Hz, 1H), 2.07 (s, 1H), 2.01 (dd, *J* = 11.4, 3.0 Hz, 1H), 1.89 (dd, *J* = 12.4, 7.2 Hz, 1H), 1.73 (d, *J* = 11.4 Hz, 1H), 1.53 (s, 3H), 1.37 (s, 3H).

^13^C NMR (126 MHz, CDCl_3_) δ 200.59, 173.42, 172.49, 156.73, 154.96, 154.23, 138.91, 131.72, 129.08, 128.24, 122.49, 119.39, 114.21, 110.97, 103.95, 93.24, 87.98, 82.92, 76.52, 54.92, 46.74, 46.38, 45.35, 44.71, 43.29, 40.37, 31.49, 31.36, 24.24, 22.74, 22.72.

HRMS (ESI) *m/z* calculated for C_32_H_34_NO_7_, [M + H]^+^ 556.2335; Found: 556.2334.

##### Characterization Data of **B11**

^1^H NMR (500 MHz, Chloroform-*d*) δ 8.19 (s, 1H), 7.58 (d, *J* = 9.0 Hz, 1H), 7.43 (d, *J* = 7.9 Hz, 2H), 7.16 (d, *J* = 7.9 Hz, 2H), 6.91 (s, 1H), 6.49 (d, *J* = 8.9 Hz, 1H), 4.62 (s, 1H), 2.65 (d, *J* = 7.7 Hz, 2H), 2.63 (s, 1H), 2.56 (dd, *J* = 12.0, 4.2 Hz, 1H), 2.54–2.50 (m, 2H), 2.47 (dd, *J* = 12.8, 4.1 Hz, 1H), 2.15 (dd, *J* = 11.7, 3.2 Hz, 1H), 2.08–2.02 (m, 1H), 1.99 (dd, *J* = 11.5, 2.8 Hz, 1H), 1.97–1.92 (m, 1H), 1.88 (td, *J* = 11.7, 10.8, 6.2 Hz, 2H), 1.72 (d, *J* = 11.4 Hz, 1H), 1.51 (s, 3H), 1.35 (s, 3H), 1.23 (t, *J* = 7.6 Hz, 3H).

^13^C NMR (126 MHz, CDCl_3_) δ 200.51, 173.57, 172.93, 156.73, 155.15, 154.37, 145.16, 131.79, 128.34, 127.87, 122.49, 119.61, 114.22, 111.05, 103.88, 93.20, 87.94, 82.96, 76.51, 54.91, 46.78, 46.37, 45.37, 44.70, 43.29, 40.37, 31.58, 31.44, 28.83, 24.21, 22.73, 15.27.

HRMS (ESI) *m/z* calculated for C_34_H_36_NO_7_, [M + H]^+^ 570.2492; Found: 570.2490.

##### Characterization Data of **B12**

^1^H NMR (400 MHz, CDCl_3_) δ 11.84 (s, 1H), 11.19 (s, 1H), 8.16 (s, 1H), 7.57 (d, *J* = 8.9 Hz, 1H), 7.44 (d, *J* = 8.1 Hz, 2H), 7.15 (d, *J* = 8.1 Hz, 2H), 6.93 (s, 1H), 6.49 (d, *J* = 8.9 Hz, 1H), 4.66 (s, 1H), 2.75–2.65 (m, 1H), 2.62 (t, *J* = 7.7 Hz, 2H), 2.58–2.54 (m, 2H), 2.51 (d, *J* = 12.9 Hz, 1H), 2.24–2.14 (m, 2H), 2.09 (d, *J* = 11.5 Hz, 1H), 2.02 (dd, *J* = 11.3, 3.3 Hz, 1H), 1.92 (ddt, *J* = 19.1, 13.0, 6.9 Hz, 2H), 1.74 (d, *J* = 11.4 Hz, 1H), 1.64–1.56 (m, 2H), 1.54 (s, 3H), 1.38 (s, 3H), 1.34 (d, *J* = 7.4 Hz, 1H), 1.32–1.25 (m, 2H), 0.94 (t, *J* = 7.3 Hz, 3H).

^13^C NMR (101 MHz, CDCl_3_) δ 200.64, 173.38, 172.37, 156.69, 154.95, 154.21, 143.93, 131.73, 128.42, 128.20, 122.52, 119.55, 114.23, 110.98, 103.84, 93.37, 88.08, 82.85, 76.55, 54.92, 46.74, 46.39, 45.31, 44.72, 43.27, 40.35, 35.63, 33.35, 31.45, 31.35, 24.28, 22.71, 22.33, 13.96.

HRMS (ESI) *m/z* calculated for C_36_H_40_NO_74_, [M + H]^+^ 598.2805; Found: 598.2803.

##### Characterization Data of **B13**

^1^H NMR (500 MHz, CDCl_3_) δ 8.17 (s, 1H), 7.59 (d, *J* = 8.9 Hz, 1H), 7.47 (d, *J* = 8.6 Hz, 2H), 6.91 (s, 1H), 6.86 (d, *J* = 8.6 Hz, 2H), 6.50 (d, *J* = 8.9 Hz, 1H), 4.64 (s, 1H), 3.83 (s, 3H), 2.68 (td, *J* = 14.2, 4.6 Hz, 1H), 2.63–2.56 (m, 1H), 2.56–2.51 (m, 2H), 2.49 (dd, *J* = 13.1, 3.3 Hz, 1H), 2.17 (dd, *J* = 11.9, 4.1 Hz, 2H), 2.11 (d, *J* = 17.5 Hz, 1H), 2.01 (dd, *J* = 11.4, 3.5 Hz, 1H), 1.95 (dd, *J* = 13.2, 4.9 Hz, 1H), 1.89 (dd, *J* = 12.4, 7.2 Hz, 1H), 1.73 (d, *J* = 11.3 Hz, 1H), 1.53 (s, 3H), 1.37 (s, 3H).

^13^C NMR (126 MHz, CDCl_3_) δ 200.81, 173.45, 172.38, 159.92, 156.43, 154.99, 154.24, 133.33, 128.23, 122.54, 114.51, 114.23, 113.95, 110.99, 103.89, 93.19, 88.03, 82.26, 76.54, 55.31, 46.75, 46.37, 45.33, 44.71, 43.28, 40.36, 31.51, 31.36, 24.28, 22.72.

HRMS (ESI) *m/z* calculated for C_33_H_34_NO_2_, [M + H]^+^ 572.2284; Found: 572.2283.

##### Characterization Data of **B14**

^1^H NMR (500 MHz, CDCl_3_) δ 8.13 (s, 1H), 7.56 (d, *J* = 8.9 Hz, 1H), 7.50 (dd, *J* = 8.6, 5.4 Hz, 2H), 7.03 (t, *J* = 8.7 Hz, 2H), 6.93 (s, 1H), 6.49 (d, *J* = 8.9 Hz, 1H), 4.65 (s, 1H), 2.73–2.64 (m, 1H), 2.56 (s, 2H), 2.53 (d, *J* = 5.5 Hz, 1H), 2.17 (dd, *J* = 11.8, 3.4 Hz, 2H), 2.10 (d, *J* = 13.4 Hz, 1H), 2.06 (s, 1H), 2.02 (dd, *J* = 11.4, 3.0 Hz, 1H), 1.91 (ddd, *J* = 18.2, 12.1, 8.0 Hz, 2H), 1.73 (d, *J* = 11.4 Hz, 1H), 1.53 (s, 3H), 1.36 (s, 3H).

^13^C NMR (126 MHz, CDCl_3_) δ 200.42, 173.35, 172.37, 161.72, 157.06, 154.95, 154.20, 133.77, 133.71, 128.16, 122.28, 118.60, 115.75, 115.57, 114.22, 111.00, 103.84, 91.91, 88.15, 83.29, 76.55, 54.87, 46.75, 46.39, 45.28, 44.71, 43.23, 40.32, 31.33, 31.30, 24.25, 22.67.

HRMS (ESI) *m/z* calculated for C_32_H_31_FNO_7_, [M + H]^+^ 560.2085; Found: 560.2085.

##### Characterization Data of **B15**

^1^H NMR (500 MHz, CDCl_3_) δ 8.12 (s, 1H), 7.56 (d, *J* = 8.9 Hz, 1H), 7.45 (d, *J* = 8.1 Hz, 2H), 7.31 (d, *J* = 8.1 Hz, 2H), 6.95 (s, 1H), 6.49 (d, *J* = 8.9 Hz, 1H), 4.66 (s, 1H), 2.75–2.66 (m, 1H), 2.58 (d, *J* = 11.5 Hz, 2H), 2.54 (s, 1H), 2.22–2.14 (m, 2H), 2.08 (d, *J* = 12.9 Hz, 2H), 2.03 (dd, *J* = 11.6, 3.4 Hz, 1H), 1.97–1.86 (m, 2H), 1.74 (d, *J* = 11.3 Hz, 1H), 1.53 (s, 3H), 1.37 (s, 3H).

^13^C NMR (126 MHz, CDCl_3_) δ 200.33, 173.31, 172.30, 157.29, 154.94, 154.18, 134.77, 133.00, 128.69, 128.15, 122.23, 120.99, 114.22, 111.01, 103.84, 91.84, 88.18, 84.50, 76.56, 60.47, 54.88, 46.75, 46.42, 45.27, 44.72, 43.23, 40.32, 31.29, 31.26, 24.24, 22.66.

HRMS (ESI) *m/z* calculated for C_32_H_31_ClNO_7_, [M + H]^+^ 576.1789; Found: 576.1788.

##### Characterization Data of **C1**

^1^H NMR (500 MHz, CDCl_3_) δ 7.92 (s, 1H), 7.65 (d, *J* = 8.9 Hz, 1H), 7.31 (d, *J* = 7.5 Hz, 2H), 7.25 (t, *J* = 7.4 Hz, 2H), 7.19 (t, *J* = 7.3 Hz, 1H), 6.53 (d, *J* = 8.8 Hz, 1H), 4.65–4.55 (m, 1H), 4.13 (q, *J* = 7.1 Hz, 1H), 3.96–3.88 (m, 2H), 3.65 (d, *J* = 14.7 Hz, 1H), 2.54–2.48 (m, 1H), 2.32 (s, 3H), 2.16 (s, 1H), 2.02 (s, 3H), 1.92–1.84 (m, 2H), 1.80–1.76 (m, 1H), 1.75–1.71 (m, 1H), 1.70–1.67 (m, 1H), 1.64 (d, *J* = 17.8 Hz, 2H), 1.49 (s, 3H), 1.35 (s, 3H).

^13^C NMR (126 MHz, CDCl_3_) δ 205.24, 201.90, 173.16, 172.27, 155.28, 155.00, 154.12, 152.12, 136.95, 128.71, 128.62, 128.38, 126.85, 114.15, 113.70, 111.31, 111.15, 104.05, 88.75, 76.93, 50.47, 49.53, 49.07, 44.87, 44.07, 43.48, 40.32, 35.93, 33.94, 32.23, 29.21, 22.94, 22.85, 17.90, 14.19.

HRMS (ESI) *m/z* calculated for C_37_H_40_NO_9_, [M + H]^+^ 642.2703; Found: 642.2703.

##### Characterization Data of **C2**

^1^H NMR (500 MHz, CDCl_3_) δ 11.86 (s, 1H), 11.01 (s, 1H), 7.93 (s, 1H), 7.66 (d, *J* = 9.0 Hz, 1H), 7.21 (d, *J* = 7.9 Hz, 2H), 7.04 (d, *J* = 7.8 Hz, 2H), 6.54 (d, *J* = 8.9 Hz, 1H), 4.66 (s, 1H), 3.93 (s, 1H), 3.85 (d, *J* = 14.6 Hz, 1H), 3.62 (d, *J* = 14.5 Hz, 1H), 2.62–2.55 (m, 1H), 2.41 (ddd, *J* = 14.4, 12.6, 4.5 Hz, 2H), 2.31 (s, 3H), 2.23 (s, 1H), 2.15 (s, 3H), 2.13 (d, *J* = 4.6 Hz, 1H), 2.02 (s, 3H), 1.93–1.87 (m, 2H), 1.79 (ddd, *J* = 11.7, 6.8, 3.2 Hz, 2H), 1.72 (d, *J* = 4.4 Hz, 1H), 1.64 (dd, *J* = 21.0, 6.6 Hz, 2H), 1.50 (s, 3H), 1.37 (s, 3H).

^13^C NMR (126 MHz, CDCl_3_) δ 205.34, 201.74, 173.06, 172.36, 155.22, 155.07, 154.04, 152.23, 136.38, 133.69, 129.24, 128.57, 128.35, 114.16, 113.59, 111.30, 111.21, 103.93, 88.74, 76.94, 50.47, 49.53, 49.20, 45.06, 44.08, 43.53, 40.33, 35.42, 33.97, 32.50, 30.53, 29.17, 22.96, 22.83, 20.77, 17.90.

HRMS (ESI) *m/z* calculated for C_38_H_42_NO_9_, [M + H]^+^ 656.2860; Found: 656.2859.

##### Characterization Data of **C3**

^1^H NMR (500 MHz, CDCl_3_) δ 7.97 (s, 1H), 7.65 (d, *J* = 8.9 Hz, 1H), 7.24 (d, *J* = 7.8 Hz, 2H), 7.08 (d, *J* = 7.8 Hz, 2H), 6.53 (d, *J* = 8.9 Hz, 1H), 4.65 (t, *J* = 3.1 Hz, 1H), 4.14 (q, *J* = 7.1 Hz, 1H), 3.93 (s, 1H), 3.80 (d, *J* = 14.5 Hz, 1H), 3.75 (q, *J* = 7.0 Hz, 1H), 3.64 (d, *J* = 14.5 Hz, 1H), 2.61–2.54 (m, 1H), 2.49–2.45 (m, 2H), 2.40 (s, 1H), 2.31 (s, 3H), 2.27–2.23 (m, 2H), 2.15–2.11 (m, 1H), 2.01 (s, 3H), 1.90 (d, *J* = 4.2 Hz, 1H), 1.82–1.75 (m, 2H), 1.71 (s, 2H), 1.49 (s, 3H), 1.37 (s, 3H), 1.09 (t, *J* = 7.6 Hz, 3H).

^13^C NMR (126 MHz, CDCl_3_) δ 205.47, 201.76, 173.11, 172.43, 155.07, 154.09, 152.27, 142.77, 133.82, 128.64, 128.35, 128.02, 117.08, 114.16, 113.57, 111.37, 111.19, 103.92, 88.62, 76.90, 74.75, 50.43, 49.52, 49.21, 45.08, 44.11, 43.53, 40.35, 35.44, 33.98, 32.62, 30.68, 29.18, 28.28, 22.99, 22.82, 17.91, 15.38.

HRMS (ESI) *m/z* calculated for C_39_H_44_NO_9_, [M + H]^+^ 670.3016; Found: 670.3018.

##### Characterization Data of **C4**

^1^H NMR (500 MHz, CDCl_3_) δ 11.86 (s, 1H), 11.02 (s, 1H), 7.98 (s, 1H), 7.65 (d, *J* = 8.9 Hz, 1H), 7.23 (d, *J* = 7.9 Hz, 2H), 7.06 (d, *J* = 8.0 Hz, 2H), 6.53 (d, *J* = 8.9 Hz, 1H), 4.64 (s, 1H), 3.92 (s, 1H), 3.80 (d, *J* = 14.5 Hz, 1H), 3.64 (d, *J* = 14.5 Hz, 1H), 2.61–2.55 (m, 1H), 2.54 (d, *J* = 2.7 Hz, 1H), 2.44 (d, *J* = 7.5 Hz, 2H), 2.43–2.37 (m, 2H), 2.31 (s, 3H), 2.29 (d, *J* = 13.3 Hz, 1H), 2.24 (s, 1H), 2.13 (d, *J* = 6.6 Hz, 1H), 2.01 (s, 3H), 1.90 (d, *J* = 11.6 Hz, 2H), 1.87 (d, *J* = 3.6 Hz, 1H), 1.80–1.71 (m, 2H), 1.65 (ddd, *J* = 22.3, 12.4, 2.9 Hz, 2H), 1.49 (s, 3H), 1.44 (dt, *J* = 7.2, 2.0 Hz, 2H), 1.36 (s, 3H), 1.26–1.20 (m, 2H), 0.83 (t, *J* = 7.3 Hz, 3H).

^13^C NMR (126 MHz, CDCl_3_) δ 205.51, 201.80, 173.09, 172.50, 155.09, 154.10, 152.25, 141.49, 133.81, 132.17, 132.09, 128.70, 128.56, 128.36, 114.16, 113.59, 111.36, 111.19, 103.93, 88.55, 76.87, 50.43, 49.52, 49.21, 45.06, 44.12, 43.51, 40.36, 35.45, 35.11, 33.98, 33.51, 32.67, 30.70, 29.18, 23.02, 22.81, 22.40, 17.92, 13.86.

HRMS (ESI) *m/z* calculated for C_41_H_48_NO_9_, [M + H]^+^ 698.3329; Found: 698.3328.

##### Characterization Data of **C5**

^1^H NMR (500 MHz, CDCl_3_) δ 11.85 (s, 1H), 11.00 (s, 1H), 7.99 (s, 1H), 7.64 (d, *J* = 9.0 Hz, 1H), 7.23 (td, *J* = 7.9, 5.9 Hz, 1H), 7.17–7.04 (m, 2H), 6.92 (td, *J* = 8.5, 2.6 Hz, 1H), 6.53 (d, *J* = 8.9 Hz, 1H), 4.64 (s, 1H), 3.95 (s, 1H), 3.75 (s, 2H), 2.61 (td, *J* = 13.4, 3.5 Hz, 1H), 2.50 (ddd, *J* = 14.8, 12.6, 4.3 Hz, 1H), 2.32 (s, 3H), 2.22 (s, 1H), 2.12 (q, *J* = 5.5 Hz, 1H), 2.01 (s, 3H), 1.99–1.95 (m, 1H), 1.94–1.87 (m, 2H), 1.81–1.77 (m, 1H), 1.73 (dd, *J* = 13.3, 4.2 Hz, 2H), 1.65 (d, *J* = 6.9 Hz, 2H), 1.50 (s, 3H), 1.38 (s, 3H).

^13^C NMR (126 MHz, CDCl_3_) δ 205.11, 201.70, 172.96, 172.25, 163.82, 161.87, 154.98, 154.29, 154.01, 151.98, 139.17, 129.90, 128.38, 124.50, 115.94, 114.15, 113.70, 111.66, 111.14, 104.03, 88.83, 76.92, 50.39, 49.47, 49.14, 45.02, 44.07, 43.49, 40.32, 35.68, 33.95, 32.33, 30.39, 29.22, 22.90, 22.85, 17.84.

HRMS (ESI) *m/z* calculated for C_37_H_39_FNO_9_, [M + H]^+^ 660.2609; Found: 660.2608.

##### Characterization Data of **C6**

^1^H NMR (500 MHz, CDCl_3_) δ 11.78 (s, 1H), 10.99 (s, 1H), 8.01 (s, 1H), 7.61 (d, *J* = 8.9 Hz, 1H), 7.32 (dd, *J* = 8.5, 5.5 Hz, 2H), 6.93 (t, *J* = 8.7 Hz, 2H), 6.51 (d, *J* = 8.9 Hz, 1H), 4.64 (s, 1H), 3.92 (s, 1H), 3.72 (d, *J* = 14.2 Hz, 1H), 3.64 (d, *J* = 14.3 Hz, 1H), 2.64–2.52 (m, 2H), 2.30 (s, 3H), 2.26 (s, 1H), 2.14–2.08 (m, 1H), 2.06–2.03 (m, 2H), 1.99 (s, 3H), 1.94–1.85 (m, 2H), 1.76 (td, *J* = 13.1, 12.6, 3.2 Hz, 2H), 1.72–1.58 (m, 2H), 1.48 (s, 3H), 1.37 (s, 3H).

^13^C NMR (126 MHz, CDCl_3_) δ 205.41, 201.72, 172.97, 172.32, 162.74, 160.79, 154.95, 154.48, 154.07, 152.09, 132.35, 130.33, 128.32, 115.39, 115.22, 114.15, 113.62, 111.47, 111.08, 103.97, 88.76, 76.93, 50.35, 49.44, 49.18, 45.04, 44.06, 43.51, 40.30, 35.04, 33.94, 32.34, 30.56, 29.20, 22.92, 22.84, 17.85.

HRMS (ESI) *m/z* calculated for C_37_H_39_FNO_9_, [M + H]^+^ 660.2609; Found: 660.2611.

##### Characterization Data of **C7**

^1^H NMR (500 MHz, DMSO-*d*_6_) δ 7.48 (d, *J* = 8.5 Hz, 1H), 7.44–7.35 (m, 4H), 6.17 (d, *J* = 8.6 Hz, 1H), 4.42 (s, 1H), 3.91 (s, 1H), 3.76 (d, *J* = 14.4 Hz, 1H), 3.49 (d, *J* = 14.5 Hz, 1H), 2.33 (d, *J* = 13.5 Hz, 1H), 2.29 (s, 3H), 2.21–2.12 (m, 3H), 2.07 (td, *J* = 13.9, 13.0, 4.3 Hz, 1H), 1.88 (s, 3H), 1.85 (s, 1H), 1.81 (dd, *J* = 11.9, 3.1 Hz, 1H), 1.77–1.68 (m, 1H), 1.50 (ddd, *J* = 25.7, 11.3, 5.1 Hz, 2H), 1.30 (s, 3H), 1.28 (s, 3H).

^13^C NMR (126 MHz, DMSO-*d*_6_) δ 206.58, 202.30, 173.09, 172.33, 152.34, 151.09, 143.30, 136.49, 131.77, 131.13, 128.81, 124.16, 119.14, 114.10, 112.74, 110.85, 110.71, 105.86, 86.69, 76.00, 50.23, 49.75, 49.16, 45.18, 44.02, 43.57, 40.35, 35.06, 33.51, 33.31, 30.49, 29.53, 23.91, 22.99, 17.85.

HRMS (ESI) *m/z* calculated for C_37_H_39_ClNO_9_, [M + H]^+^ 676.2313; Found: 676.2313.

### 3.3. DFT Calculations on Transition States

The DFT (density function theory) calculations were carried out in the Gaussian 09 software package (revision D.01) [31]. The geometric structures of intermediates and transition states were fully optimized using the M06-2X functional method combined with the 6-31G + (d) basis set. Solvent effects were considered implicitly during optimization and energy evaluation through the CPCM polarizable continuum model as implemented in Gaussian 09 [32,33]. The solvent DMSO was used. Single-point energies were also calculated using M06-2X, and the 6-31G + (d) basis set. The frequency analysis was performed based on the optimized structures to verify the energy minimum and transition states.

All discussed energy values are the Gibbs free energies calculated at the temperature 298 K, and the distances are in angstrom. Gibbs free energies (ΔG) were used for the discussion on the relative stabilities of the considered structures. The theoretical ratio of reaction products was obtained through the energy of the different transition states using a Maxwell−Boltzmann distribution at 298 K at which thermal and entropic corrections to energy were calculated [34]. Computed structures were illustrated in GaussView.

### 3.4. The MIC Determination

All of the tested compounds were prepared to different concentrations with methanol, and 150 μL of the respective methanol solution was used to mix with 15 mL LB (Luria–Bertani) medium with 1% agar in each Petri dish. Linezolid and PTM were used as reference compounds for comparison. In vitro bioassay for compounds was carried out using clinical strains of *S. aureus*, *E. coli*, and *Klebsilla pneumoniae*. The bacteria were cultivated in LB medium and incubated for 16 h at 37 °C, 250 rpm. The resulting bacteria were diluted to approximate 0.25 at OD_600_, which were further diluted 10_4_-fold. The diluted bacteria (2 μL) were added to Petri dish containing the respective compounds. The resulting Petri dishes were incubated for 16 h at 37 °C. The determination of MIC is the concentration of Petri dishes with no visible growth of bacteria.

### 3.5. Molecular Docking

The molecular modeling studies in this work were carried out on an Intel core i5 2.5 GHz processor, 8 GB memory with Windows 10 operating system using Molecular Operating Environment (MOE 2010.06; Chemical Computing Group, Montreal, QC, Canada) as the computational platform. All the energy minimizations were performed with MOE until a RMSD gradient of 0.05 Kcal·mol^−1^·Å^−1^ with MMFF94X force-field, and the partial charges were automatically calculated. The X-ray crystallographic structure of ecFabF complexed with PTM (PDB ID: 2GFX) was obtained from the protein data bank. The enzyme was prepared for docking studies where: (i) errors in crystallographic structure were corrected with prepare structure step and waters were removed from the complex. (ii) Hydrogen atoms and partial charges were added with protonate 3D step. (iii) The active site was defined by using PTM as template. (iv) The generated model was then used in predicting the binding modes of synthesized analogues.

### 3.6. Molecular Dynamics Simulation

The molecular dynamics simulation was performed on dual Intel Xeon E5-2678V3 2.5 GHz processor, 32 GB memory, with Ubuntu 16.04 operating system using NAMD (Linux-x86_64-multicore-CUDA edition) as the computational platform. All the calculations were accelerated with CUDA 9.1 on dual NVIDIA TITAN Xp graphics card. The Binding stabilities of the selected ligand–FabF complex were simulated with CHARMM general force field (protein: https://www.charmm.org/charmm/, ligand: https://cgenff.umaryland.edu/) (accessed on 1 October 2021). The simulation was performed with implicit solvent model containing 0.15 M sodium chloride. An equilibration simulation from 60 K to 300 K for 1.242 ns was processed before taking 50 ns production simulation at 300 K. The results were saved per 2 fs. The VMD 1.9.3 was used to analyze the generated MD trajectory file and calculate the root mean-square deviation (RMSD) of the protein backbone and the nonbond interaction energy between the ligand and protein. In RMSD analysis, the actual structural fluctuations were measured by comparing each frame with frame 0.

### 3.7. Metabolic Stability Assay

Each compound was incubated in duplicate with pooled human liver microsomes at 37 °C. The tested compounds were pre-incubated with pooled human liver microsomes in phosphate buffer (pH 7.4) for 5 min in a shaking water bath at 37 °C. The reaction was initiated by adding a NADPH-generating system and incubated for 0, 15, 30, 45, and 60 min. The reaction was stopped by transferring the incubation mixture to CH_3_CN/MeOH. Samples were then mixed and centrifuged. Supernatants were used for HPLC–MS/MS analysis. Data were calculated as percent parent compounds remaining by assuming zero-minute time point peak area ratio (analyte/IS) as 100% and dividing remaining time point peak area ratios by the zero-minute time point peak area ratio. Data were subjected to fit a first-order decay model to calculate both the slope and half-life.

Equilibrium dialysis was used to determine the plasma protein binding. All of the test compounds were treated in triplicate by RED Rapid Equilibrium Dialysis Device (Thermo Scientific, Waltham, MA, USA). In brief, the initial compound concentration (5 μM) in the plasma chamber was first set, followed by the addition of phosphate buffered saline (PBS) to the receiver chamber. The Equilibrium Dialysis Device was allowed to shake for 6 h in a 37 °C incubator. Then, 25 μL solution was sampled from the plasma and PBS chambers, respectively, and they were diluted with either blank PBS or plasma to achieve a 1:1 ratio of plasma: PBS for all samples. The concentrations of the tested compounds in the plasma and PBS chambers were determined by LC–MS/MS. The fraction bound was calculated as ([plasma]—[PBS])/[plasma].

### 3.8. Animal Protocols

All animal protocols were approved by the Animal Care and Use Committee of Central South University. All experimental procedures complied with the Guide for the Care and Use of Laboratory Animals (1996). 

### 3.9. Mouse Peritonitis Model 

Six to eight-week-old female C57BL/6J mice (18–21 g) were intraperitoneally injected with 2 × 10^7^ CFU of the overnight MRSA inoculum in 0.5 mL physiological saline containing 5% (w/v) mucin to generate the lethal peritonitis mice model. After 1 h and 5 h post-infection, the mice were divided into individual groups (n = 5) and injected with 0.2 mL/mouse of **4** (10 mg/kg), **A4** (5, 10, 20, 30 or 50 mg/kg), **B8** (10 mg/kg), PTM (10 mg/kg), vancomycin (50 mg/kg), and saline. Compound **A4** (5, 10, 20, 30, or 50 mg/kg) was formulated in DMSO (1.25%, 2.5%, 5.0%, 7.5%, or 12.5%), 30% propanediol, 30% polyethylene glycol 400, and physiological saline to 0.5, 1, 2, 3, or 5 mg/mL for injection. Compounds **4** (10 mg/kg), **B8** (10 mg/kg), and PTM (10 mg/kg) were formulated with 2.5% DMSO, 30% propanediol, 30% polyethylene glycol 400, and 37.5% physiological saline to 1 mg/mL for injection. Vancomycin was dissolved in physiological saline to a final concentration of 5 mg/mL. The ability of the tested compounds to rescue mice from lethal peritonitis was observed twice daily, and mice survival was tracked for 7 days.

### 3.10. Mouse Skin Infection with MRSA

Mouse MRSA skin infection study was performed as described before [14]. Female BABL/c mice (20–22 g, 7-weeks old) were anesthetized by intraperitoneal injection of 4% chloral hydrate (200 μL), and the hair of mice back surface was removed and washed with povidone iodine–propanol solution. A gauze (15 × 15 mm) was preheated in boiling water (90 °C) for 10 min, and then placed on the back of each mouse for 40 s to generate the burns. Next, the burn was bound with sterile gauze and infected with 1 × 10^8^ colony-forming units (CFU) of MRSA. An open wound was witnessed at the burns after 48 h post infection. The infected mice were then divided into three groups (5 per group). **A4** (200 μg) was formulated into 2% ointment consisting of 1.4% carbomer, 1.4% triethanolamine, 14% propanediol, 14% glycerinum, and 1% azone. Mupirocin (2% *w/w*) were used as the positive control, while vehicle used as negative control. All mice were treated twice a day for 7 days. Mice were euthanized after 24 h of the last treatment, and the wound area was slightly swabbed with 70% ethanol. Wound area (1 cm^2^) was excised, homogenized, and plated onto TSB agar plates to count viable bacteria. Other skin biopsy specimens removed from the wounds were stored at 4% paraformaldehyde solution to be used for hematoxylin and eosin (HE) staining.

## 4. Conclusions

Natural products have had transformative impacts on human medicine in history [35,36]. PTM is a new class of natural antibiotics that operates by inhibiting FASII and shows no cross-resistance to many other antibiotics [6,30,37]. Ideally, more diverse PTM derivatives may be prepared and biologically evaluated in order to discover PTM-based drug leads with improved in vivo efficacy. In this work, thirty-two PTM derivatives were synthesized via Heck, Sonogashira, and one-pot Sonogashira/cycloaddition cascade reactions in a highly efficient way. PTM derivatives C1–C7 containing substituted 4*H*-pyrans were obtained in a stereochemically controlled fashion due to the presence of the rigid tetracyclic ring in the PTM scaffold [13,24]. About half of the synthesized compounds were equipotent to PTM against the tested clinical S. aureus isolates. Importantly, the antibacterial activity of compound **A4** was comparable to the clinical drug mupirocin when evaluated in an MRSA-induced mice skin infection model, while **A4** showed improved in vivo efficacy over PTM in a systematic mouse peritonitis model. Finally, we observed certain toxicity of **A4** in mice when given in dosages of 30 or 50 mg/kg by IP injection, which suggests that further toxicological study of these PTM derivatives is needed. Therefore, the approach reported in our study could be adapted to make a library of PTM derivatives from which safer and more effective PTM derivatives may be obtained.

## Figures and Tables

**Scheme 2:**
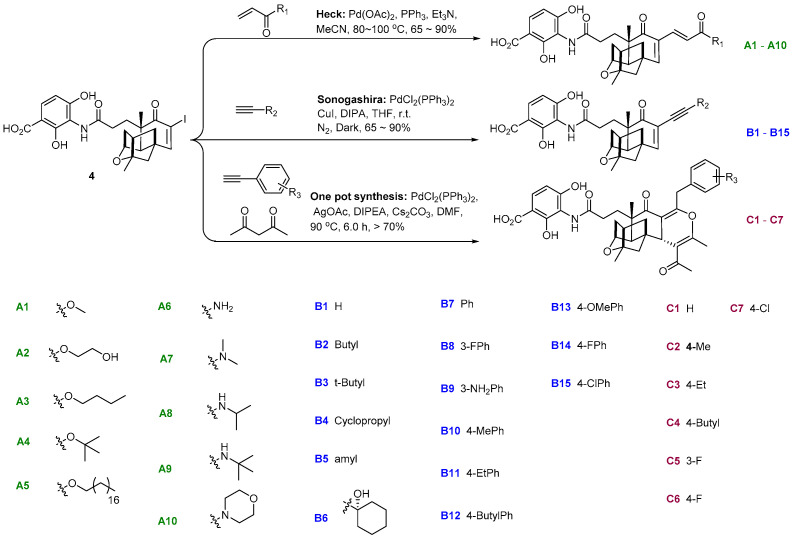
Synthesis of PTM derivatives. Compounds **A1**–**A10**, **B1**–**B15** were prepared in classical Heck and Sonogashira reaction conditions. **C1**–**C7** were prepared via a one-pot synthesis strategy from **4**.

**Table 1:** The antibacterial activities of **4**, **A1**–**A10**, **B1**–**B15,** and **C1**–**C7** against *S. aureus* ATCC 29213, and clinical MSSA and MRSA strains. The reported MICs are based on at least two replicates.

Compounds	ATCC	MSSA		MRSA	
	29213	1	2	3	4
PTM	1	1	1	1	1
Linezolid	1	1	1	1	1
**4**	1	1	1	1	1
**A1**	2	2	2	2	2
**A2**	16	16	16	16	16
**A3**	1	1	1	1	1
**A4**	1	1	1	1	1
**A5**	>64	>64	>64	>64	>64
**A6**	64	64	64	64	64
**A7**	16	16	16	16	16
**A8**	32	32	32	32	32
**A9**	8	8	8	8	8
**A10**	16	16	16	16	16
**B1**	8	8	8	8	8
**B2**	1	1	1	1	1
**B3**	1	1	1	1	1
**B4**	1	1	1	1	1
**B5**	1	1	1	1	1
**B6**	4	4	4	4	4
**B7**	1	1	1	1	1
**B8**	1	1	1	1	1
**B9**	1	1	1	1	1
**B10**	8	4	4	8	4
**B11**	1	1	1	1	1
**B12**	8	8	8	8	8
**B13**	1	1	1	1	1
**B14**	1	1	1	1	1
**B15**	1	1	1	1	1
**C1**	16	16	16	16	16
**C2**	16	16	16	16	16
**C3**	32	32	32	32	32
**C4**	32	32	32	32	32
**C5**	16	16	16	16	16
**C6**	32	32	32	32	32
**C7**	32	32	32	32	32
MIC color scale (μg/mL)		1	2	4	8
		16	32	64	>64

**Table 2:** Human hepatic microsome stability assay and plasma protein binding assay of representative PTM derivatives.

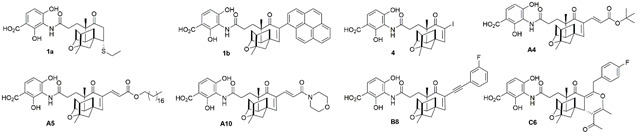			
Compounds	Half-Life in Human Hepatic Microsomes (Min)	Plasma Protein Binding	
		Free %	Bound %
**PTM** ^a^	51	34	66
**1a** ^a^	22	65	35
**1b** ^b^	73	0.1	99.9
**4**	58	13	87
**A4**	26	1.7	98.3
**A5**	76	0.8	99.2
**A10**	>120	23	77
**B8**	96	0.6	99.4
**C6**	17	0.4	99.6

^a^ previously reported [14]. ^b^ previously synthesized [15].

## Data Availability

Data are available upon request.

## Supplementary Materials

The following supporting information can be downloaded at: https://www.mdpi.com/article/10.3390/antibiotics11040425/s1. Table S1: Summary of ^1^H NMR (500 MHz) and ^13^C NMR (126 MHz) data for compound **C2** in CDCl_3_. Figure S1: ^1^H NMR and ^13^C NMR spectra of **2**. Figure S2: ^1^H NMR and ^13^C NMR spectra of **4**. Figures S3–S12: ^1^H NMR and ^13^C NMR spectra of **A1**–**A10**. Figure S13–S27: ^1^H NMR and ^13^C NMR spectra of **B1**–**B15**. Figure S28: ^1^H NMR and ^13^C NMR spectra of **C1**. Figure S29–S33: ^1^H, ^13^C, and 2D NMR spectra of **C2**. Figures S34–S38: ^1^H NMR and ^13^C NMR spectra of **C3**–**C7**. Figure S39: HRMS spectra of **2**. Figure S40: HRMS spectra of **4**. Figure S41: HRMS spectra of **A1**–**A10**. Figure S42: HRMS spectra of **B1**–**B15**. Figure S43: HRMS spectra of **C1**–**C7**. Figure S44: Analysis of the stability of **A4** in LB agar.
