# Supplementary material for: Morphing Natural Product Platensimycin via Heck, Sonogashira, and One-Pot Sonogashira/Cycloaddition Reactions to Produce Antibiotics with In Vivo Activity
Source: Antibiotics (Basel). 2022 Mar 23;11(4):425. doi: 10.3390/antibiotics11040425 (PMC9027111; doi:10.3390/antibiotics11040425)
Supplement: Supplementary file 1 [file antibiotics-11-00425-s001.zip › antibiotics-1627640-supplementary.pdf]

Supporting Information

Morphing Natural Product Platensimycin via Heck, Sonogashira, and One-Pot Sonogashira/Cycloaddition Reactions to Produce Antibiotics with In Vivo Activity

Youchao Deng [1], Yuling Li [1], Zhongqing Wen [1], Claudia H. Ruiz [2], Xiang Weng [1], Michael D. Cameron [2], Yanwen Duan [1,3,4,*], and Yong Huang[1,4*]

1 Xiangya International Academy of Translational Medicine, Central South University, Changsha 410013, China; dengyouchao1206@163.com (Y.D.); lyl1614787460@163.com (Y.L.); wen17375898093@163.com (Z.W.); 202120919@mail.sdu.edu.cn (X.W.)

2 Departments of Molecular Medicine, The Scripps Research Institute, Jupiter, FL 33458, USA; cruiz@scripps.edu (C.H.R.); cameron@scripps.edu (M.D.C.)

3 Hunan Engineering Research Center of Combinatorial Biosynthesis and Natural Product Drug Discovery, Changsha 410011, China

4 National Engineering Research Center of Combinatorial Biosynthesis for Drug Discovery, Changsha 410011, China

* Correspondence: ywduan66@csu.edu.cn (Y.D.); jonghuang@csu.edu.cn (Y.H.)

Table of Contents

Table S1. Summary of ^{1}H NMR (500 MHz) and ^{13}C NMR (126 MHz) data for compound **C2** in $CDCl_3$ 3
Figure S1. ^{1}H NMR and ^{13}C NMR spectra of **2** 5
Figure S2. ^{1}H NMR and ^{13}C NMR spectra of **4** 6
Figures S3-S12. ^{1}H NMR and ^{13}C NMR spectra of **A1 - A10** 7
Figure S13-S27. ^{1}H NMR and ^{13}C NMR spectra of **B1 - B15** 17
Figures S28. ^{1}H NMR and ^{13}C NMR spectra of **C1** 32
Figure S29-S33. ^{1}H, ^{13}C , and 2D NMR spectra of **C2** 33
Figures S34-S38. ^{1}H NMR and ^{13}C NMR spectra of **C3 - C7** 36
Figure S39. HRMS spectra of **2** 41
Figure S40. HRMS spectra of **4** 41
Figure S41. HRMS spectra of **A1 - A10** 42
Figure S42. HRMS spectra of **B1 - B15** 45
Figure S43. HRMS spectra of **C1 - C7** 49
Figure S44. Analysis of the stability of **A4** in LB agar 51
Computational data 52

Table S1. Summary of ^{1}H NMR (500 MHz) and ^{13}C NMR (126 MHz) data of **C2** in $CDCl_3$ (δ in ppm, J in Hz). Assignments are based on 1D and 2D NMR experiments. Proton at C-10 has strong NOESY interactions with protons at both C-18 and C-21 positions, which supports the proposed absolute configuration of **C2**.

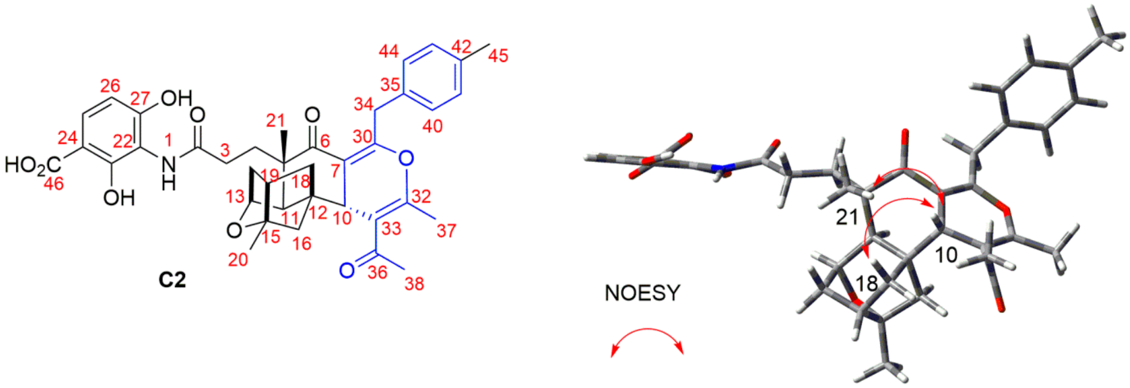

Carbon Number	δ (Carbon)	δ (Proton)
2	173.06	
3	30.53	2.39 – 2.35 (m, 1H), 1.83 – 1.79 (m, 1H),
4	32.5	1.72 – 1.68 (m, 1H), 2.61 – 2.55 (m, 1H),
5	50.47	
6	205.34	
7	111.3	
10	33.97	3.93 (s, 1H),
11	45.06	2.23 (s, 1H),
12	49.2	
13	76.9	4.69 – 4.61 (m, 1H),
15	88.74	
16	49.53	1.52 (d, J = 4.5 Hz, 1H), 1.78 (t, J = 2.7 Hz, 1H),
17	40.33	2.13 – 2.11 (m, 1H), 1.91 (d, J = 11.7 Hz, 1H),
18	44.08	1.66 (d, J = 5.5 Hz, 1H), 1.89 – 1.84 (m, 1H),
19	43.53	2.34 (s, 1H),
20	22.83	1.50 (s, 3H),
21	22.96	1.37 (s, 3H).
22	113.59	
23	154.04	
24	103.93	
25	128.35	7.66 (d, J = 9.0 Hz, 1H),
26	111.21	6.54 (d, J = 8.9 Hz, 1H),
27	155.07	
30	155.22	
32	152.23	
33	114.16	

34	35.42	3.85 (d, *J* = 14.6 Hz, 1H),<br>3.62 (d, *J* = 14.5 Hz, 1H),
35	133.69	
36	201.74	
37	17.9	2.02 (s, 3H),
38	29.17	2.31 (s, 3H),
40	128.57	7.21 (d, *J* = 8.0 Hz, 2H),
41, 43	129.24	7.04 (d, *J* = 7.8 Hz, 2H),
42, 44	136.38	
45	20.77	2.15 (s, 3H),
46	172.36	

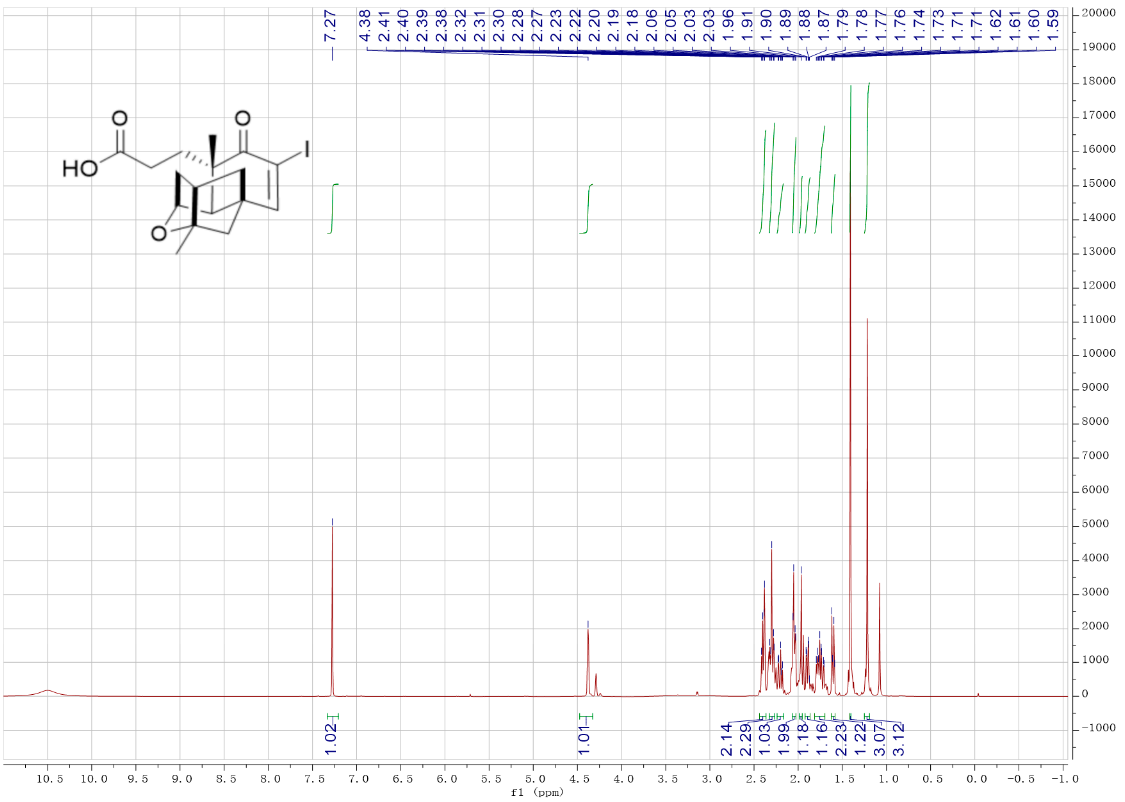

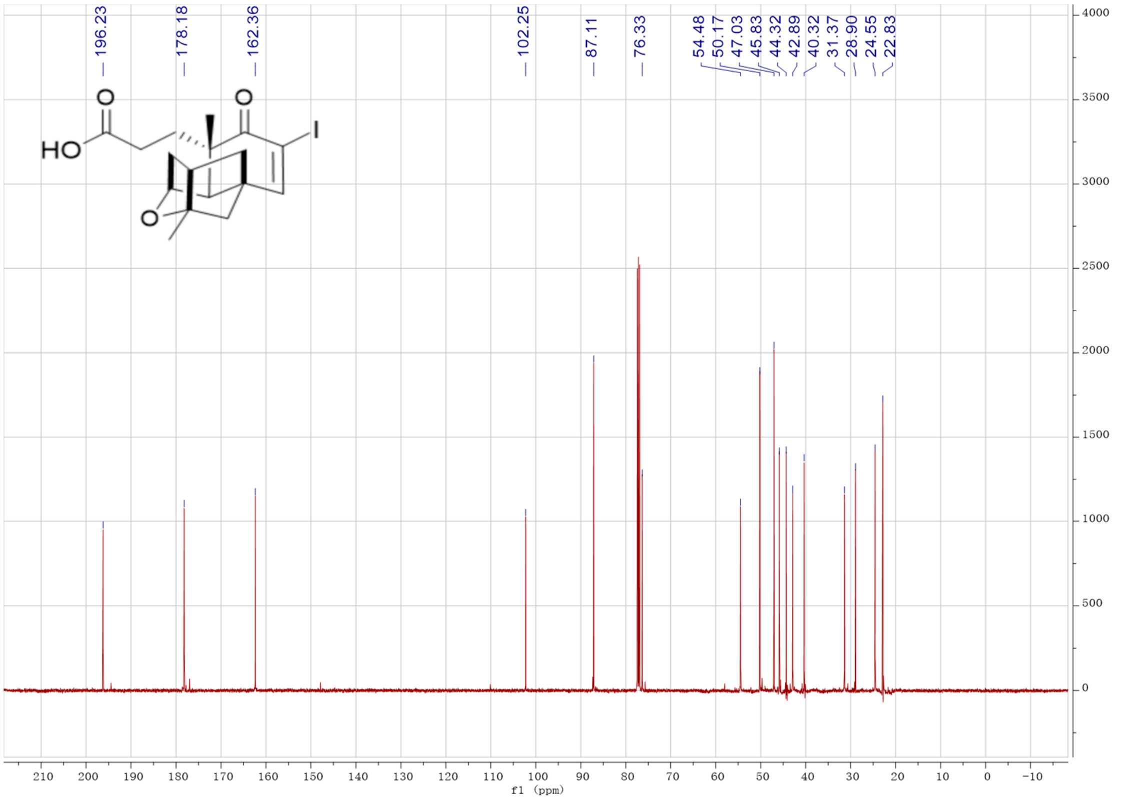

Figure S1. ^{1}H NMR (500 MHz) and ^{13}C NMR (126 MHz) spectra of **2** in $CDCl_3$.

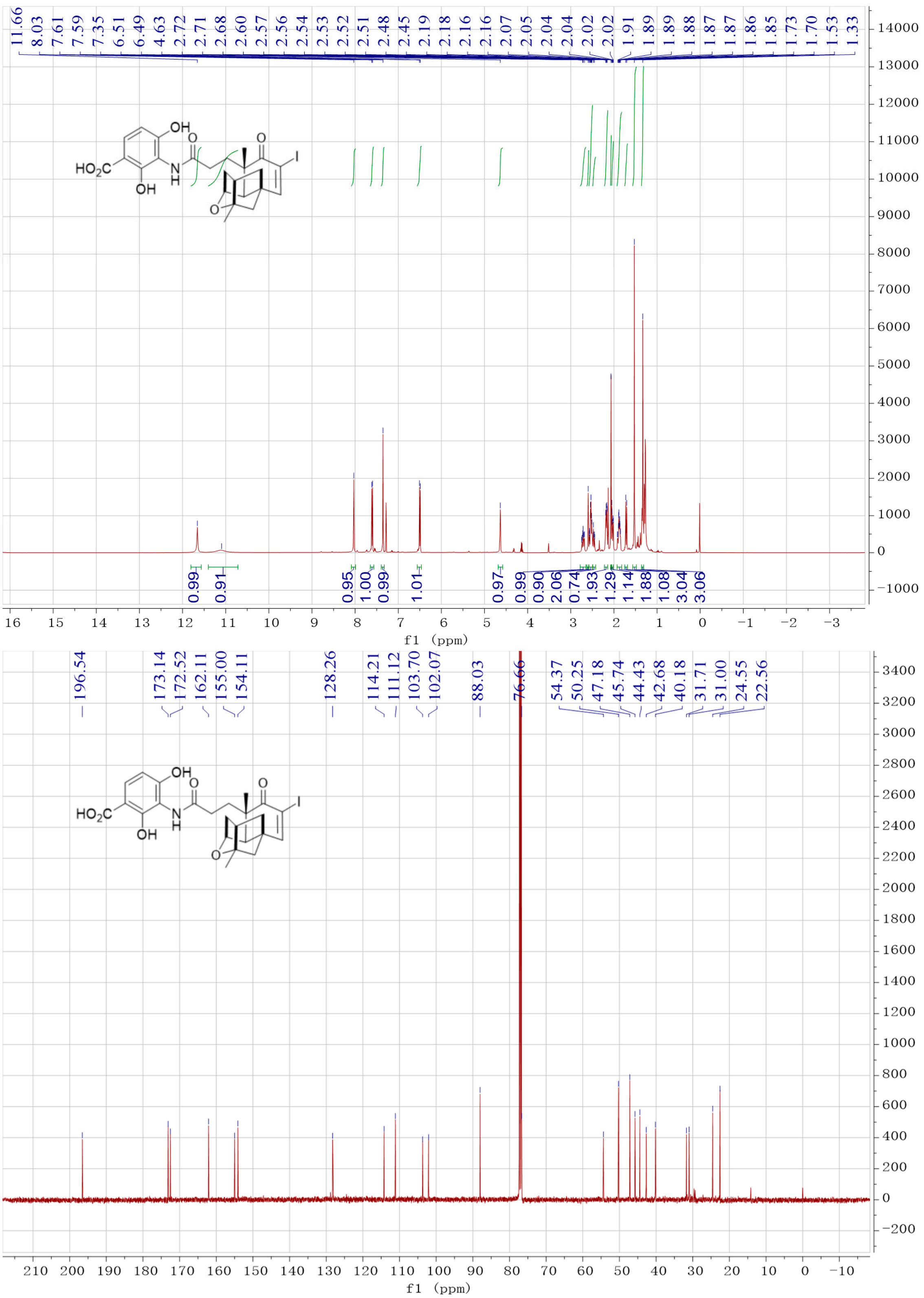

Figure S2. ^{1}H NMR (500 MHz) and ^{13}C NMR (126 MHz) spectra of **4** in $CDCl_3$.

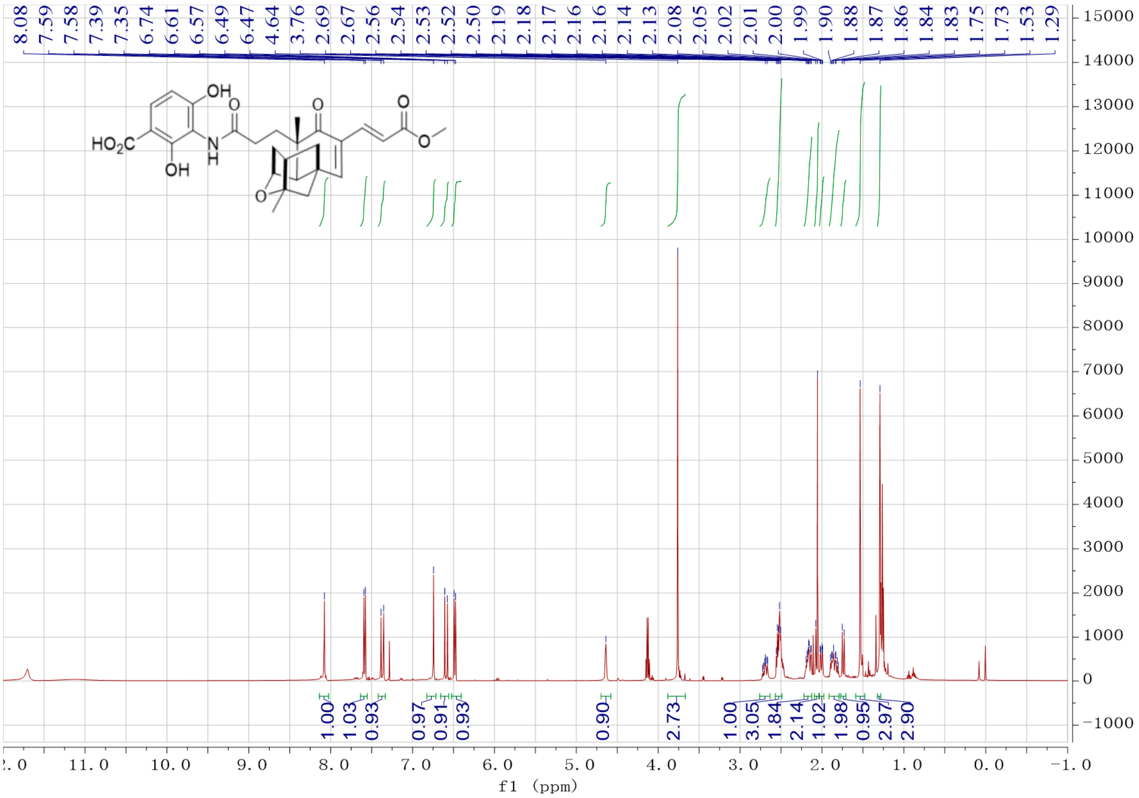

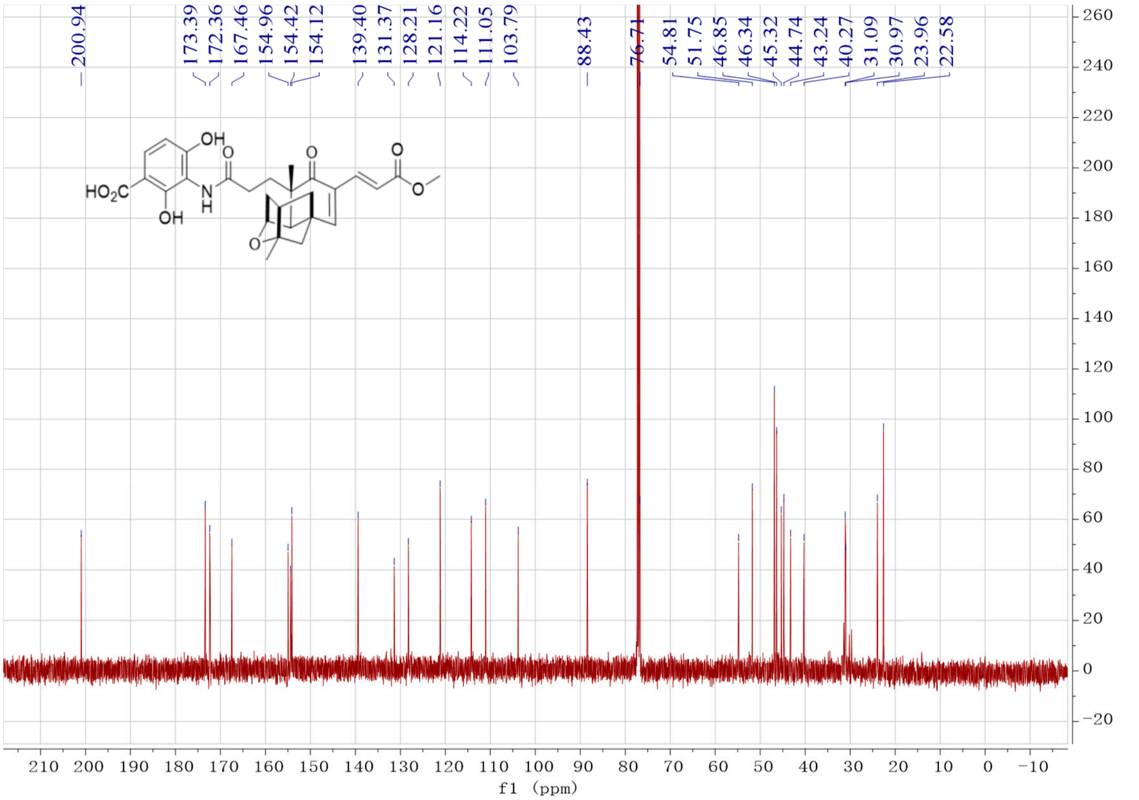

Figure S3. ^{1}H NMR (500 MHz) and ^{13}C NMR (126 MHz) spectra of **A1** in $CDCl_3$.

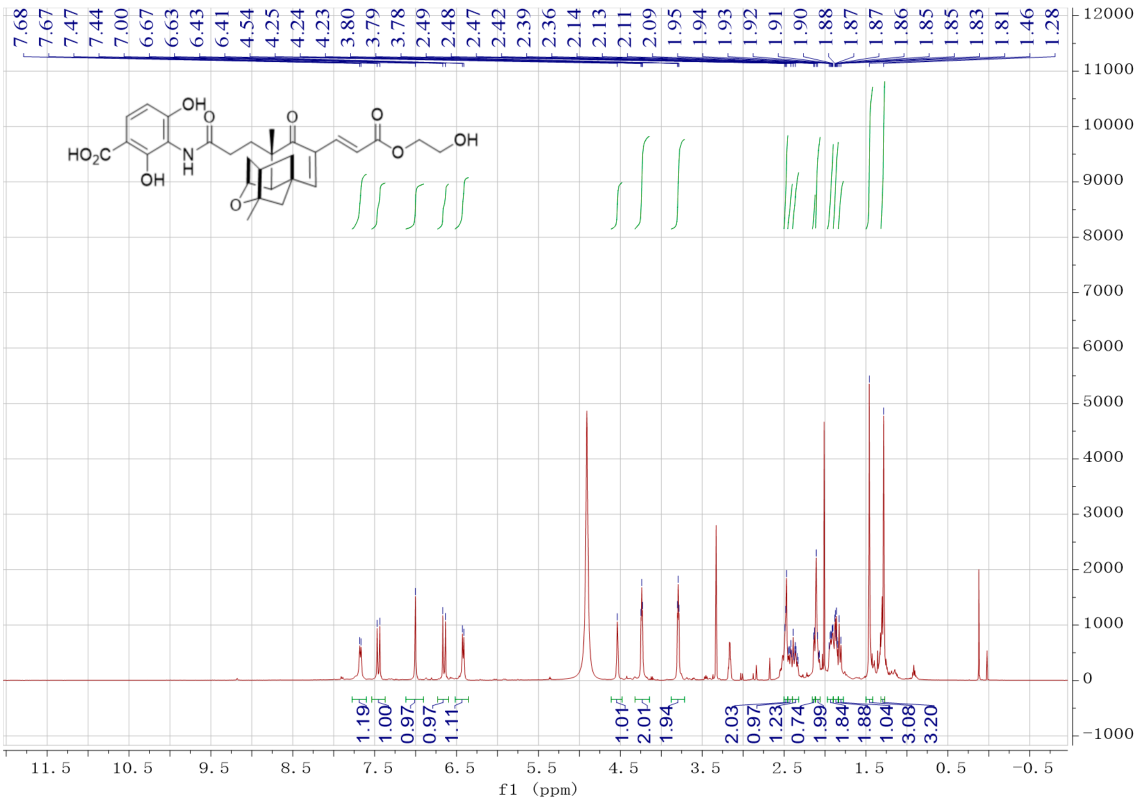

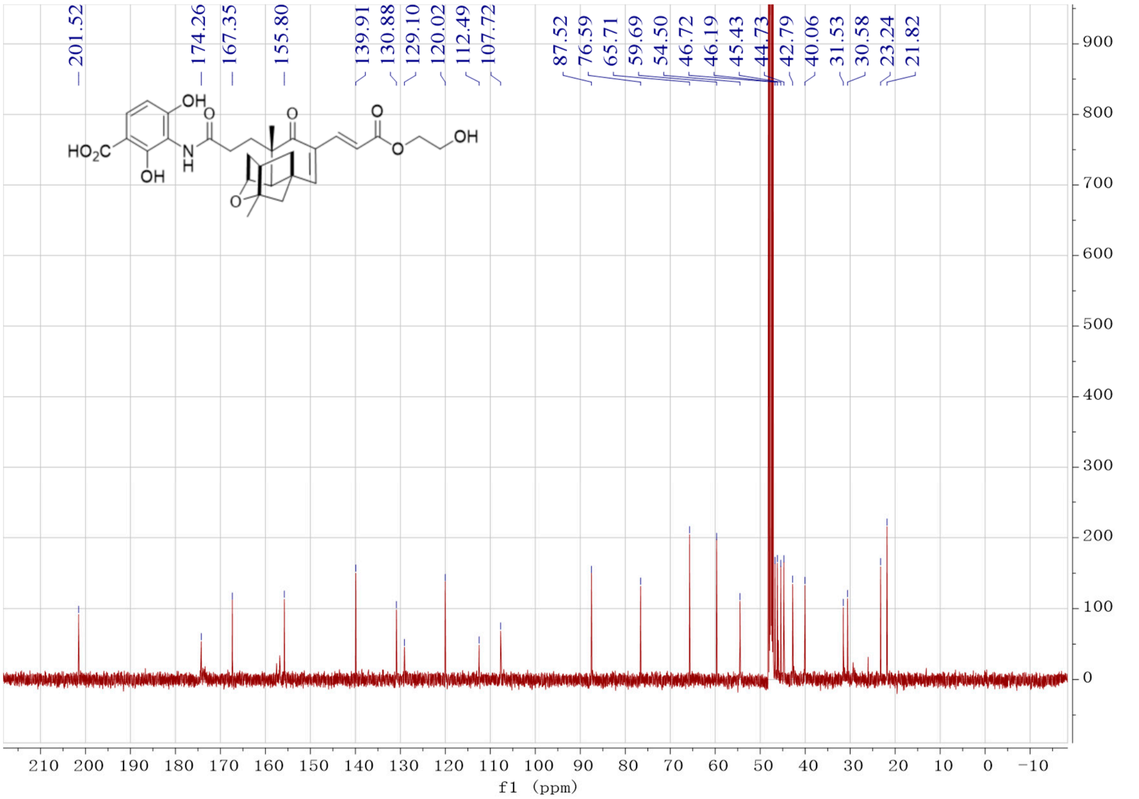

Figure S4. ^{1}H NMR (500 MHz) and ^{13}C NMR (126 MHz) spectra of **A2** in MeOD.

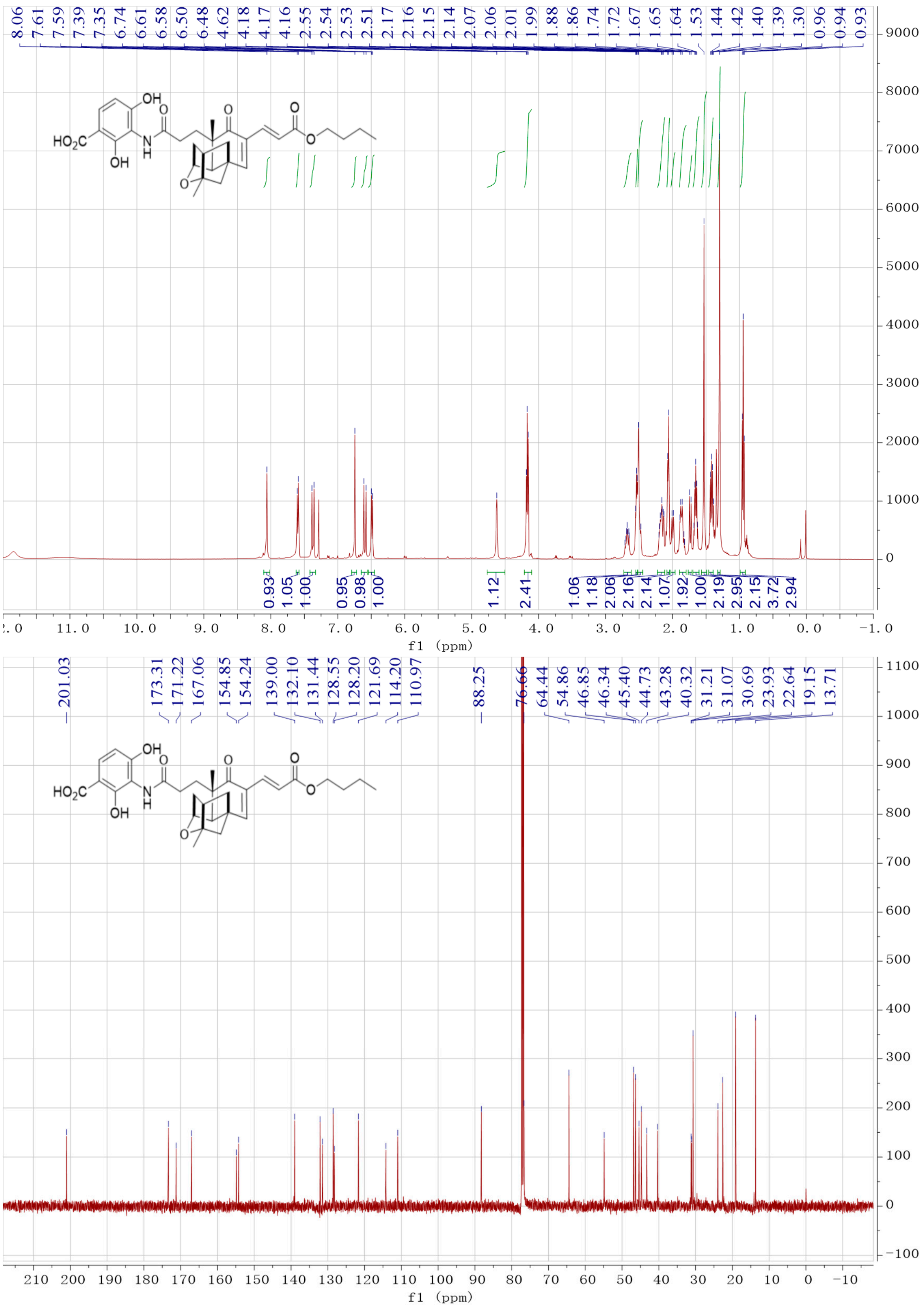

Figure S5. ^{1}H NMR (500 MHz) and ^{13}C NMR (126 MHz) spectra of **A3** in $CDCl_3$.

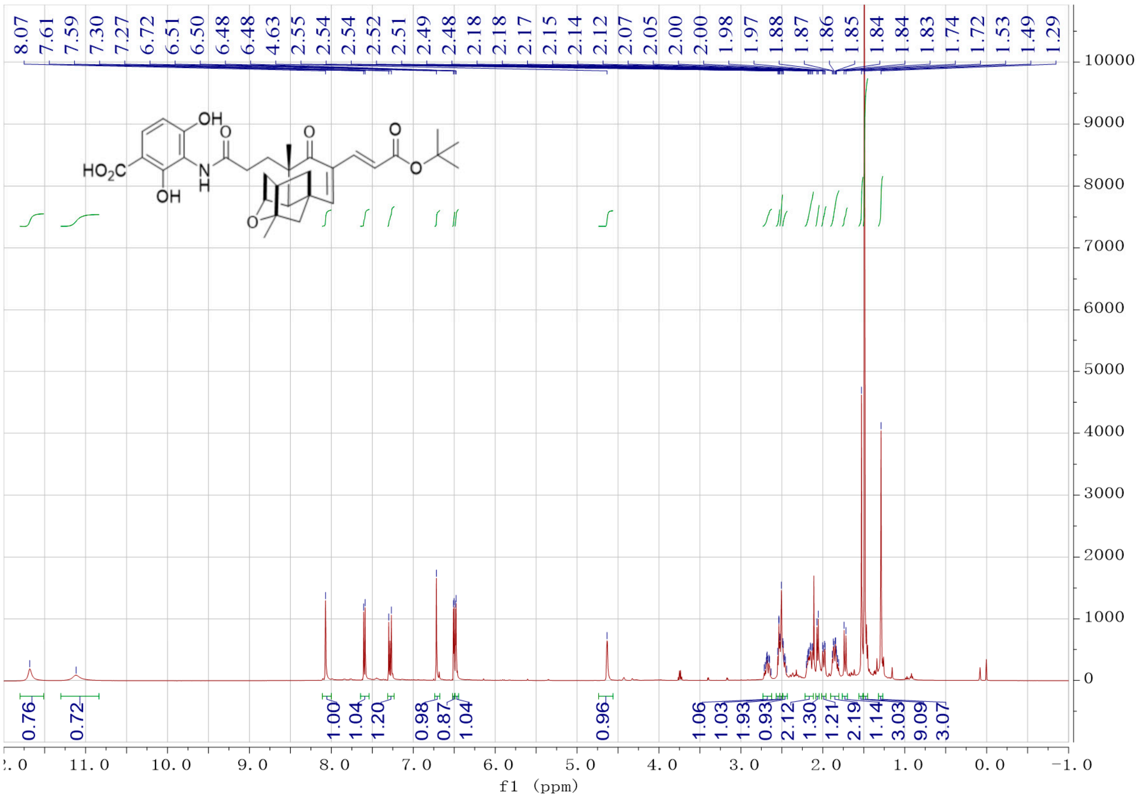

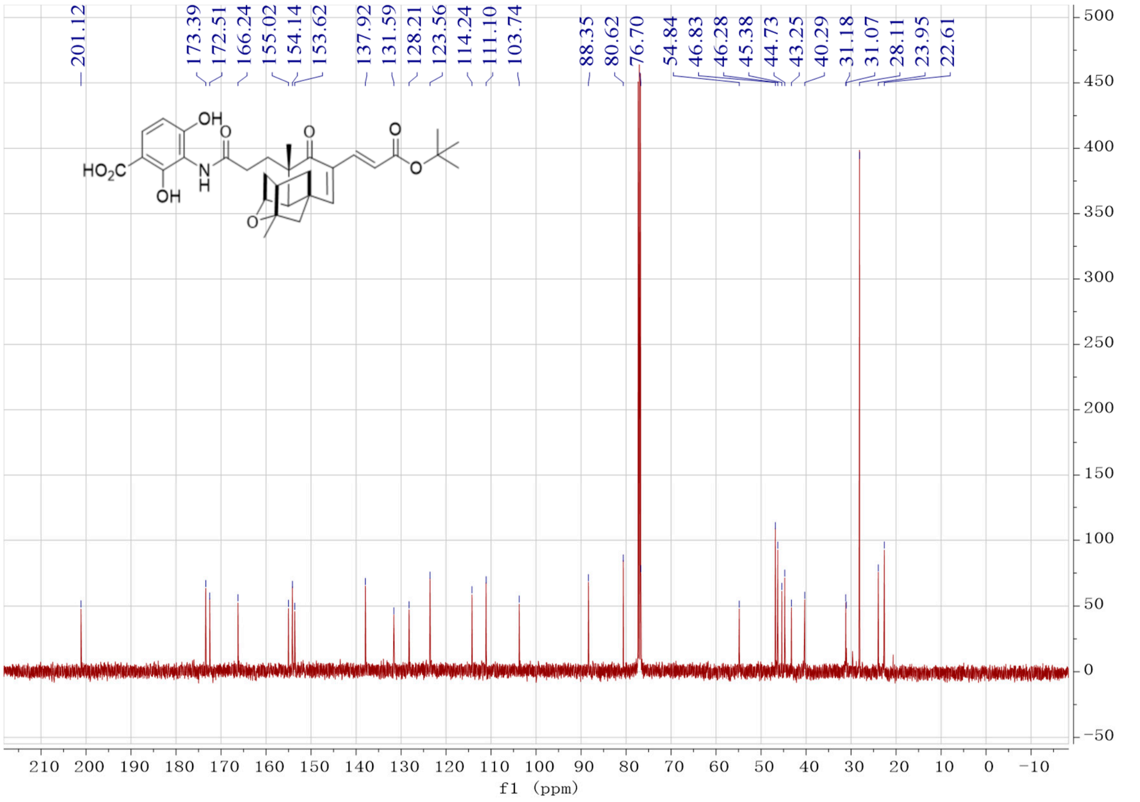

Figure S6. ^{1}H NMR (500 MHz) and ^{13}C NMR (126 MHz) spectra of **A4** in $CDCl_3$.

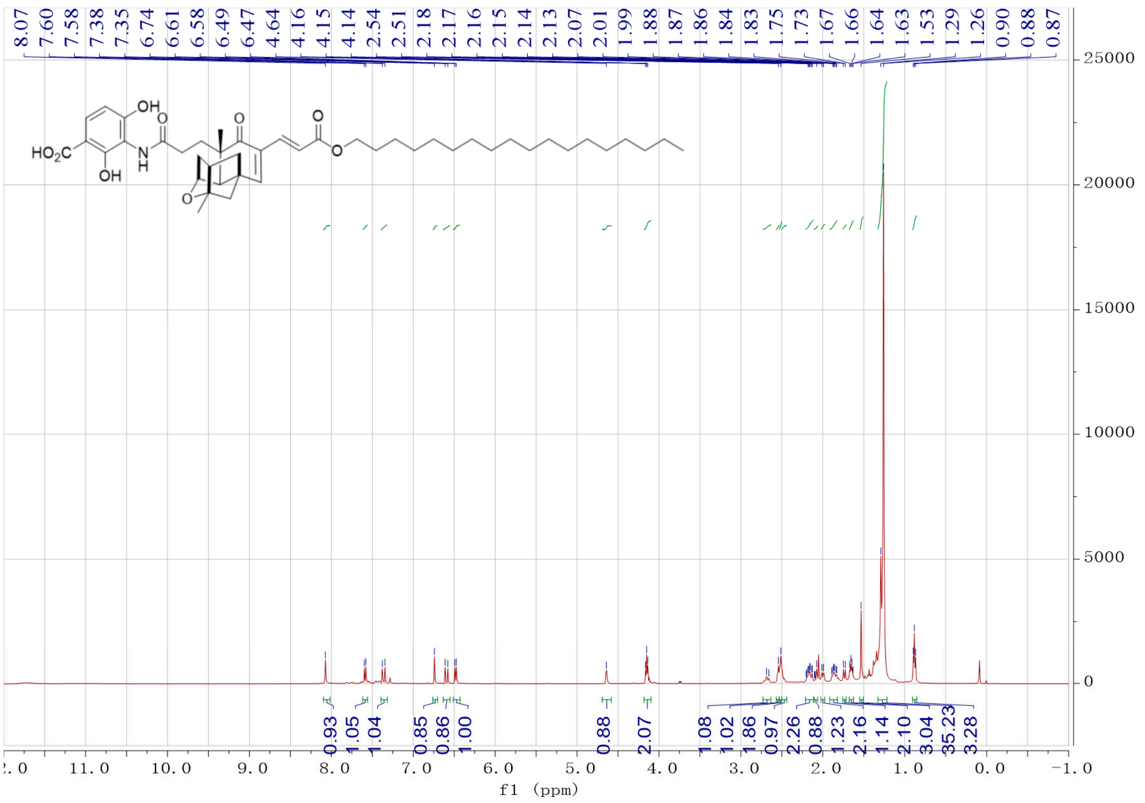

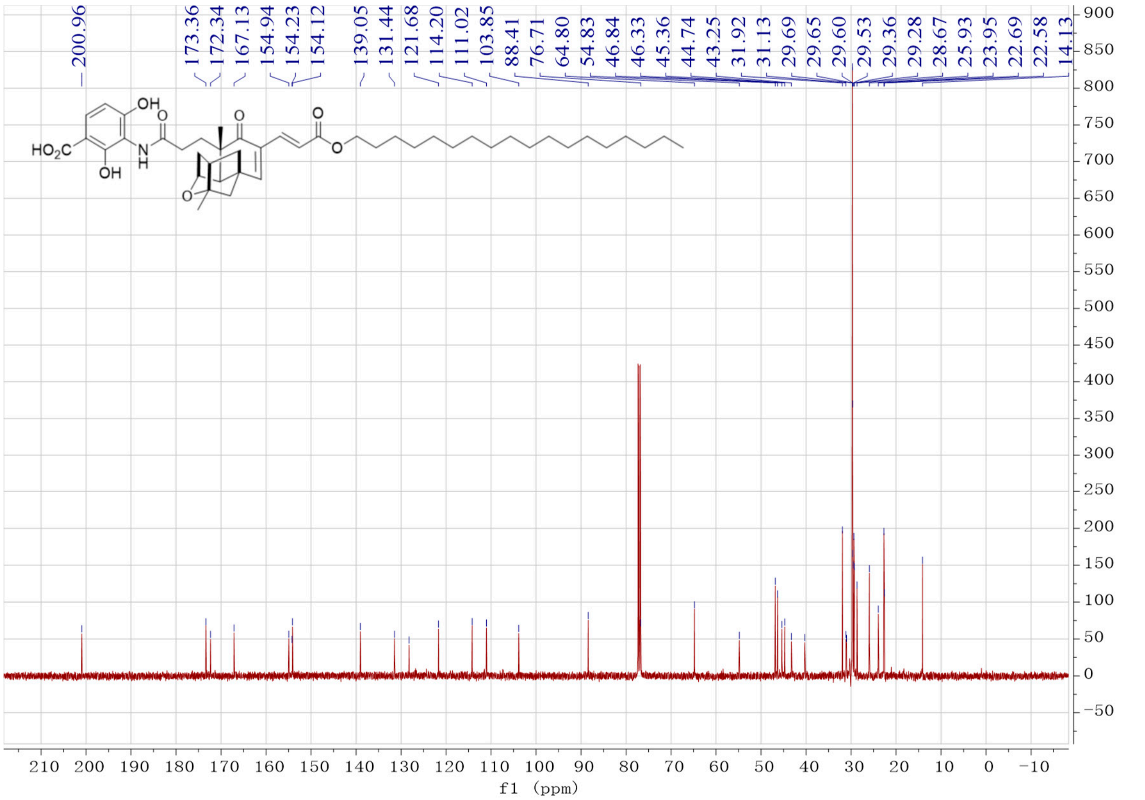

Figure S7. ^{1}H NMR (500 MHz) and ^{13}C NMR (126 MHz) spectra of **A5** in $CDCl_3$.

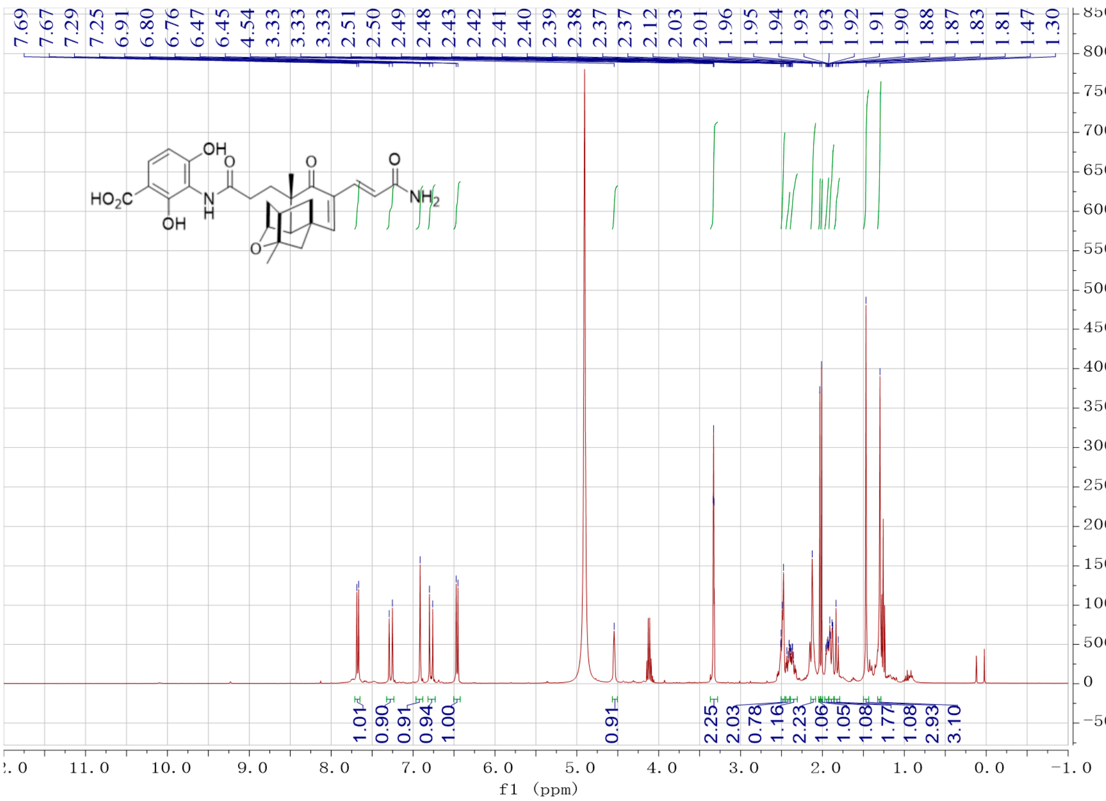

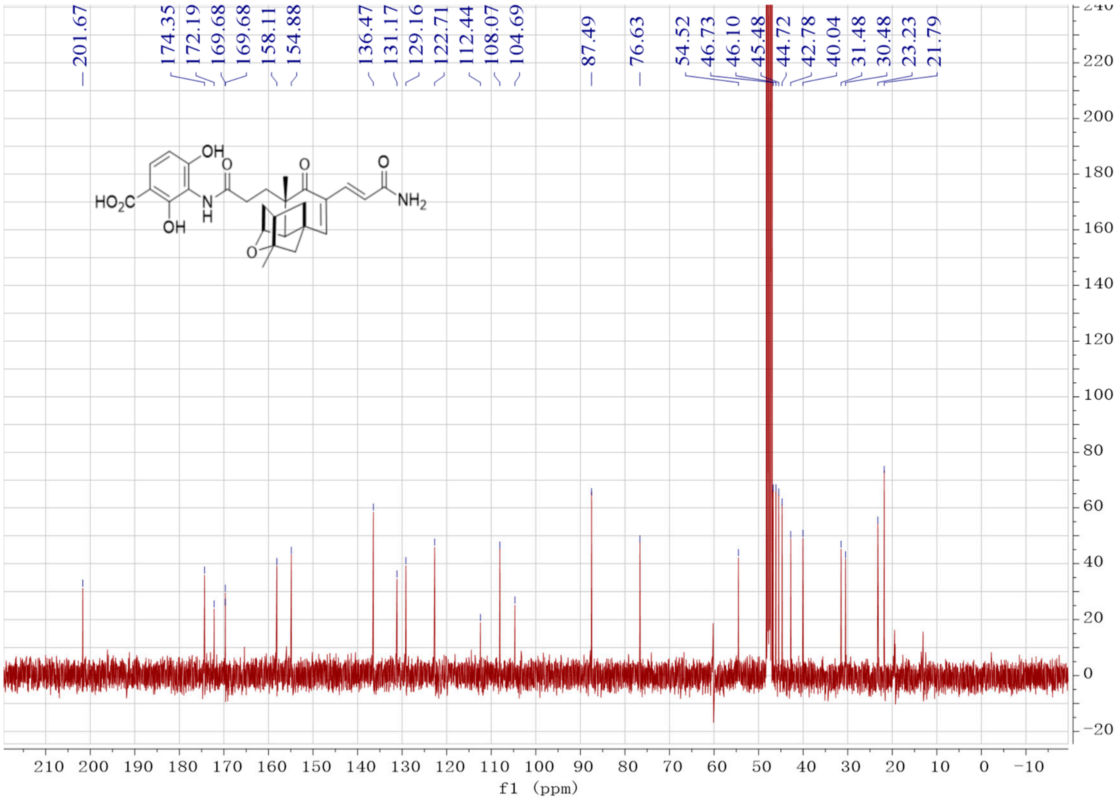

Figure S8. ^{1}H NMR (400 MHz) and ^{13}C NMR (101 MHz) spectra of **A6** in MeOD.

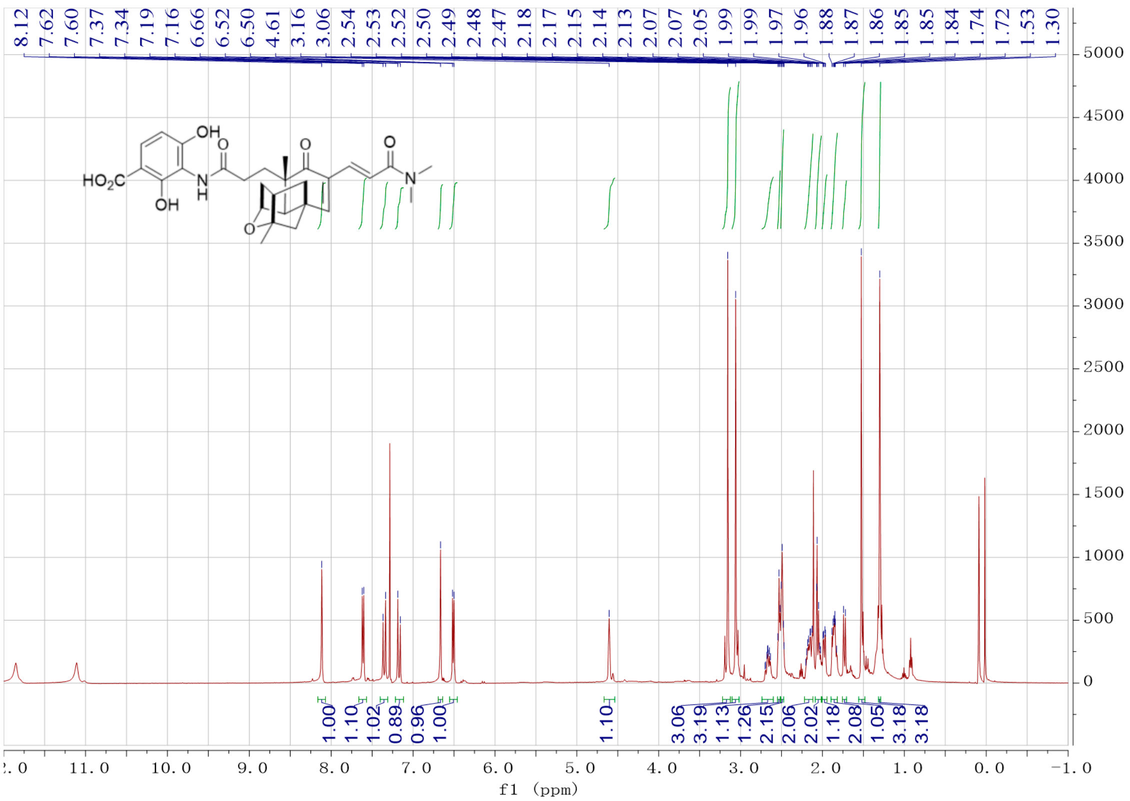

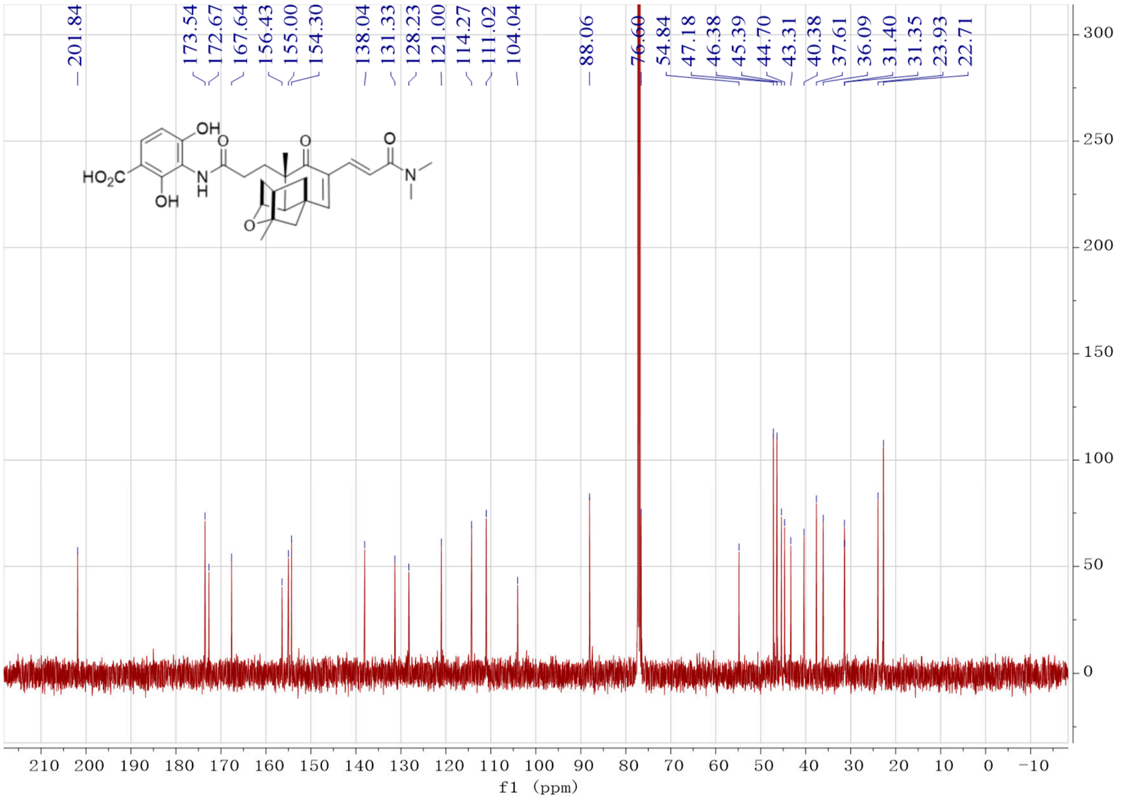

Figure S9. ^{1}H NMR (500 MHz) and ^{13}C NMR (126 MHz) spectra of **A7** in $CDCl_3$.

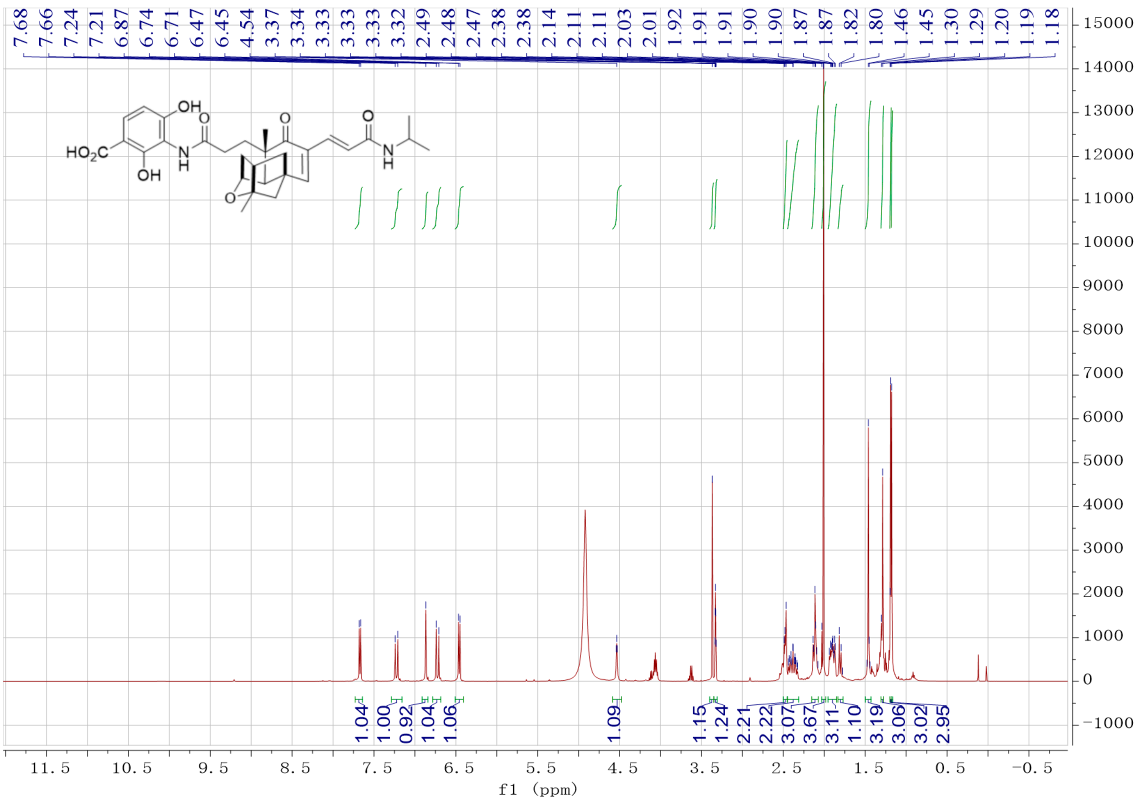

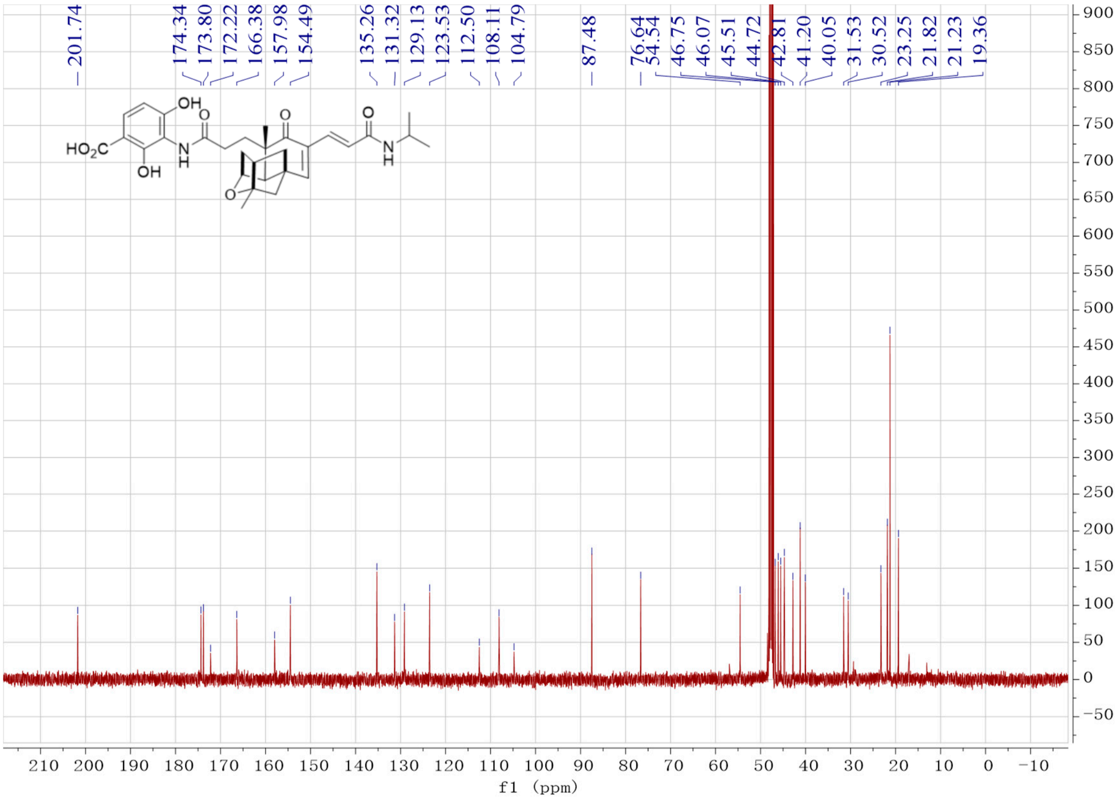

Figure S10. ^{1}H NMR (500 MHz) and ^{13}C NMR (126 MHz) spectra of **A8** in MeOD.

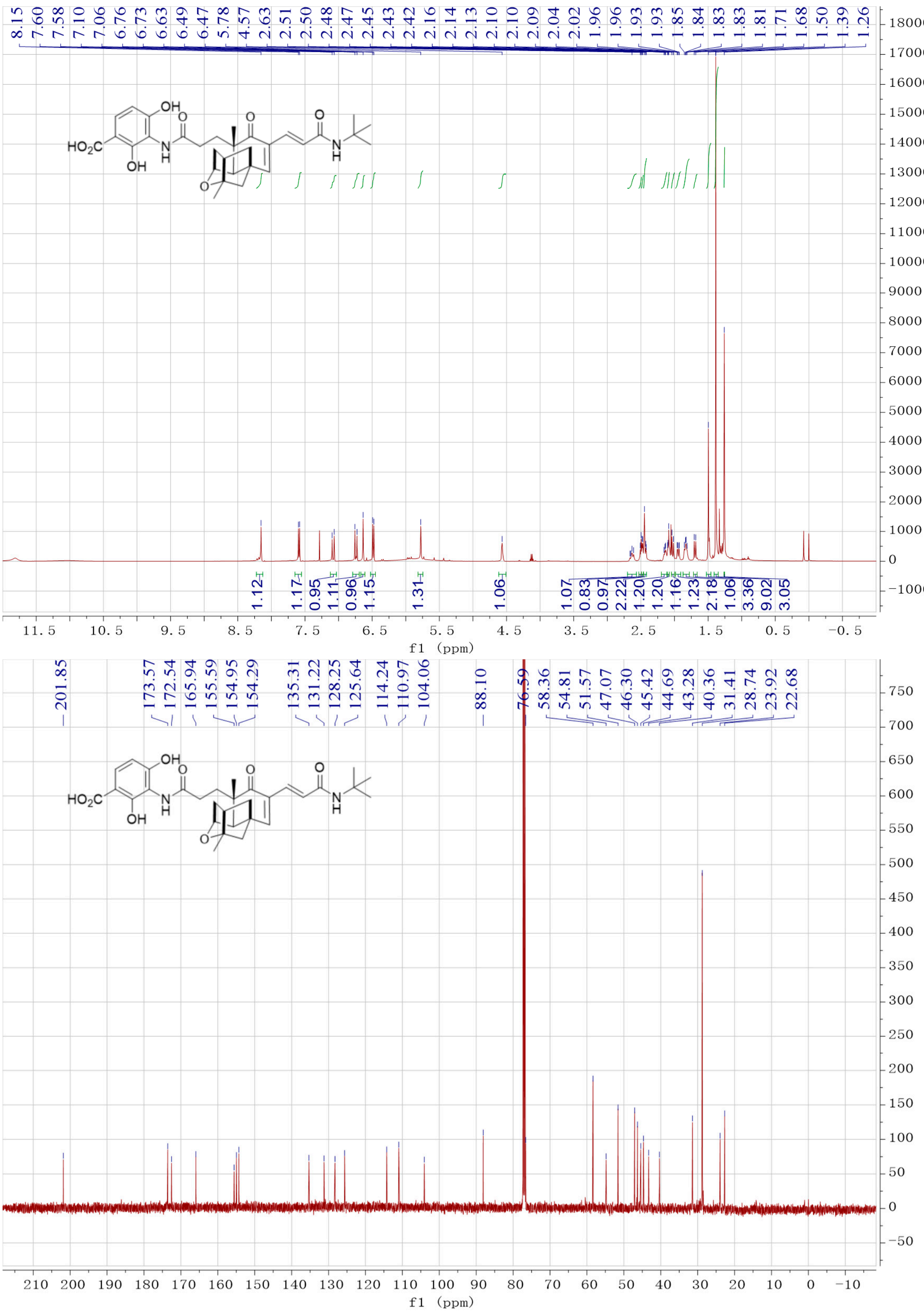

Figure S11. ^{1}H NMR (500 MHz) and ^{13}C NMR (126 MHz) spectra of **A9** in $CDCl_3$.

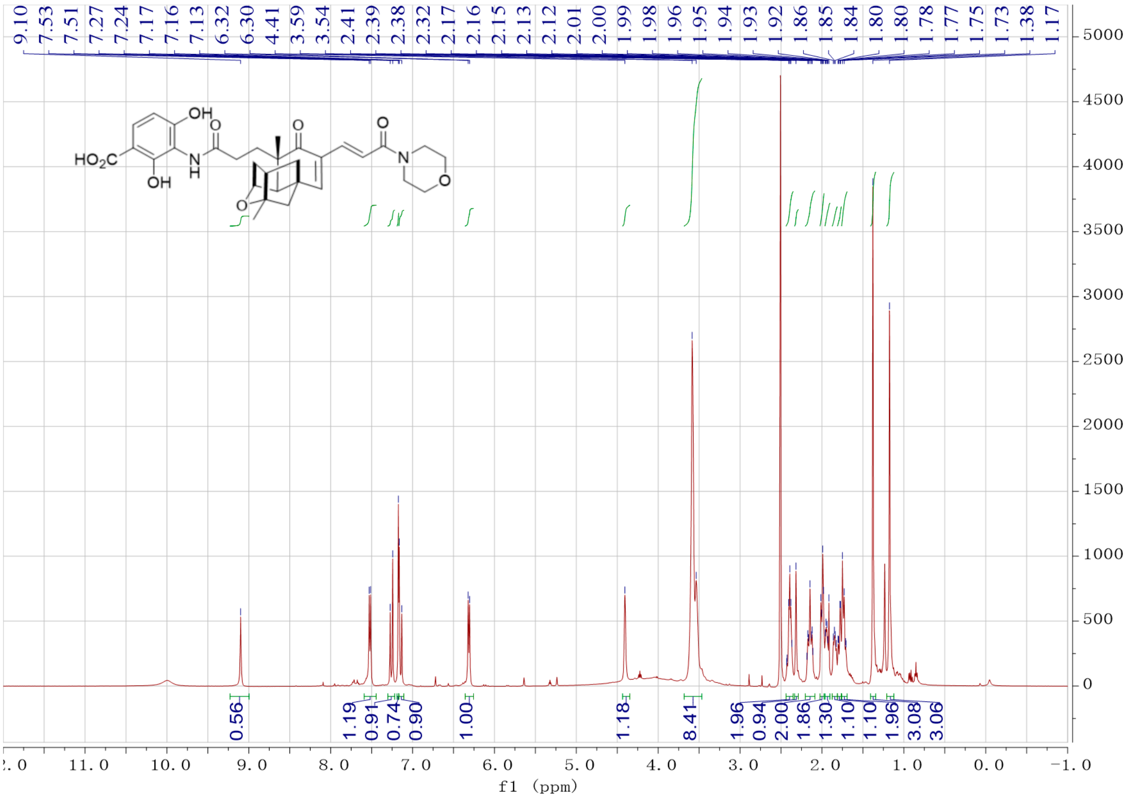

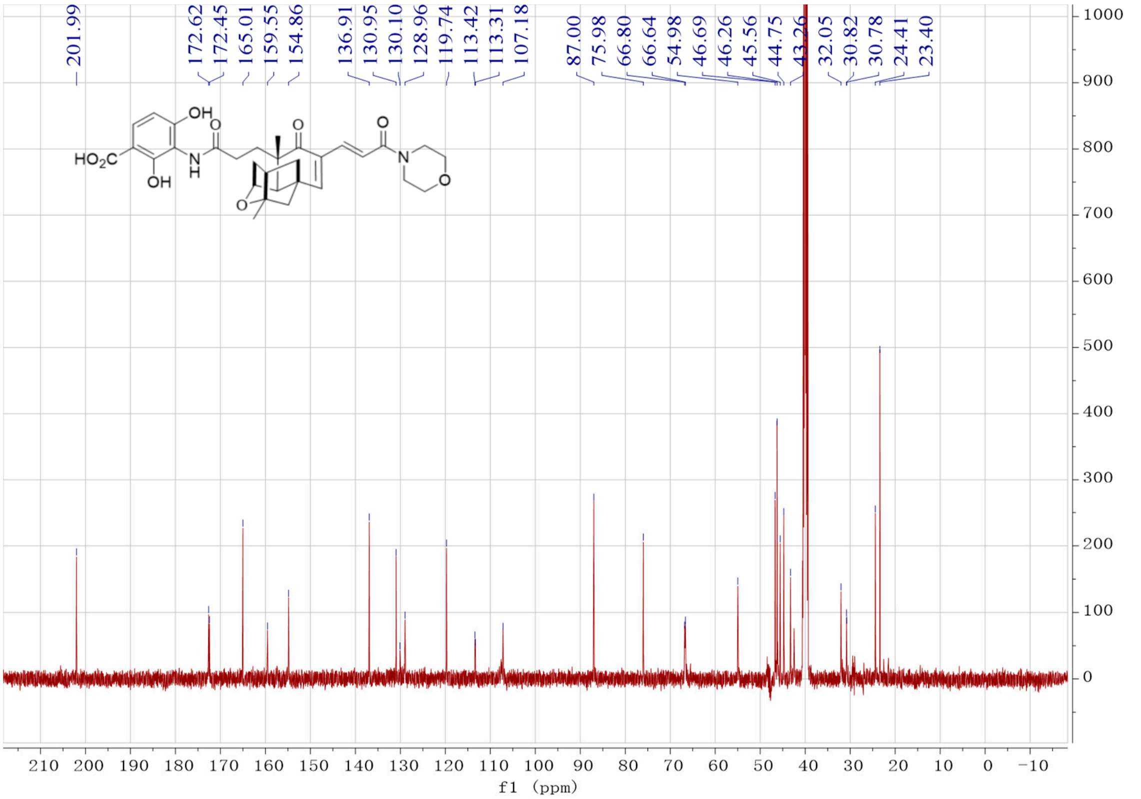

Figure S12. ^{1}H NMR (500 MHz) and ^{13}C NMR (126 MHz) spectra of **A10** in DMSO-d_6.

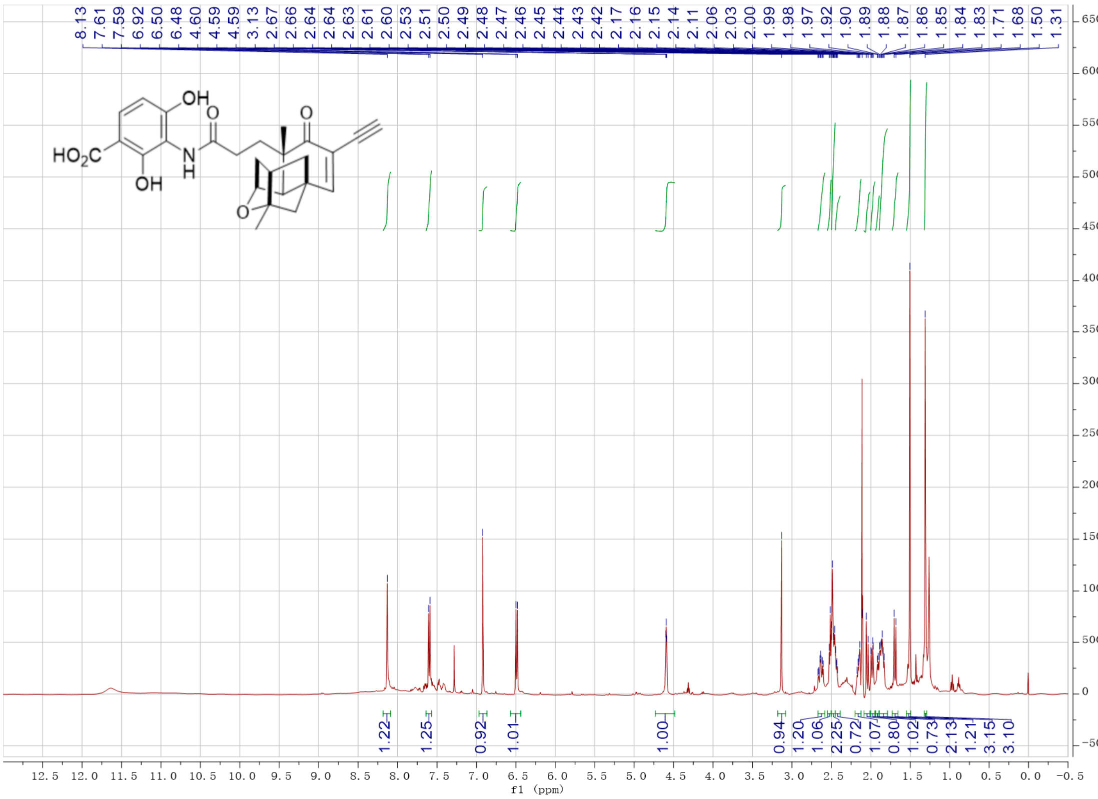

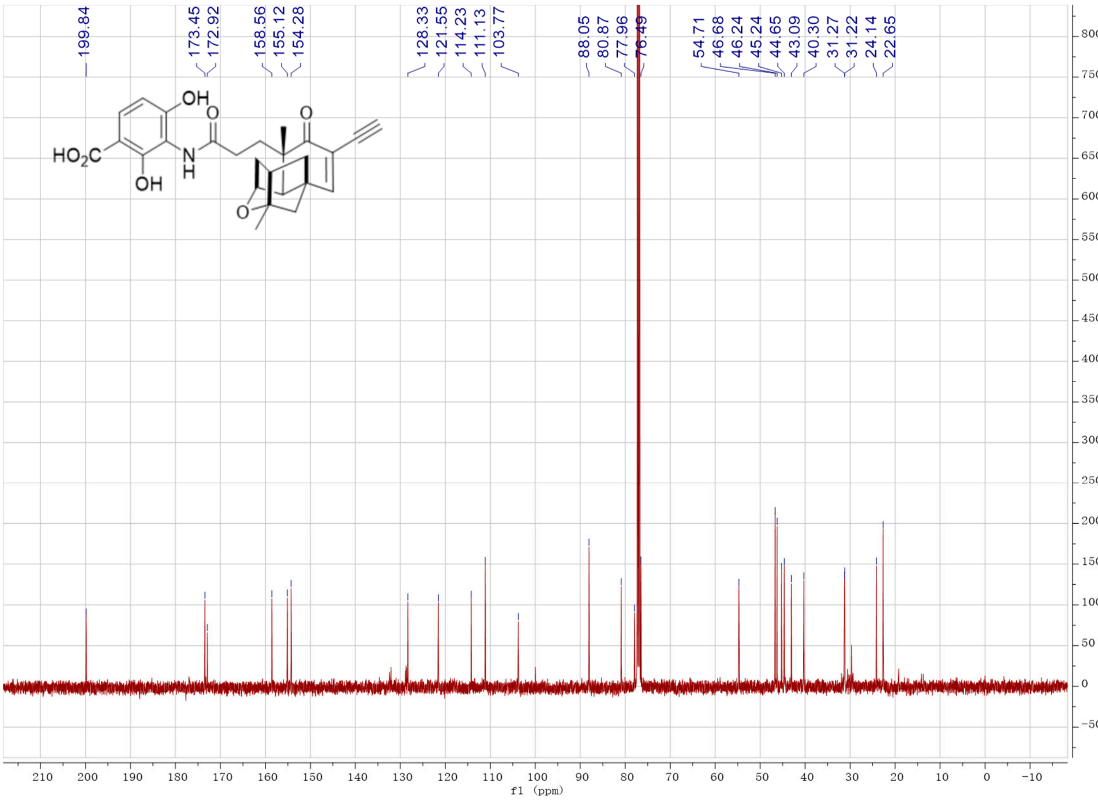

Figure S13. ^{1}H NMR (500 MHz) and ^{13}C NMR (126 MHz) spectra of **B1** in $CDCl_3$.

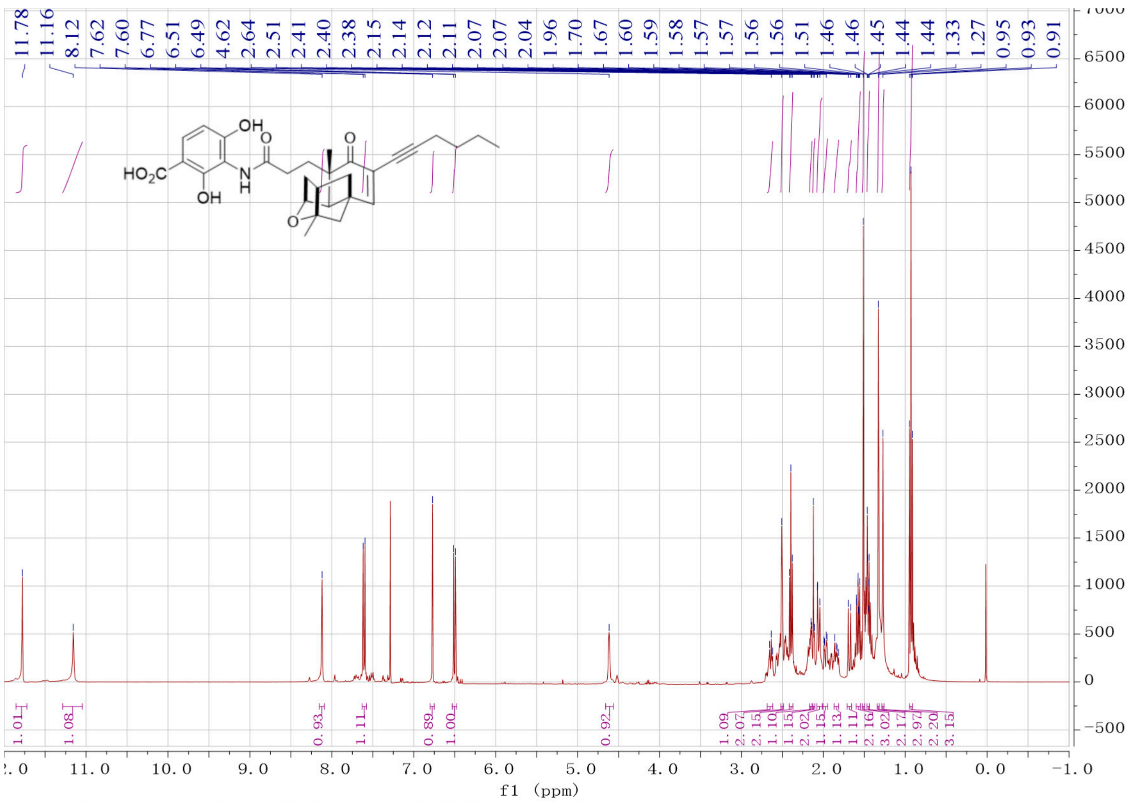

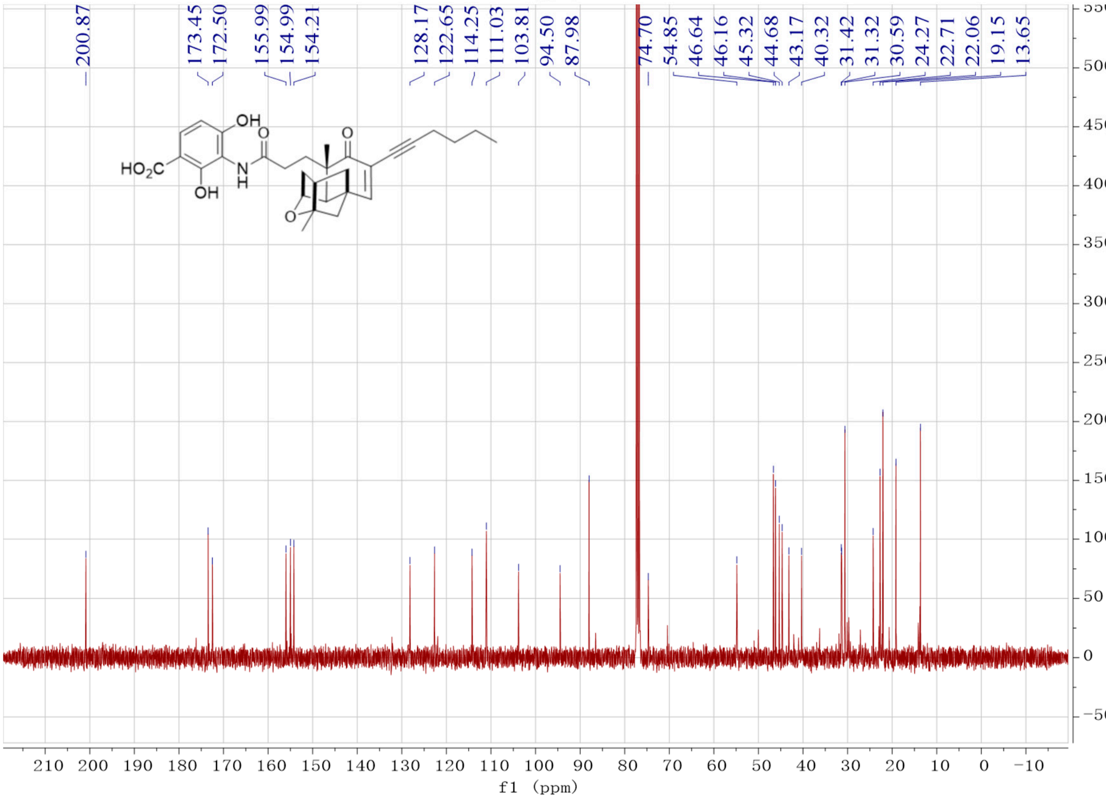

Figure S14. ^{1}H NMR (400 MHz) and ^{13}C NMR (101 MHz) spectra of **B2** in $CDCl_3$.

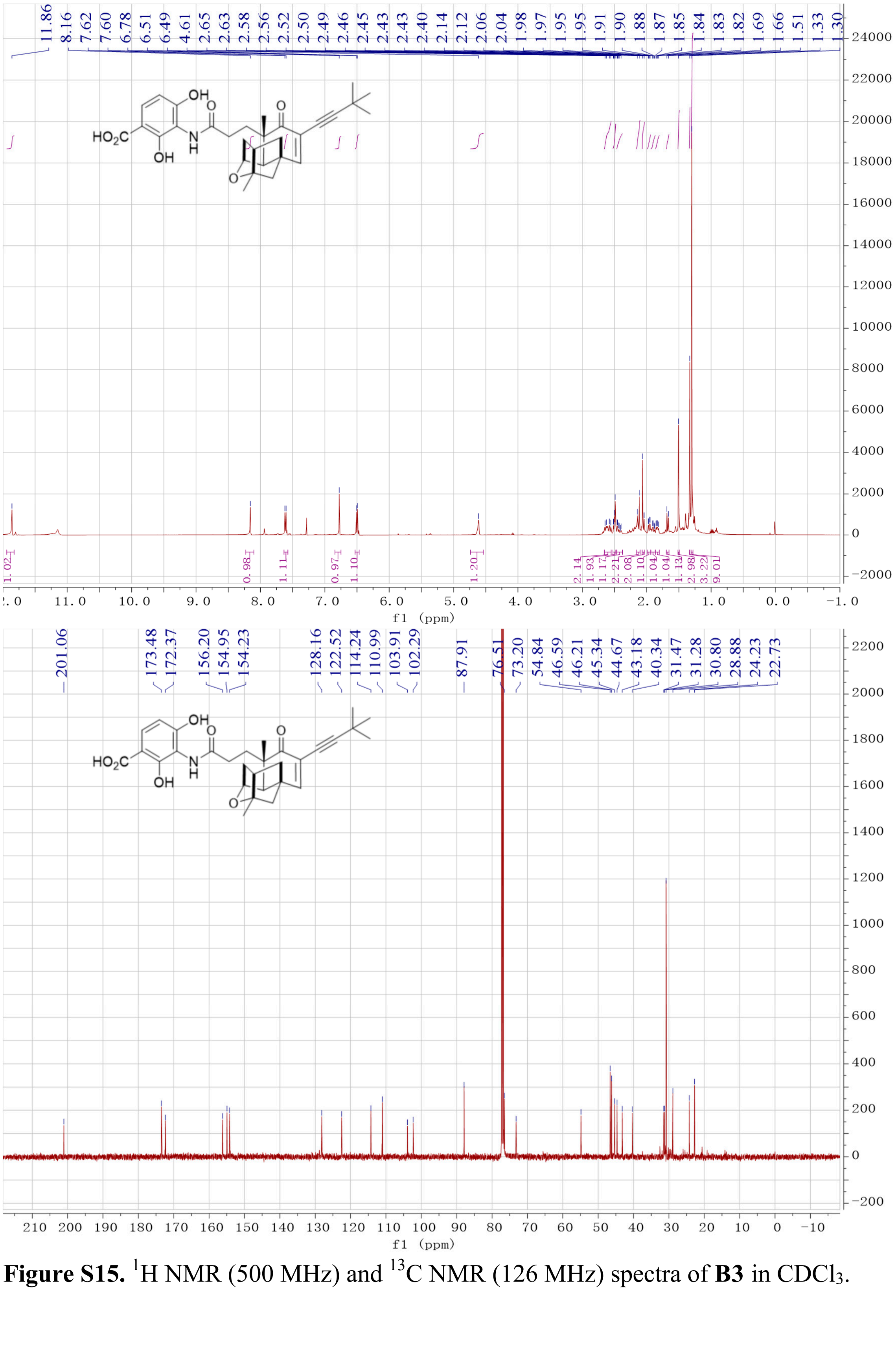

Figure S15. ^{1}H NMR (500 MHz) and ^{13}C NMR (126 MHz) spectra of **B3** in $CDCl_3$.

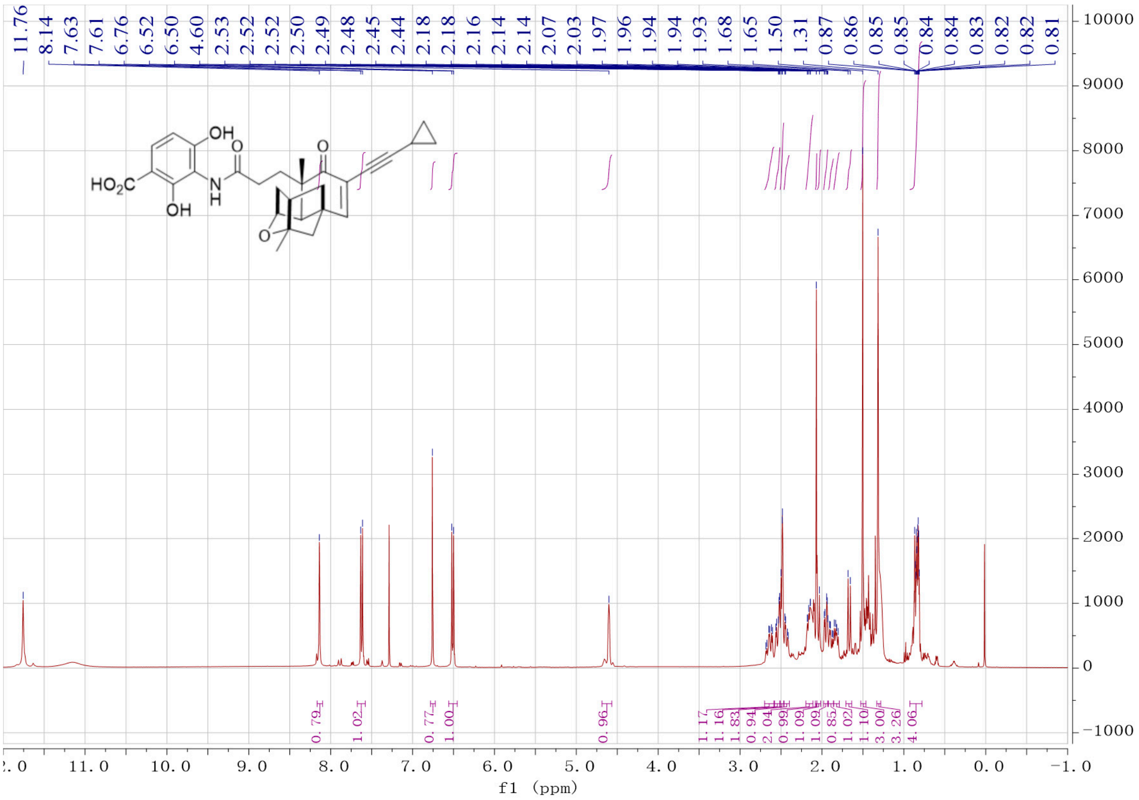

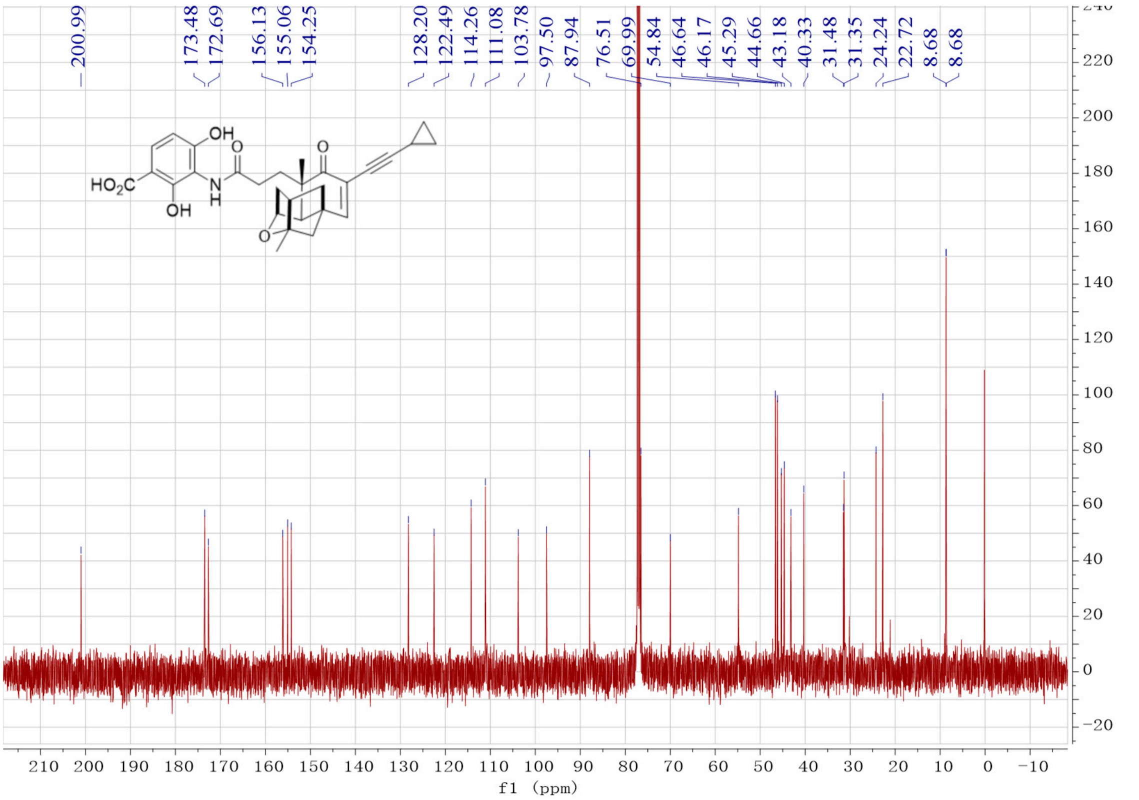

Figure S16. ^{1}H NMR (400 MHz) and ^{13}C NMR (126 MHz) spectra of **B4** in $CDCl_3$.

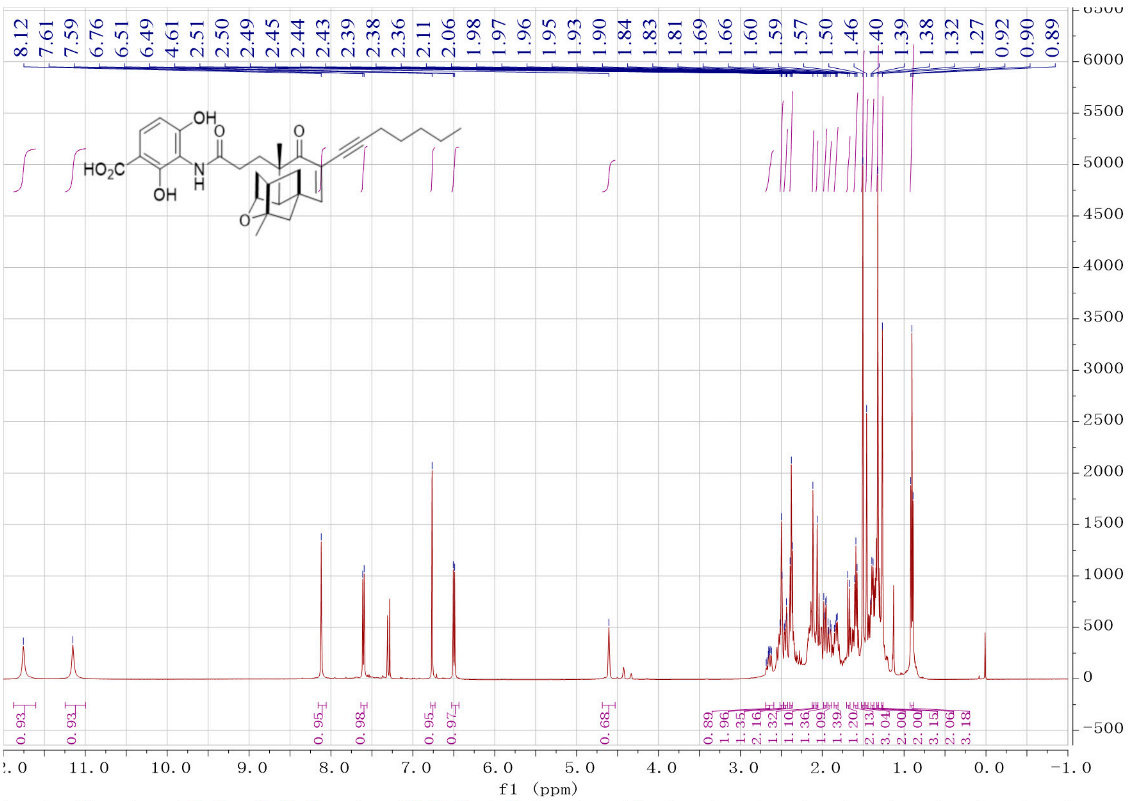

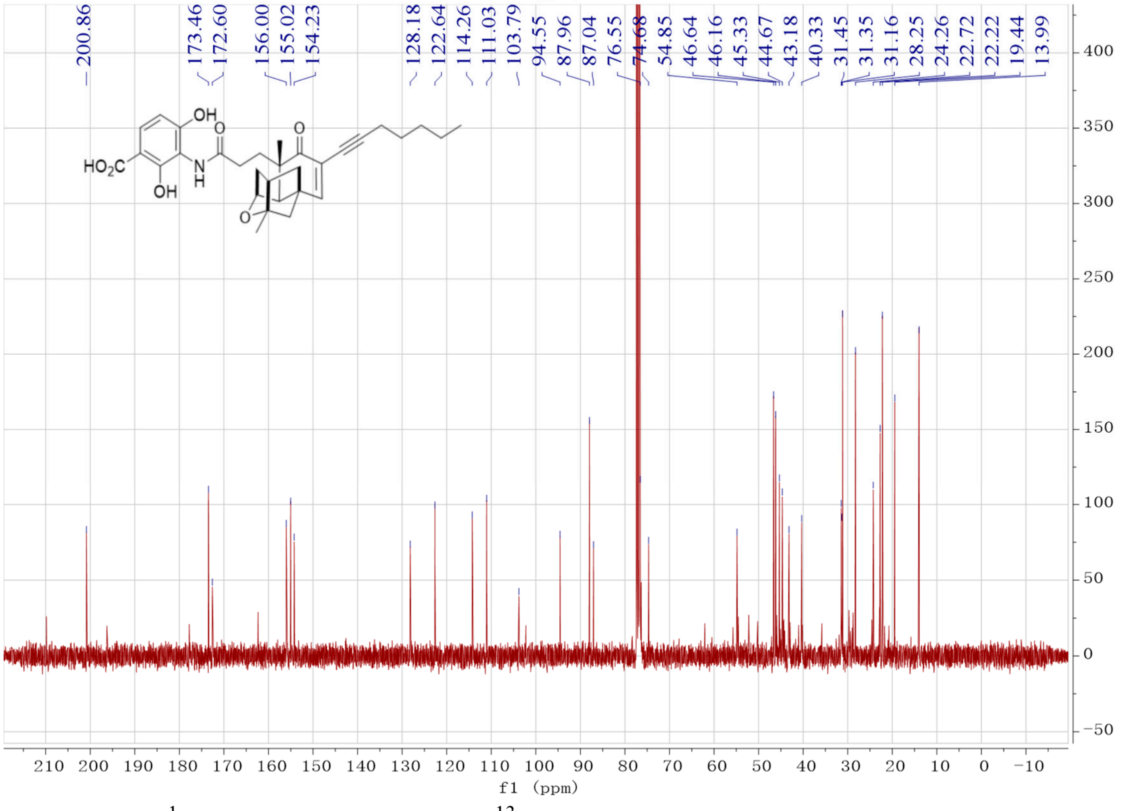

Figure S17. ^{1}H NMR (500 MHz) and ^{13}C NMR (101 MHz) spectra of **B5** in $CDCl_3$.

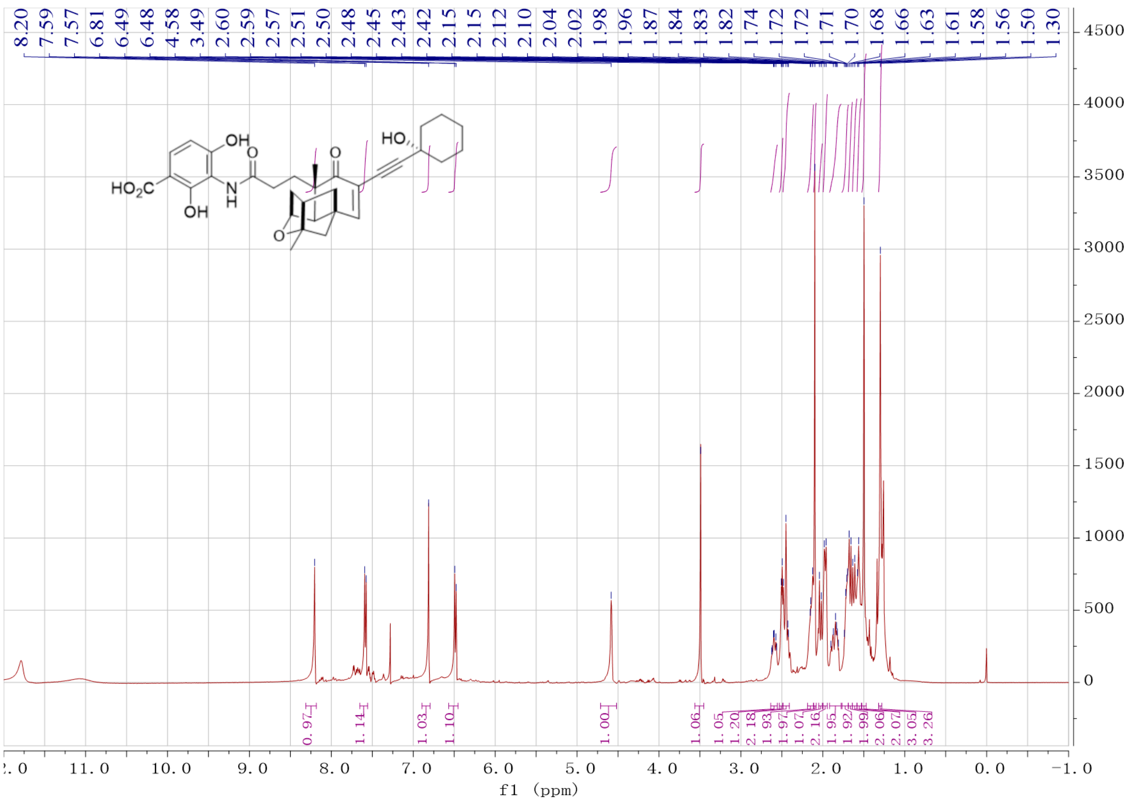

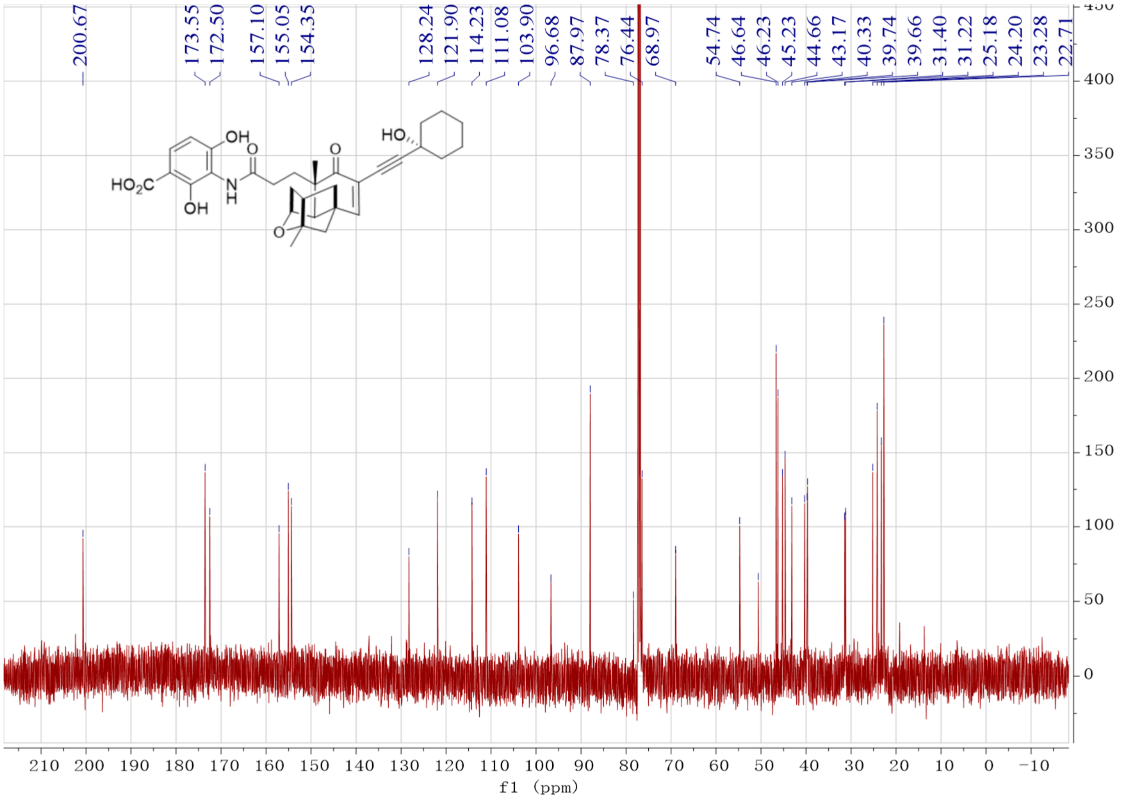

Figure S18. ^{1}H NMR (500 MHz) and ^{13}C NMR (126 MHz) spectra of **B6** in $CDCl_3$.

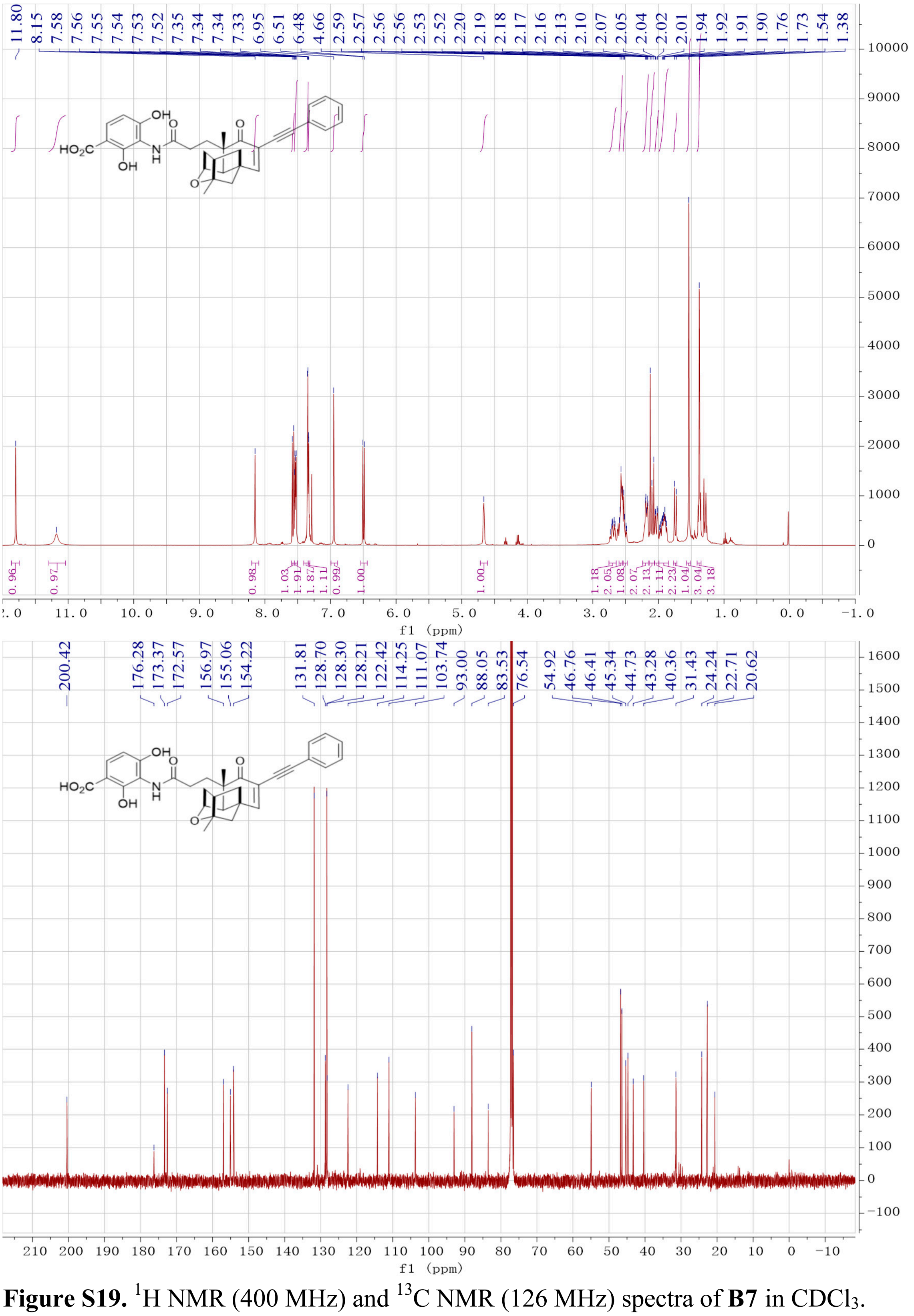

Figure S19. ^{1}H NMR (400 MHz) and ^{13}C NMR (126 MHz) spectra of **B7** in $CDCl_3$.

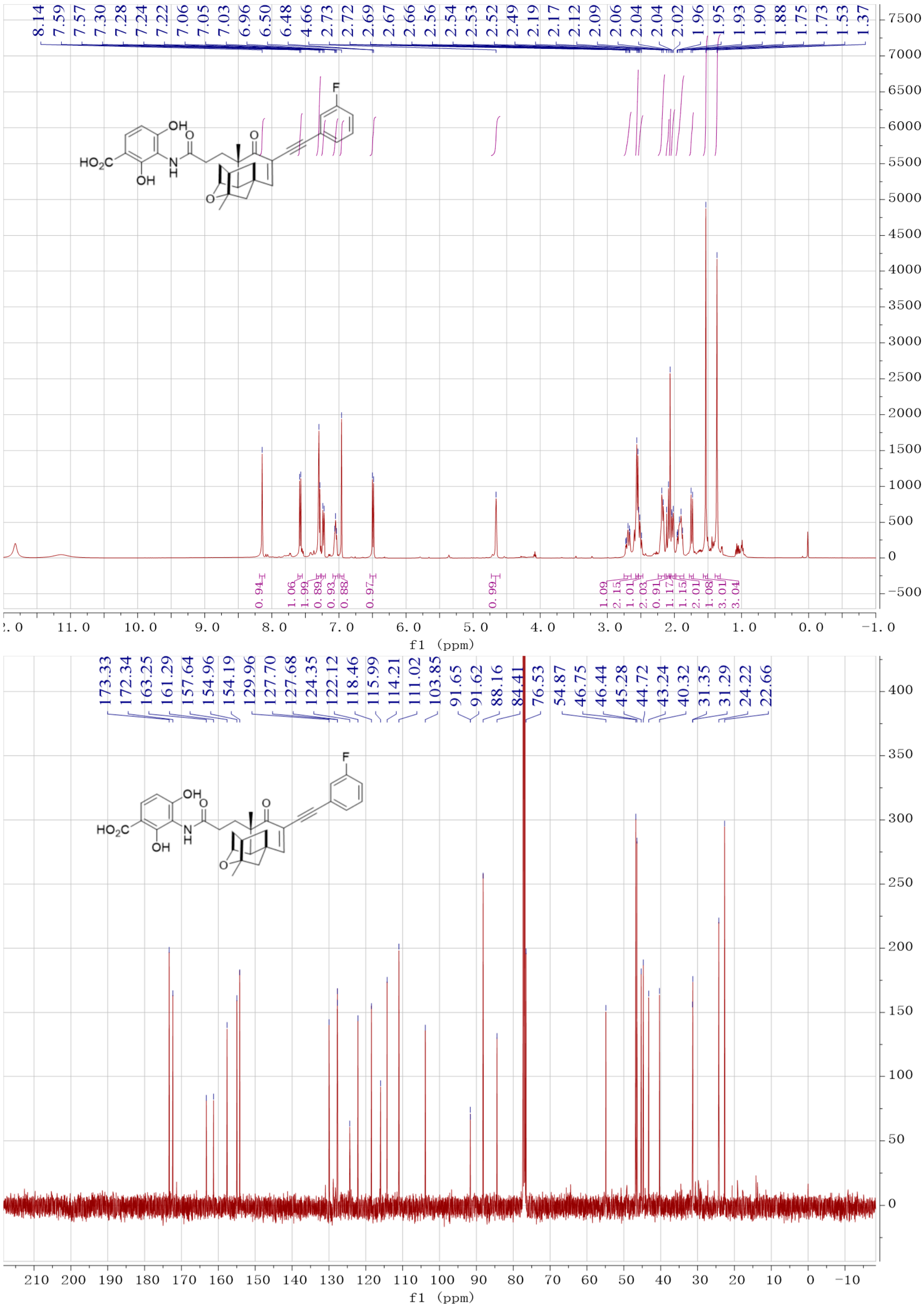

Figure S20. ^{1}H NMR (500 MHz) and ^{13}C NMR (126 MHz) spectra of **B8** in $CDCl_3$.

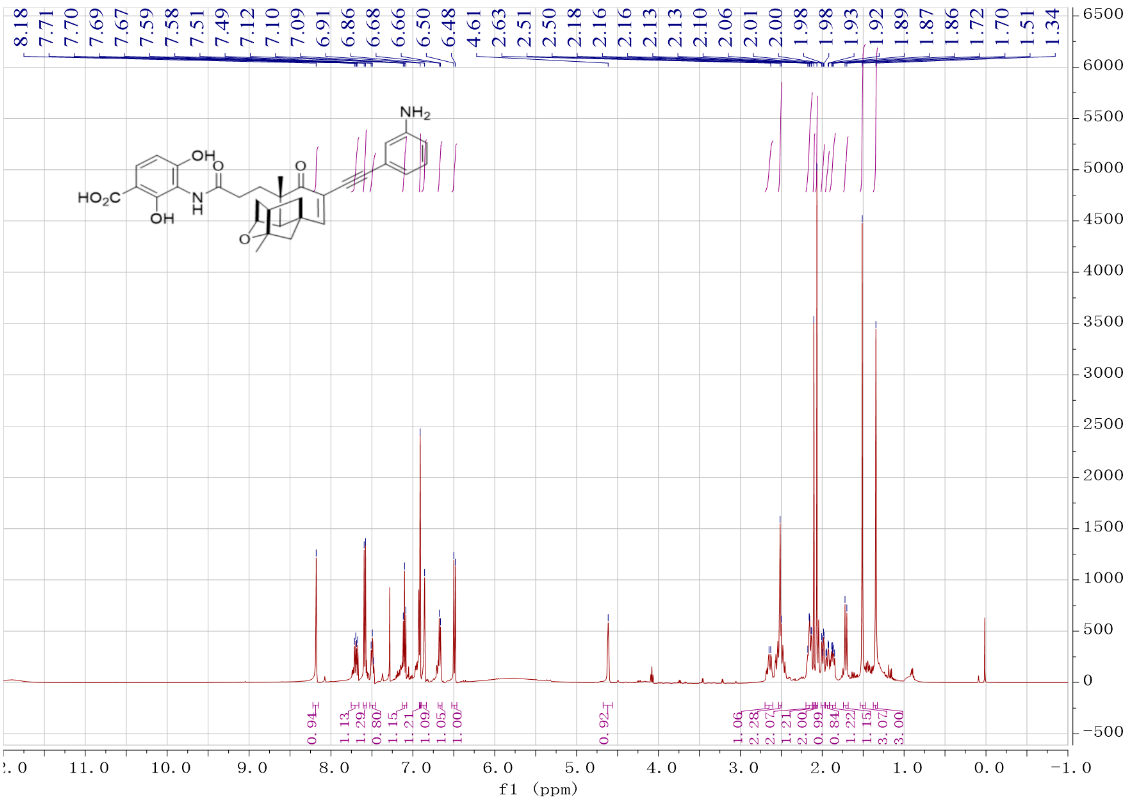

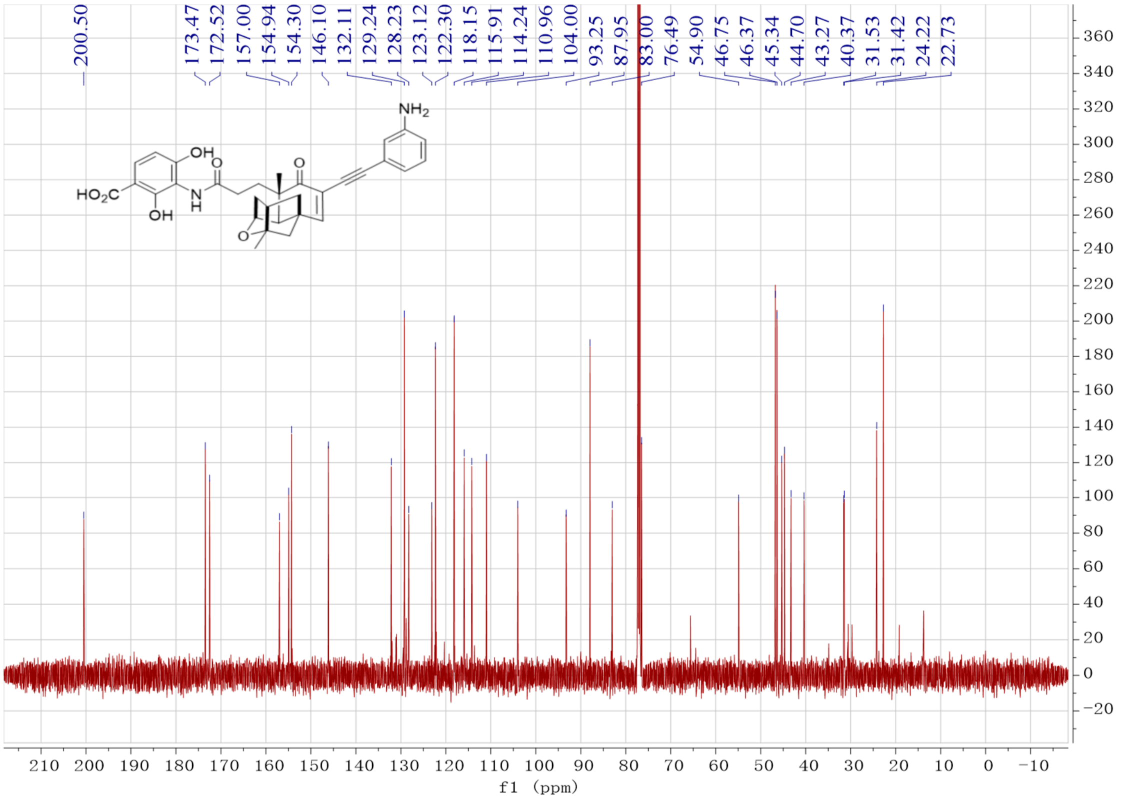

Figure S21. ^{1}H NMR (500 MHz) and ^{13}C NMR (126 MHz) spectra of **B9** in $CDCl_3$.

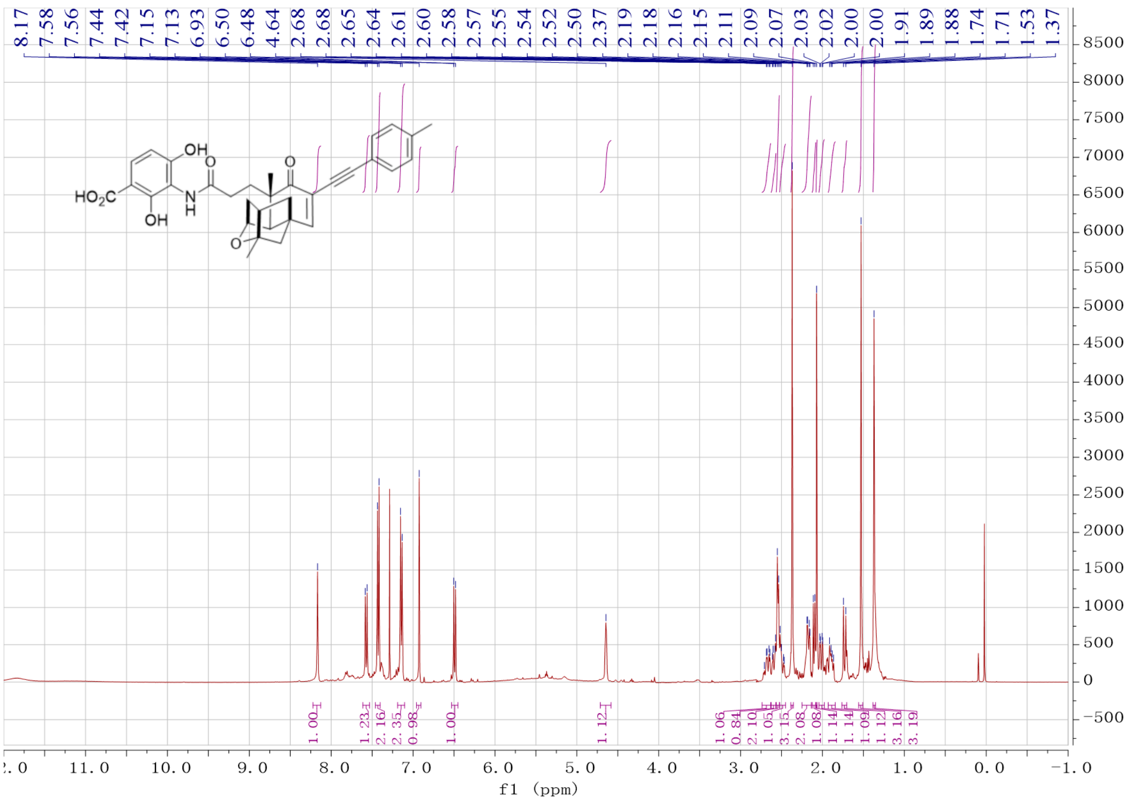

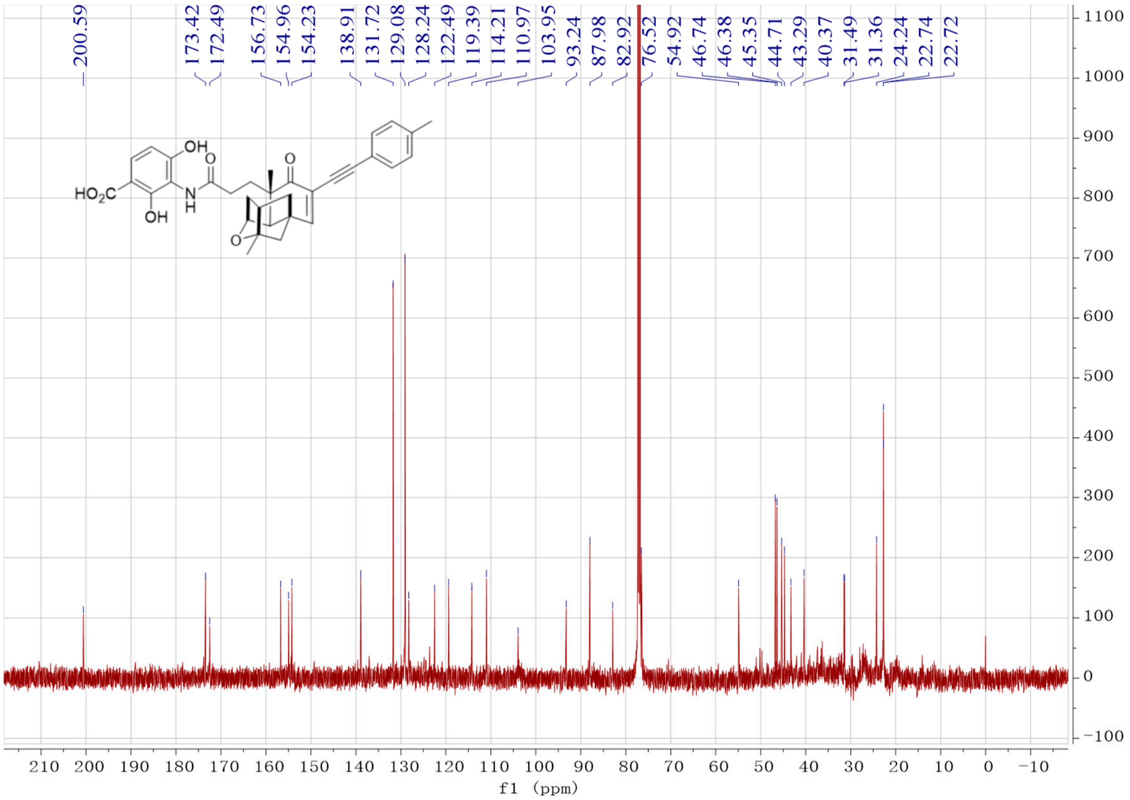

Figure S22. ^{1}H NMR (400 MHz) and ^{13}C NMR (126 MHz) spectra of **B10** in $CDCl_3$.

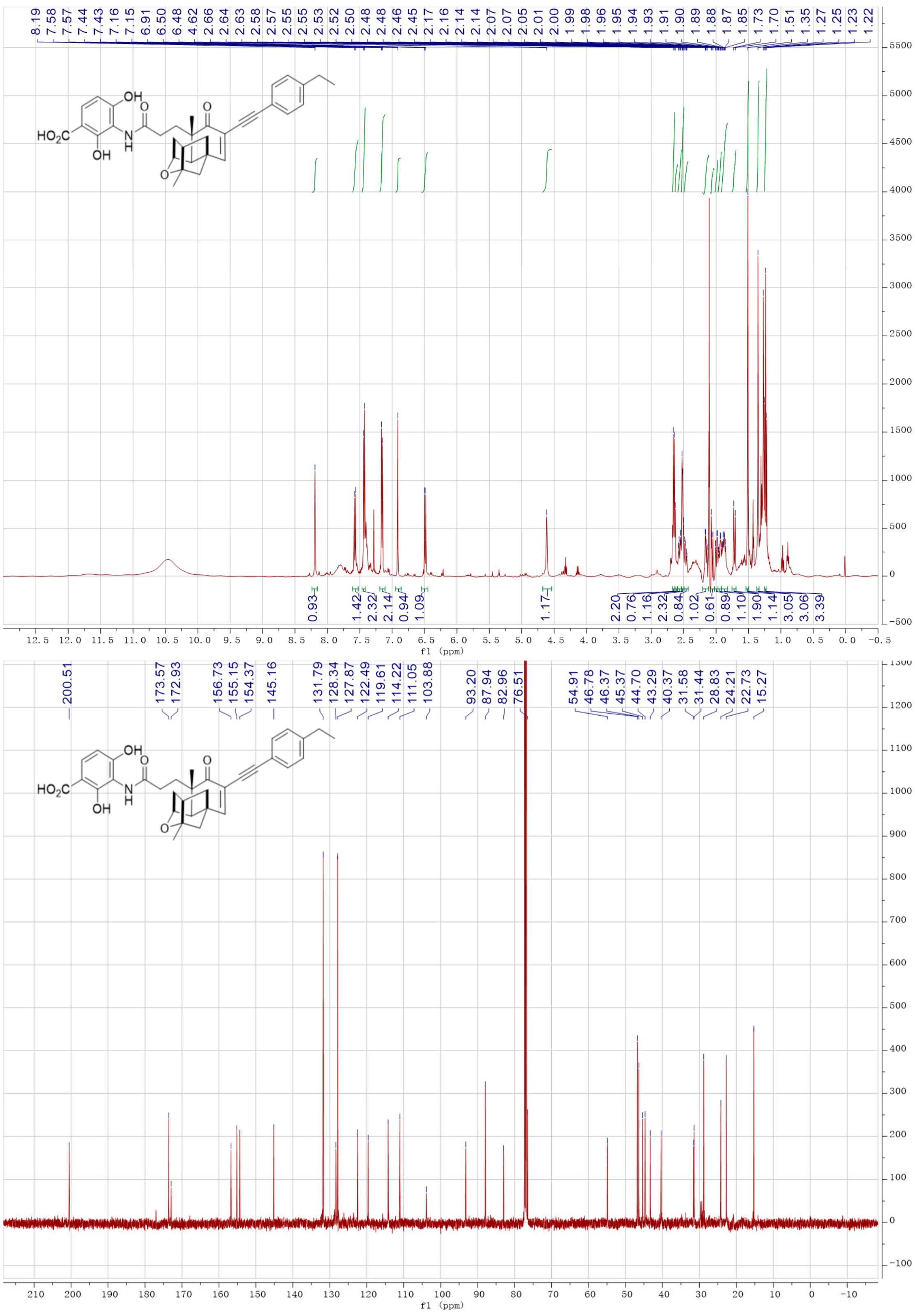

Figure S23. ^{1}H NMR (500 MHz) and ^{13}C NMR (126 MHz) spectra of **B11** in $CDCl_3$.

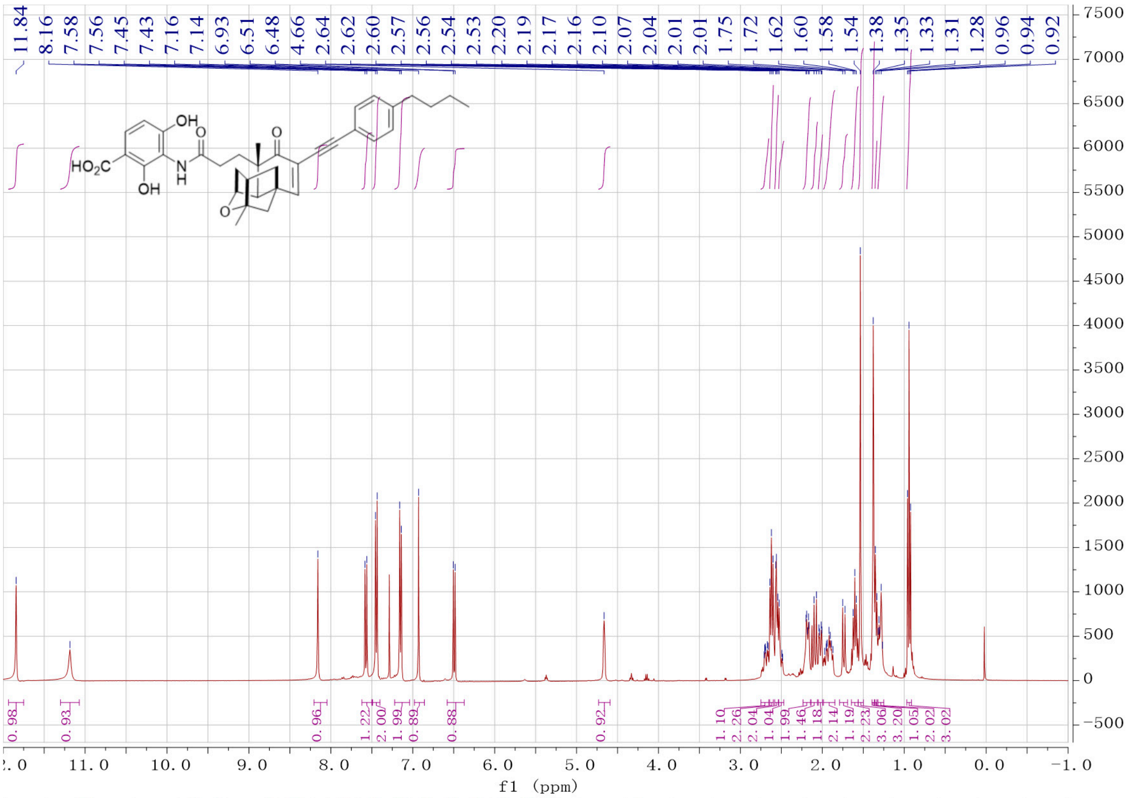

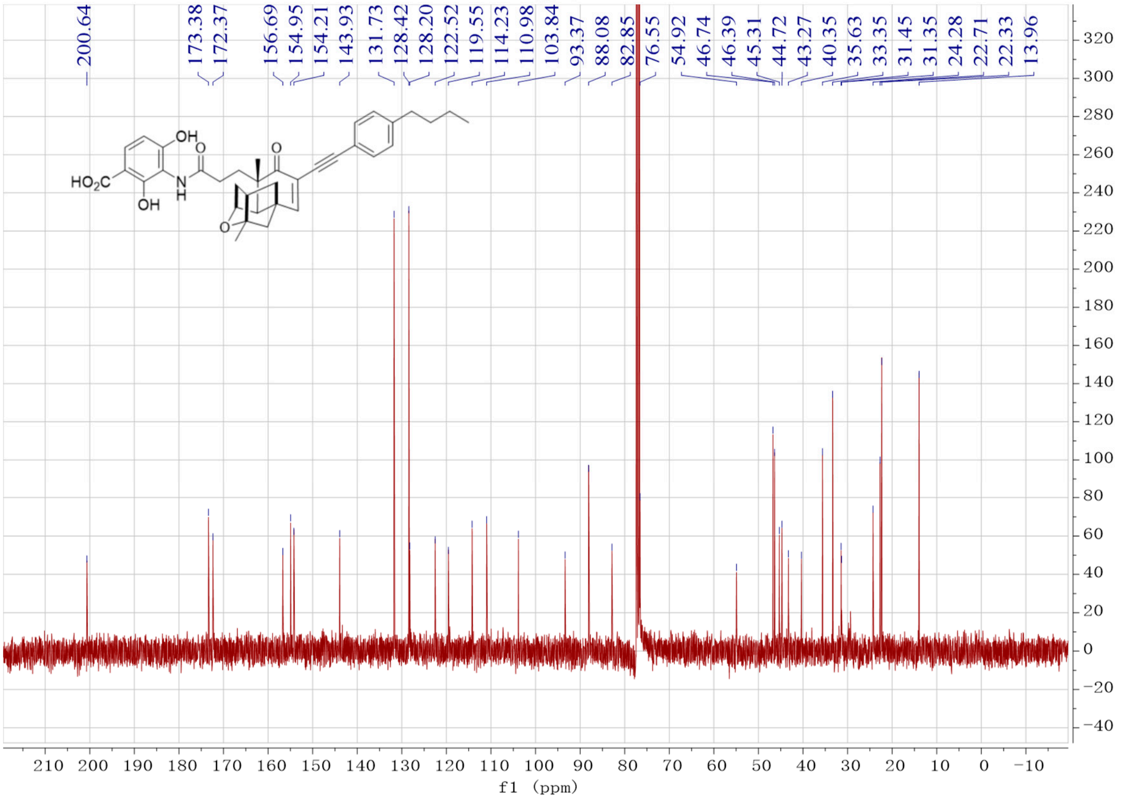

Figure S24. ^{1}H NMR (400 MHz) and ^{13}C NMR (101 MHz) spectra of **B12** in $CDCl_3$.

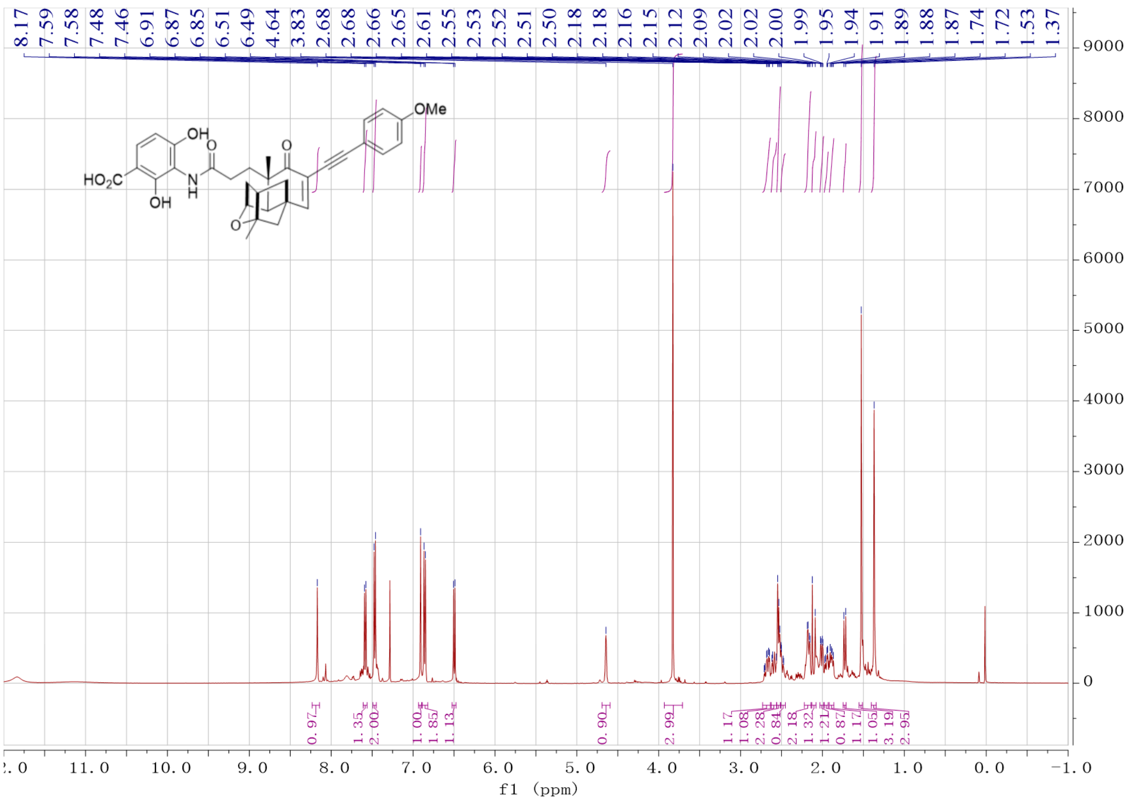

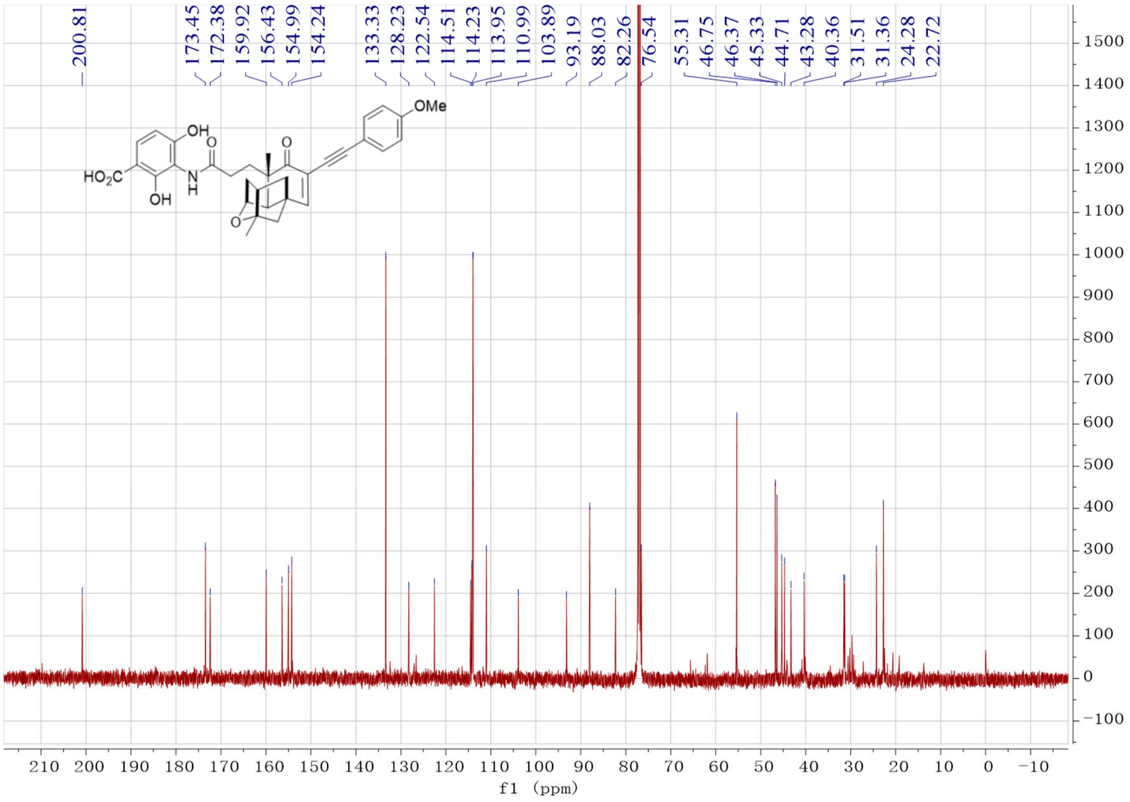

Figure S25. ^{1}H NMR (500 MHz) and ^{13}C NMR (126 MHz) spectra of **B13** in $CDCl_3$.

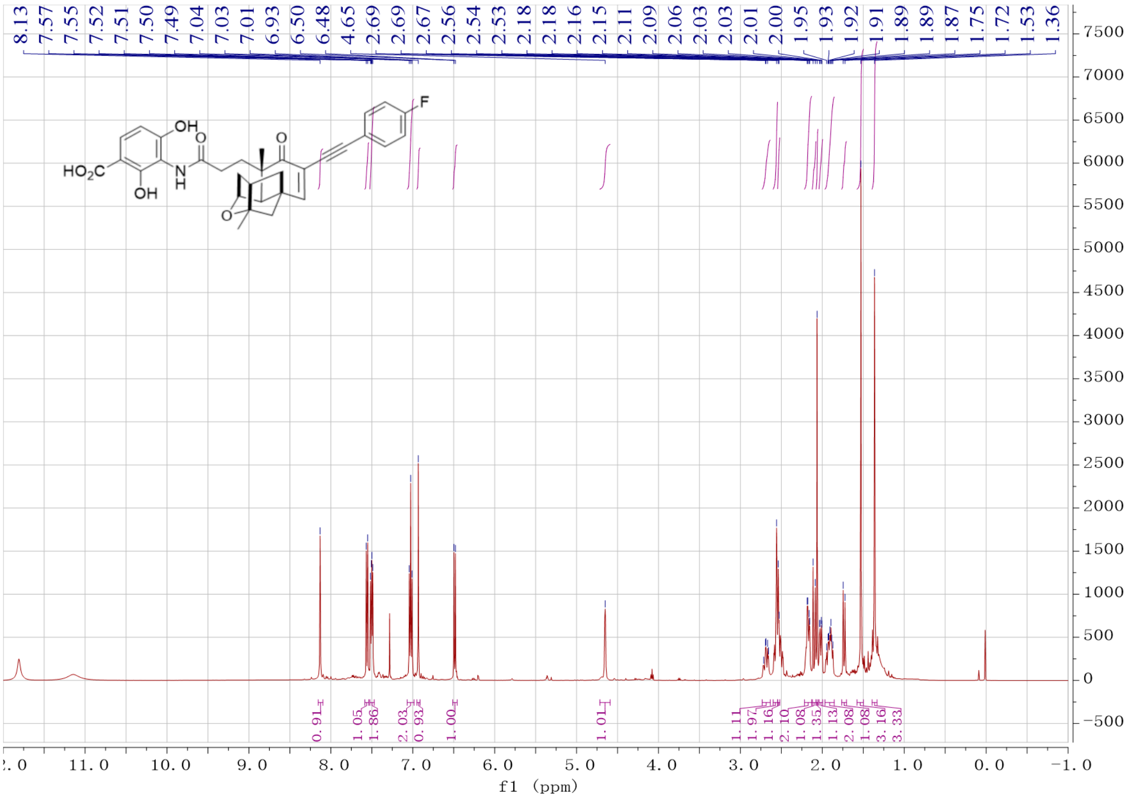

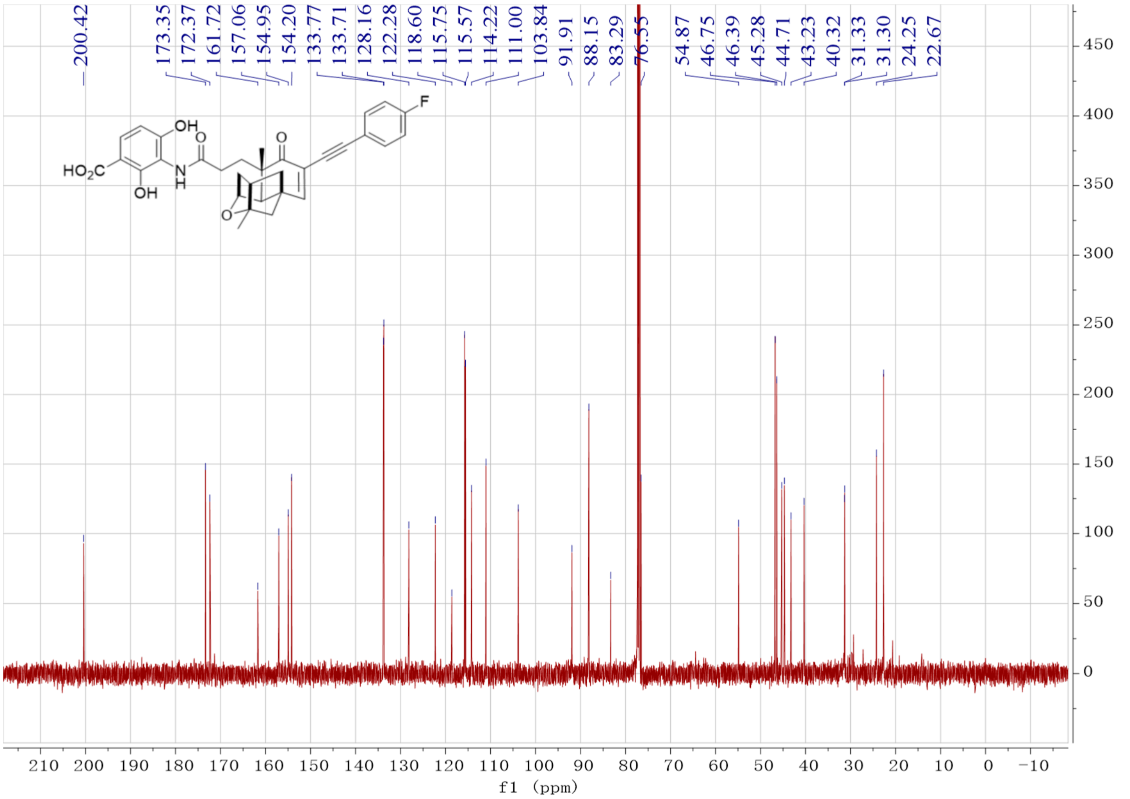

Figure S26. ^{1}H NMR (500 MHz) and ^{13}C NMR (126 MHz) spectra of **B14** in $CDCl_3$.

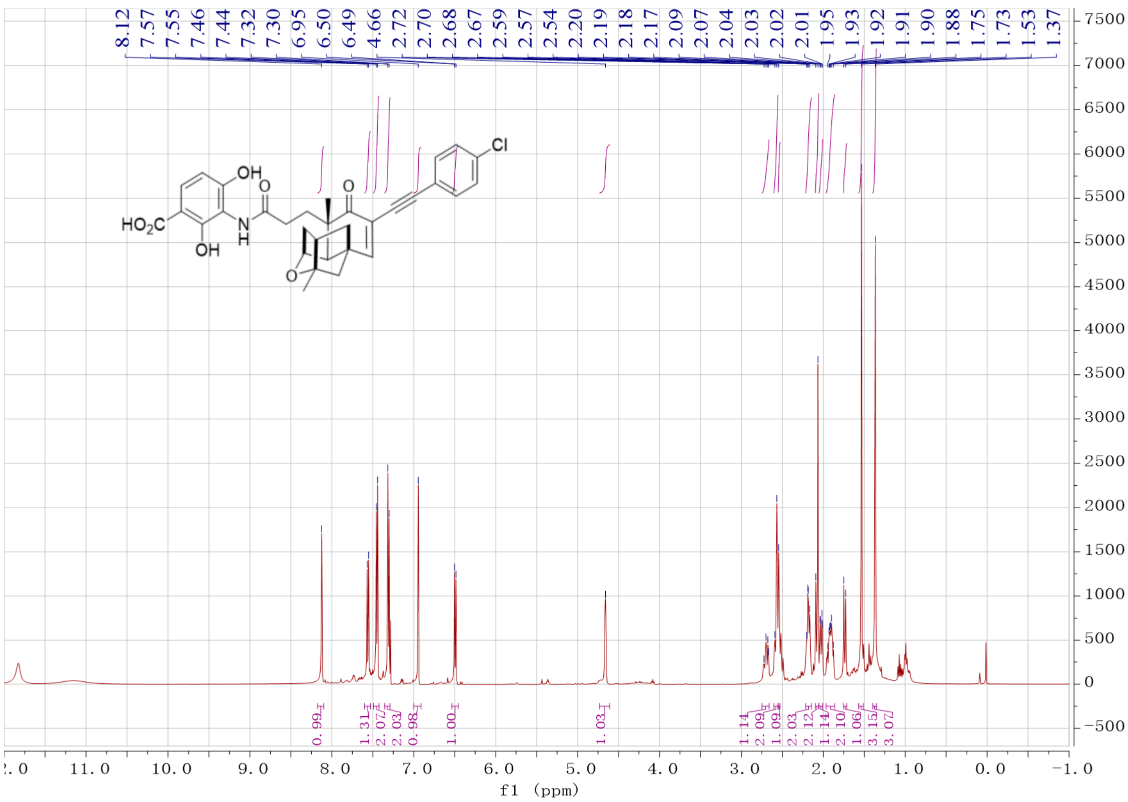

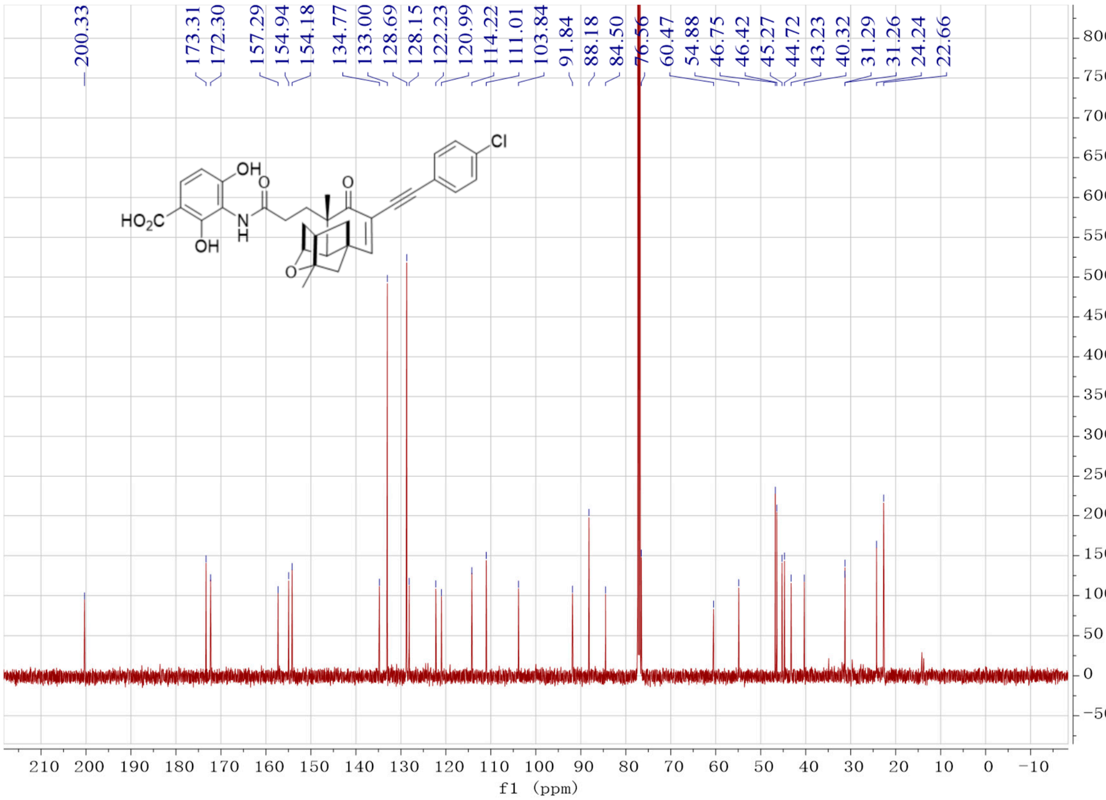

Figure S27. ^{1}H NMR (500 MHz) and ^{13}C NMR (126 MHz) spectra of **B15** in $CDCl_3$.

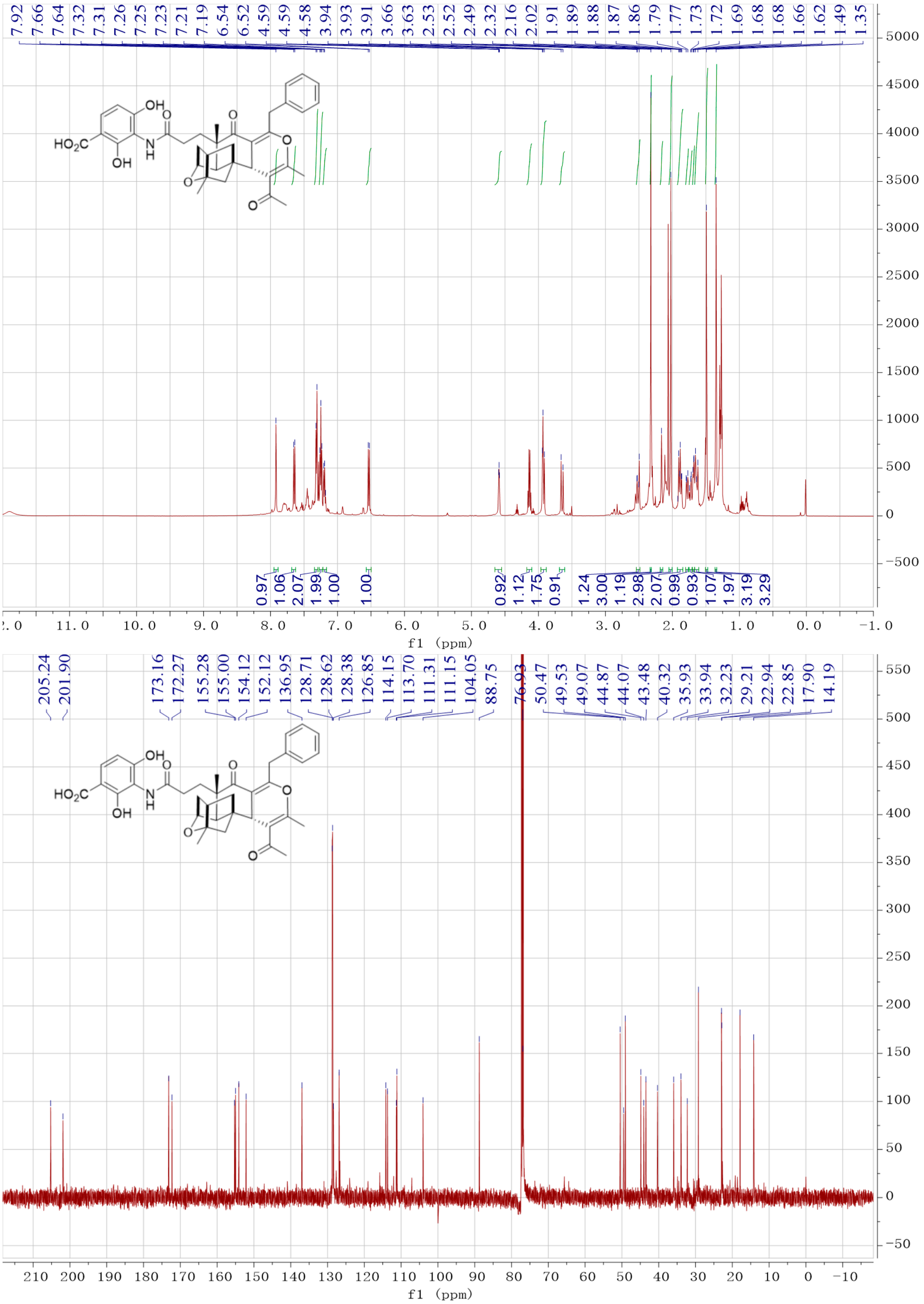

Figure S28. ^{1}H NMR (500 MHz) and ^{13}C NMR (126 MHz) spectra of **C1** in $CDCl_3$.

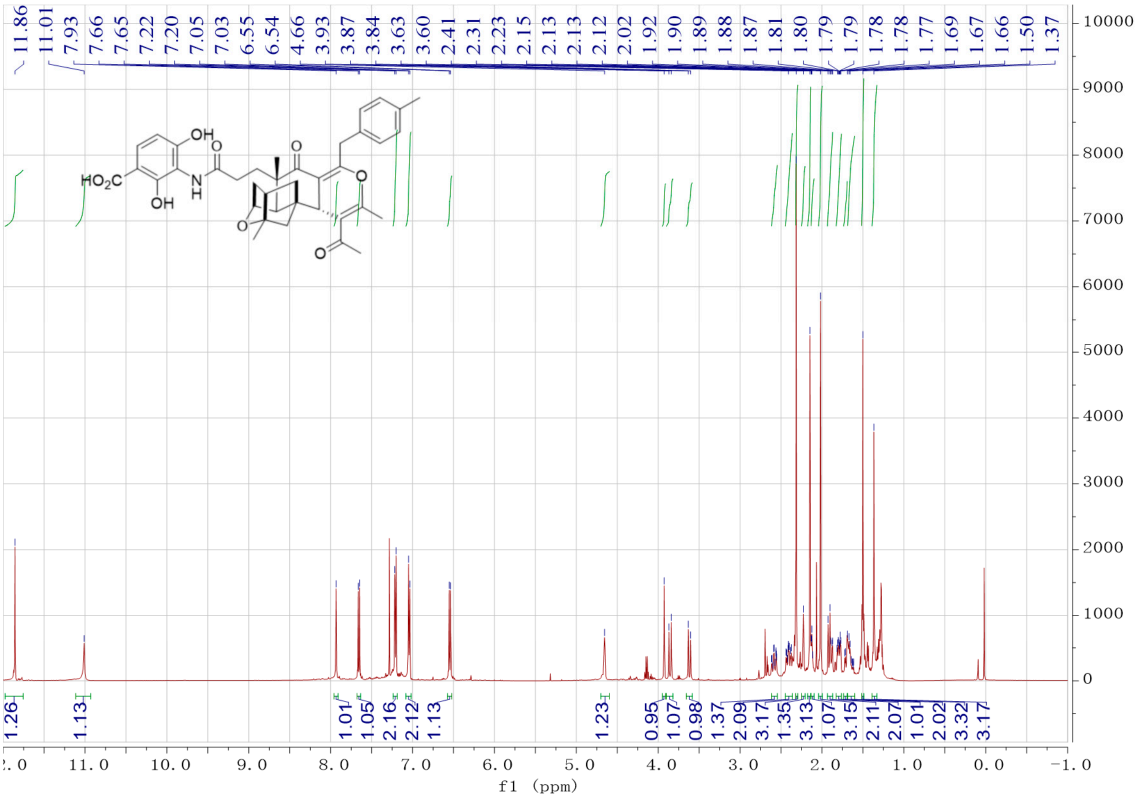

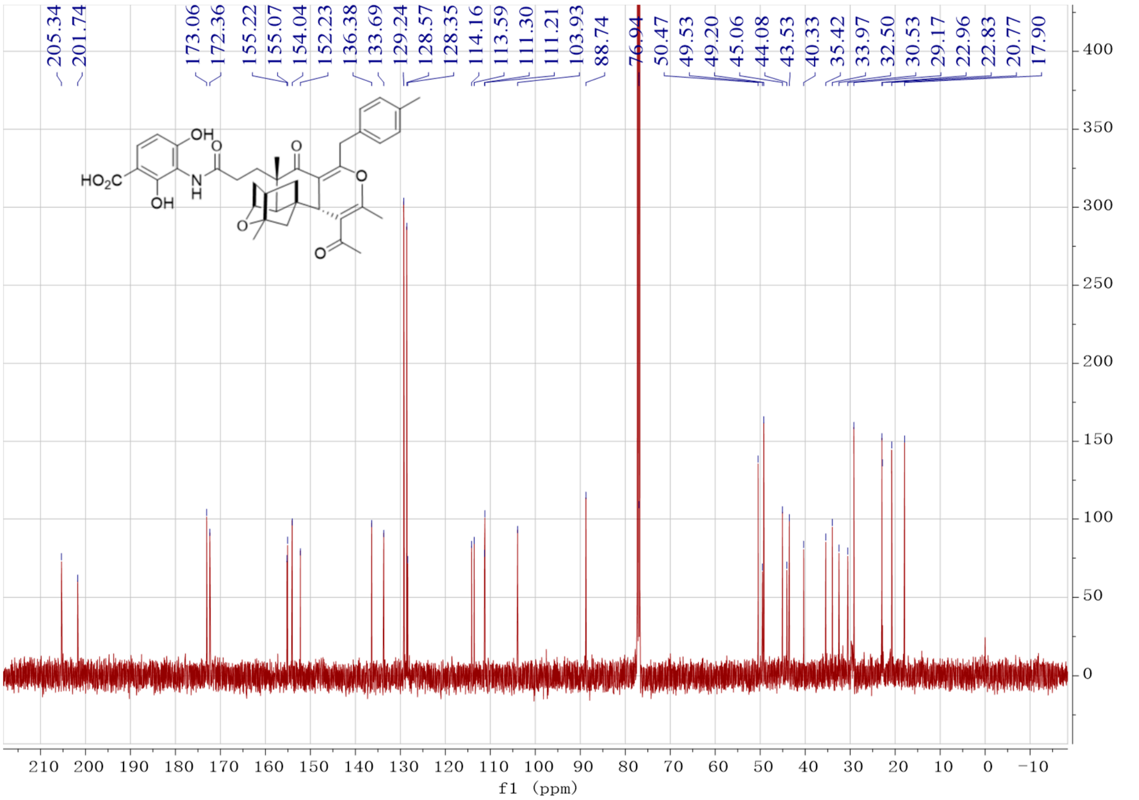

Figure S29. ^{1}H NMR (500 MHz) and ^{13}C NMR (126 MHz) spectra of **C2** in $CDCl_3$.

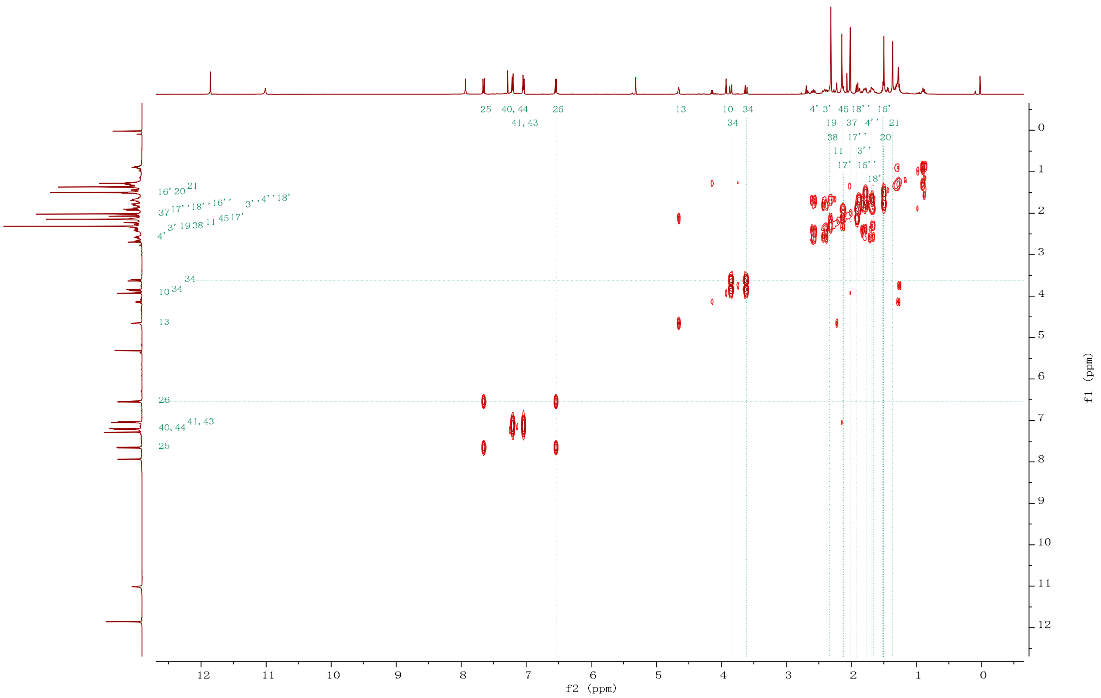

Figure S30. ^{1}H–^{1}H COSY spectra of **C2** in $CDCl_3$

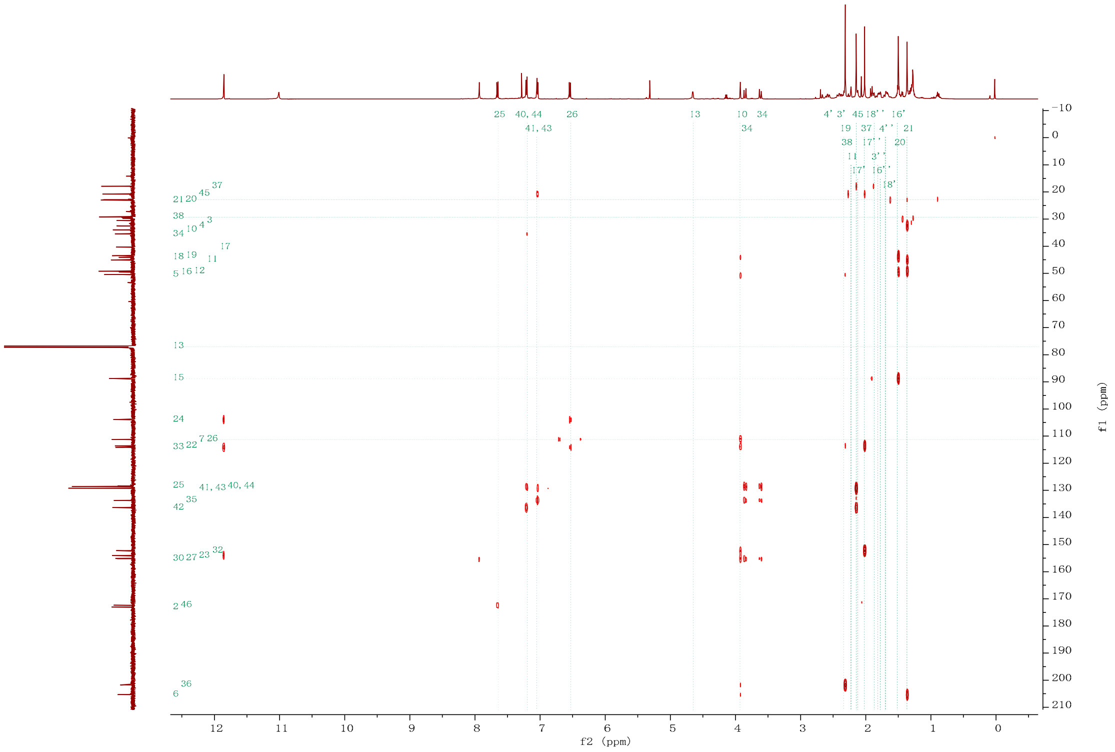

Figure S31. ^{1}H – ^{13}C HMBC spectra of **C2** in $CDCl_3$.

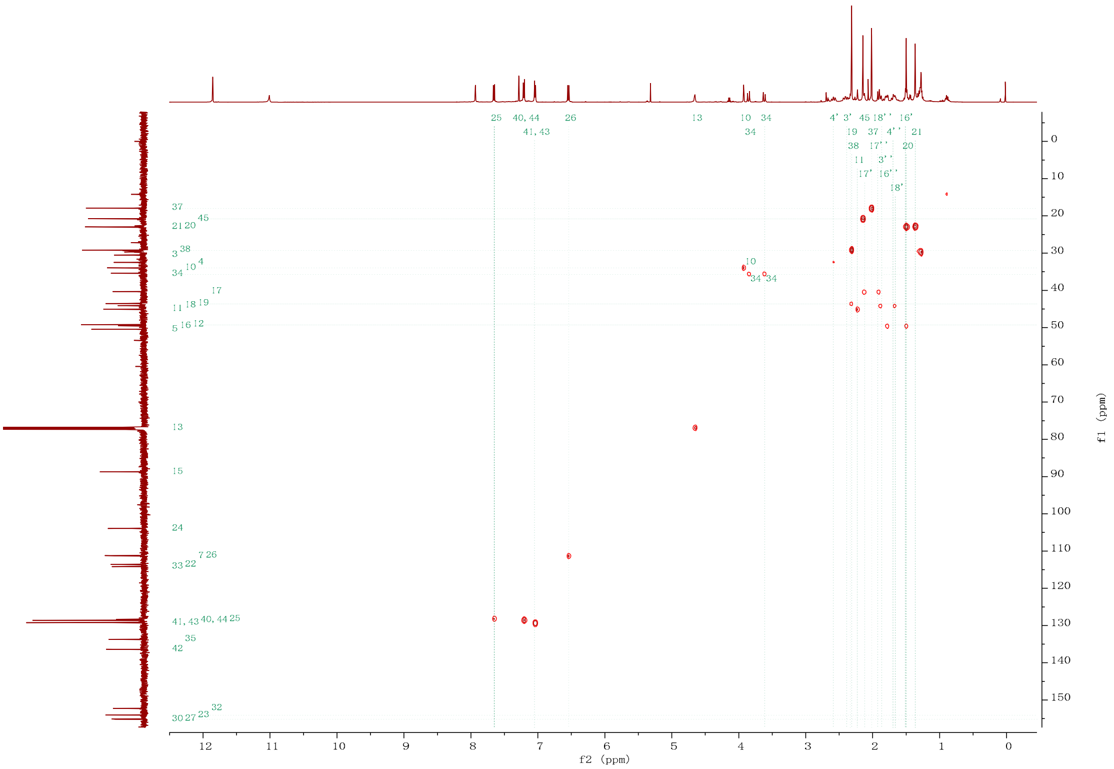

Figure S32. ^{1}H – ^{13}C HSQC spectra of **C2** in $CDCl_3$.

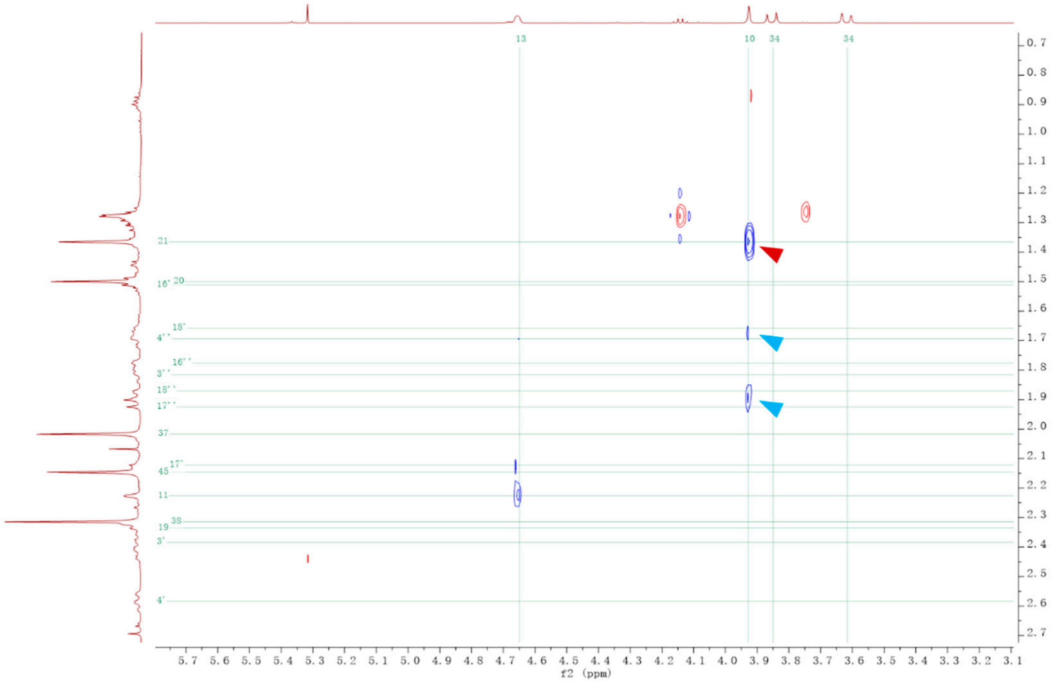

Figure S33. ^{1}H – ^{1}H ROESY spectra of **C2** in $CDCl_3$.

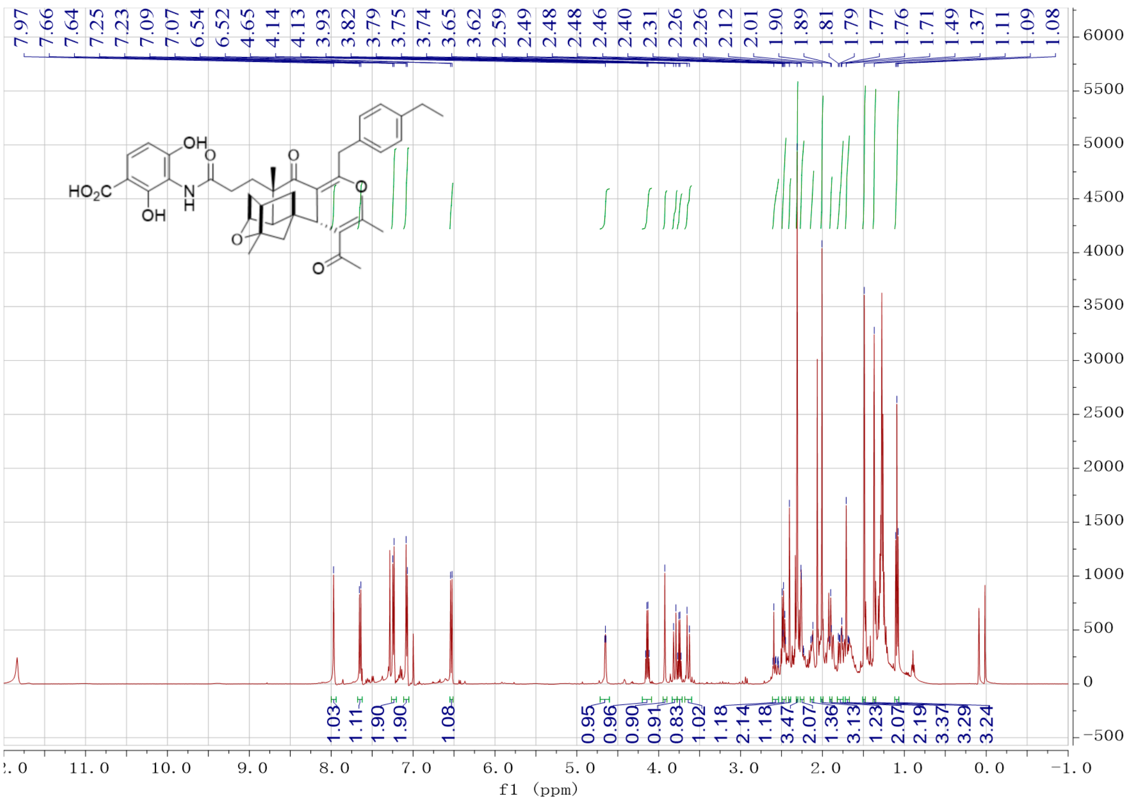

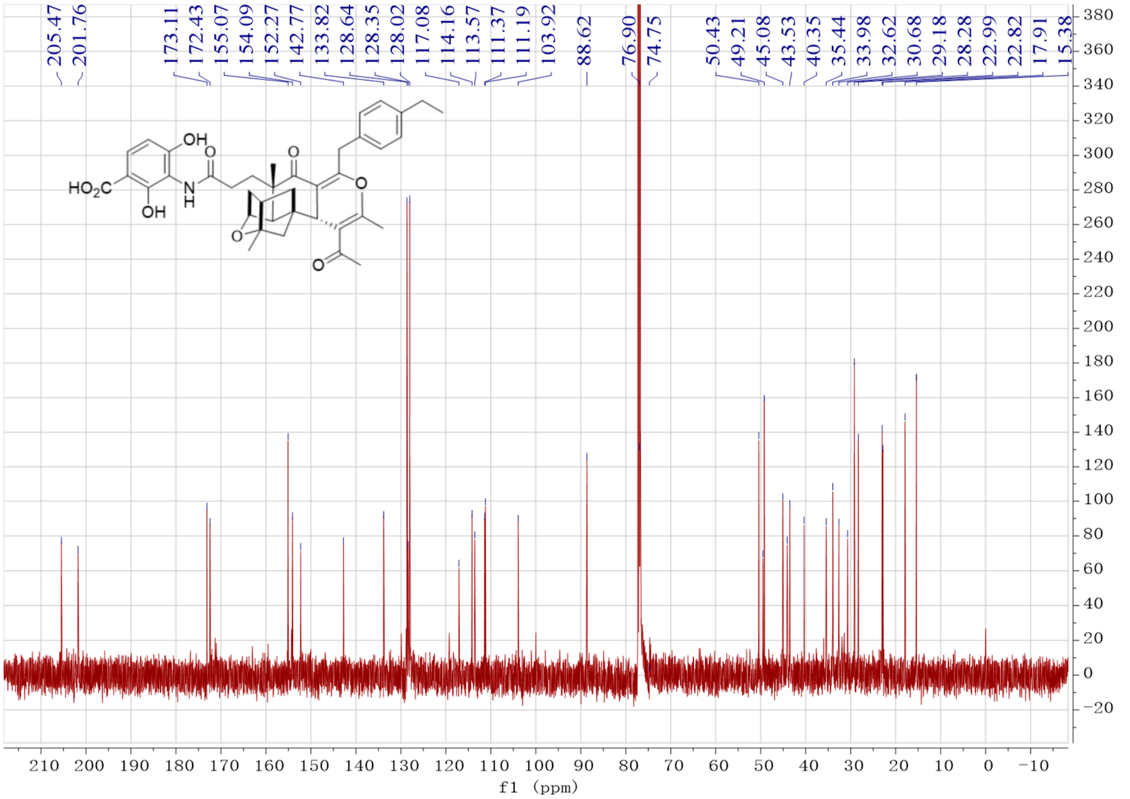

Figure S34. ^{1}H NMR (500 MHz) and ^{13}C NMR (126 MHz) spectra of **C3** in $CDCl_3$.

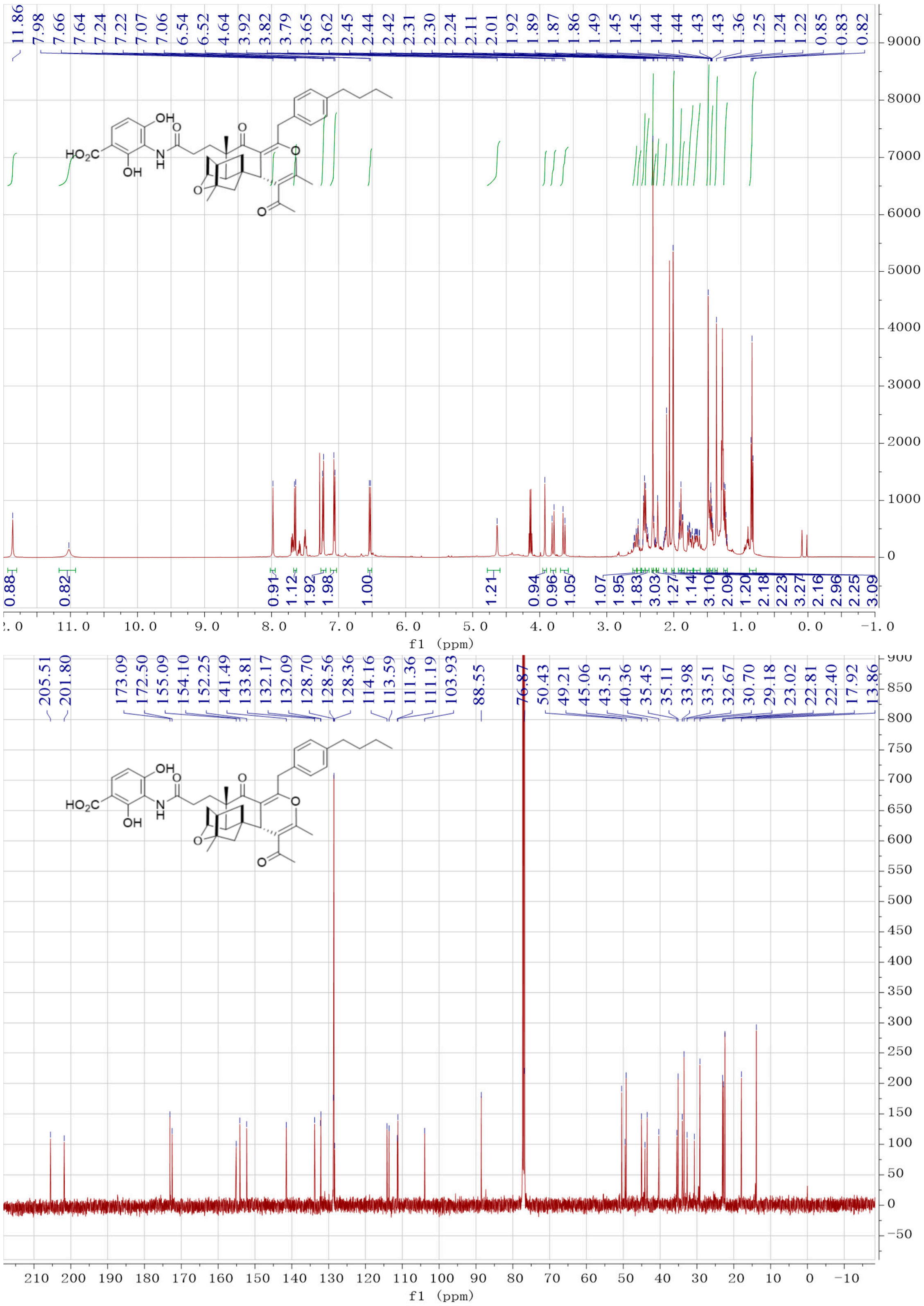

Figure S35. ^{1}H NMR (500 MHz) and ^{13}C NMR (126 MHz) spectra of **C4** in $CDCl_3$.

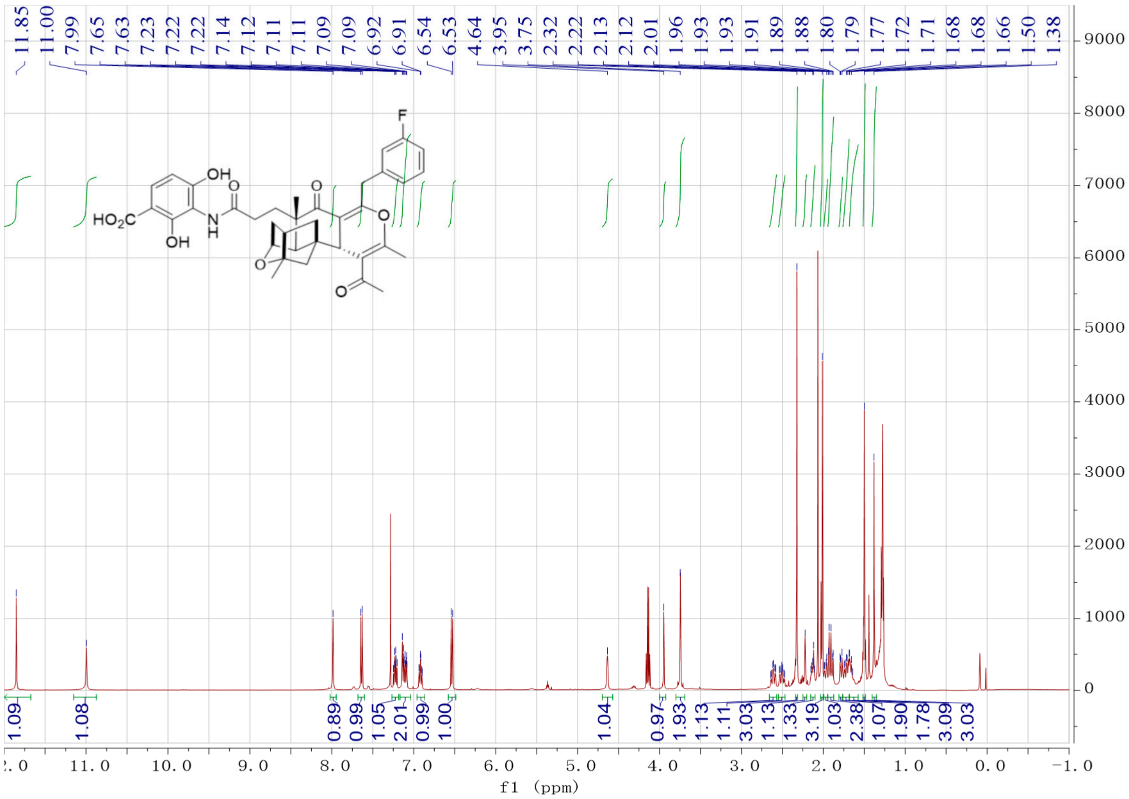

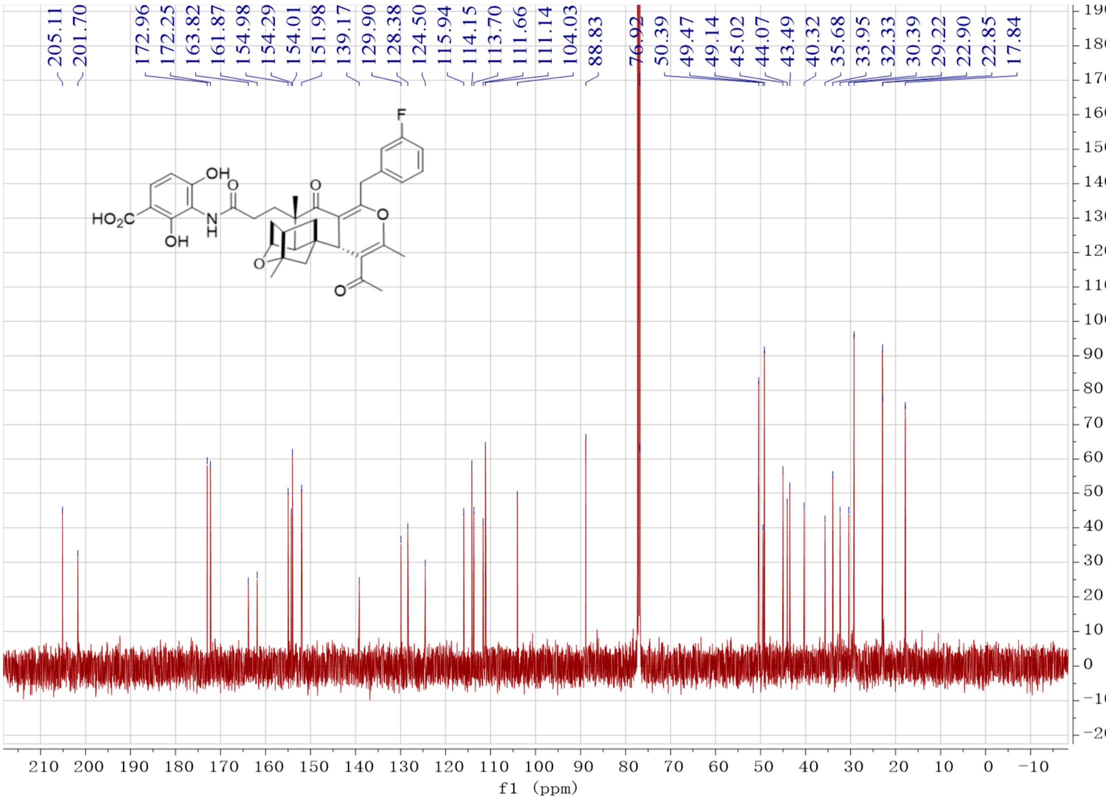

Figure S36. ^{1}H NMR (500 MHz) and ^{13}C NMR (126 MHz) spectra of **C5** in $CDCl_3$.

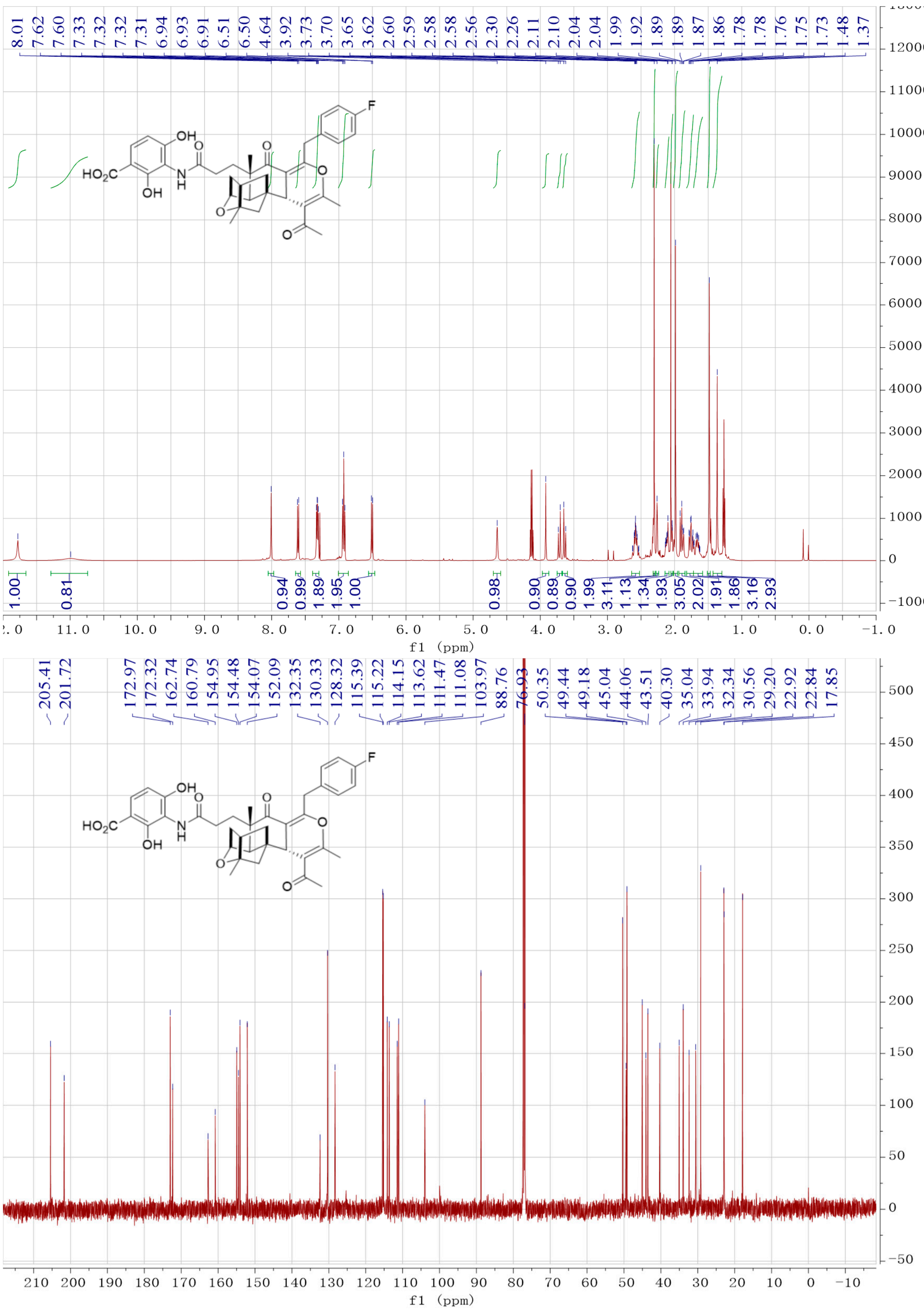

Figure S37. ^{1}H NMR (500 MHz) and ^{13}C NMR (126 MHz) spectra of **C6** in $CDCl_3$.

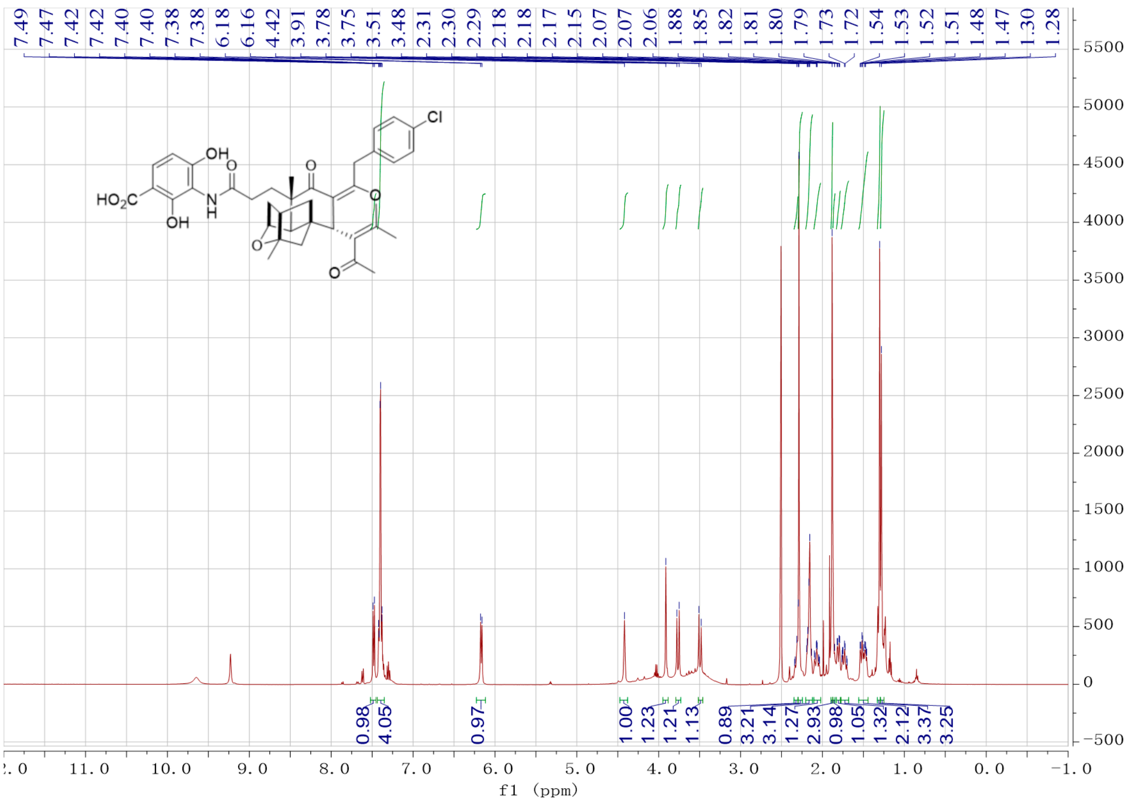

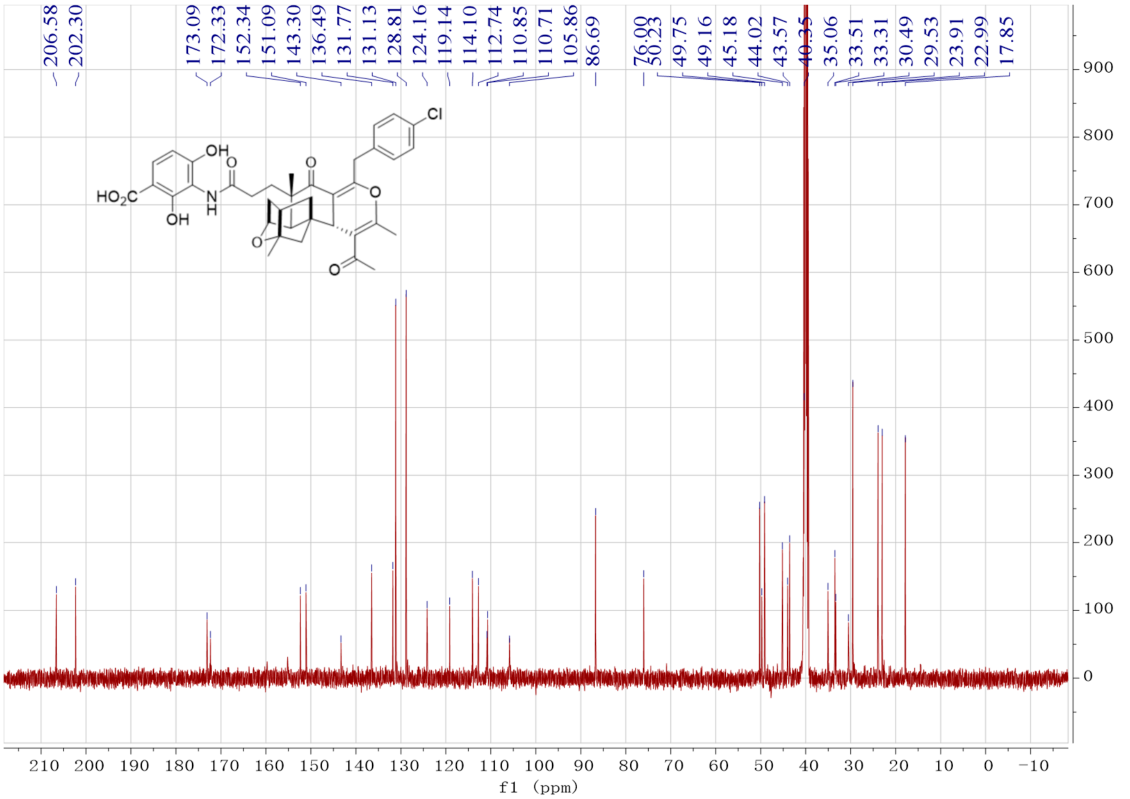

Figure S38. ^{1}H NMR (500 MHz) and ^{13}C NMR (126 MHz) spectra of **C7** in DMSO-d_6.

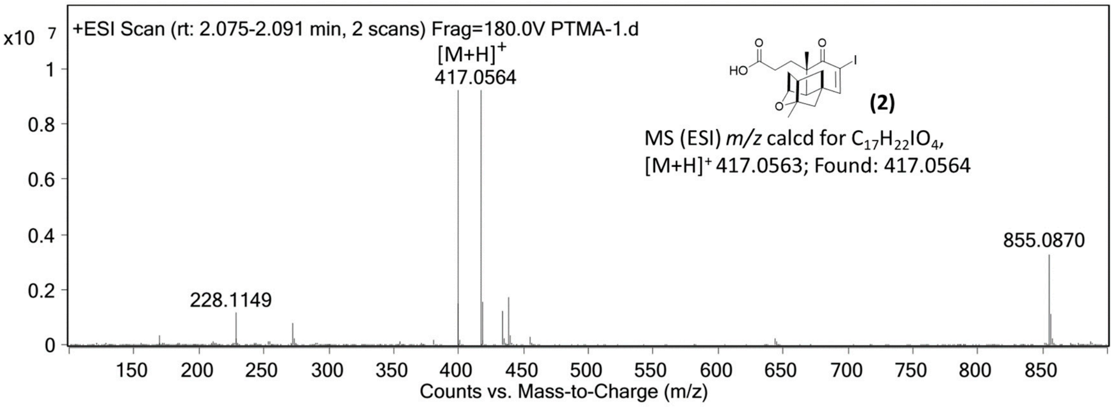

Figure S39. HRMS spectrum of **2**.

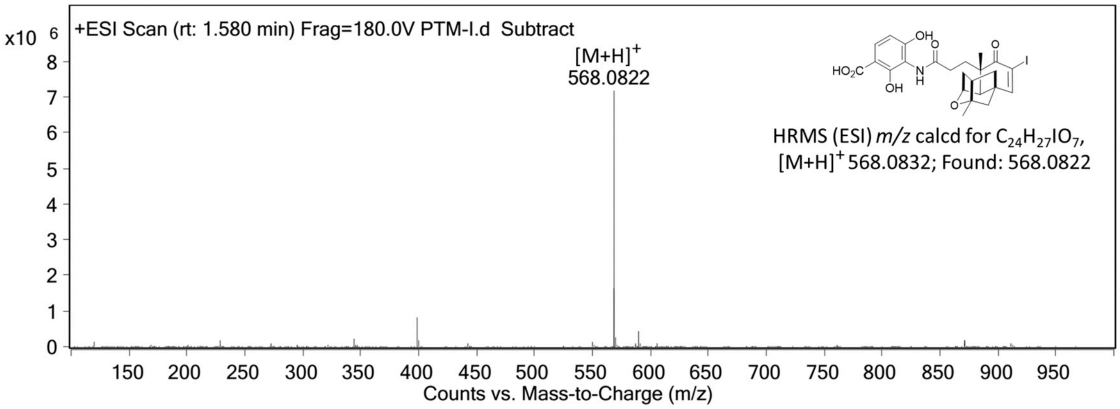

Figure S40. HRMS spectrum of **4**.

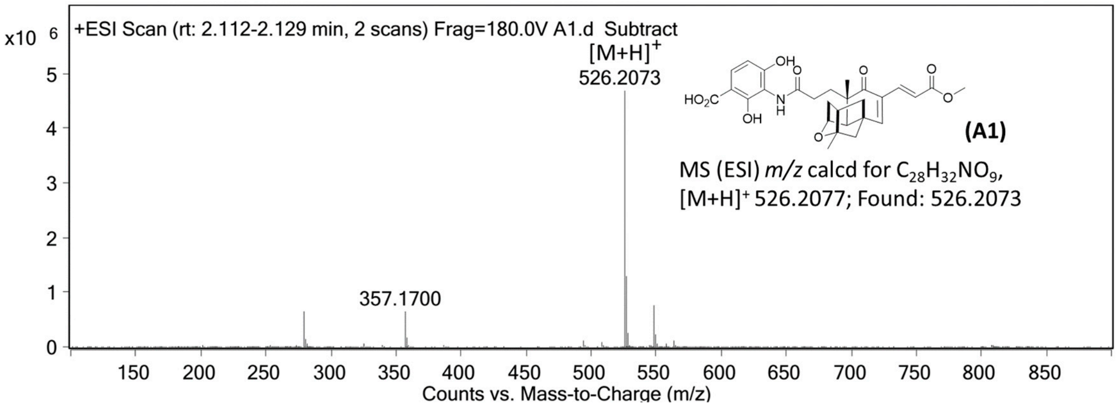
x10 6
+ESI Scan (rt: 2.112-2.129 min, 2 scans) Frag=180.0V A1.d Subtract
[M+H]+
526.2073
357.1700
(A1)
MS (ESI) m/z calcd for C28H32NO9,
[M+H]+ 526.2077; Found: 526.2073
Counts vs. Mass-to-Charge (m/z)

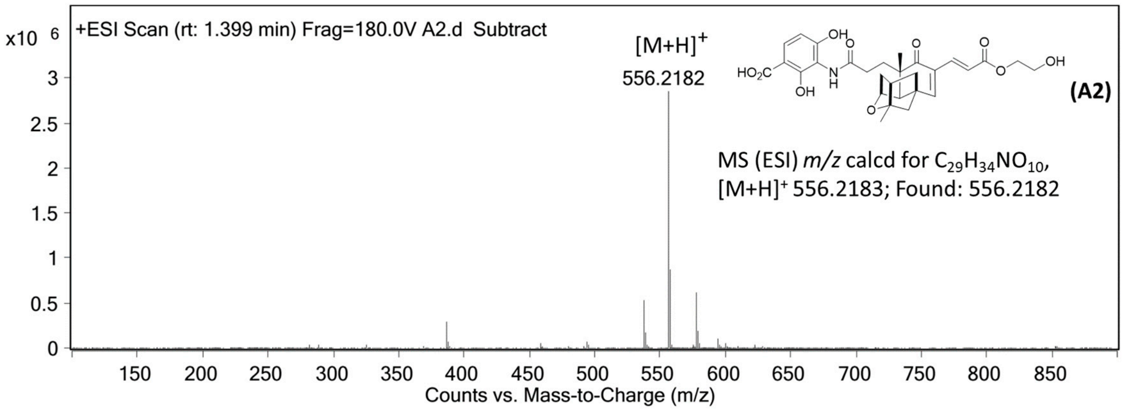
x10 6
+ESI Scan (rt: 1.399 min) Frag=180.0V A2.d Subtract
[M+H]+
556.2182
(A2)
MS (ESI) m/z calcd for C29H34NO10,
[M+H]+ 556.2183; Found: 556.2182
Counts vs. Mass-to-Charge (m/z)

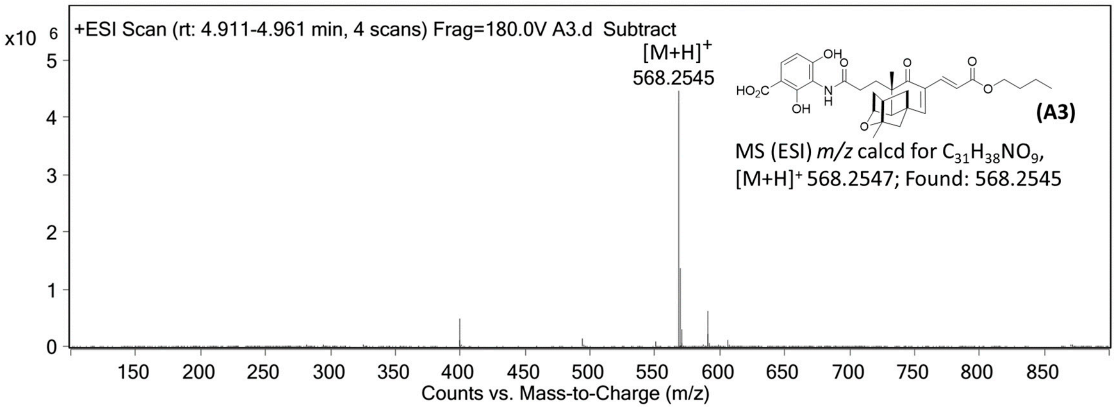
x10 6
+ESI Scan (rt: 4.911-4.961 min, 4 scans) Frag=180.0V A3.d Subtract
[M+H]+
568.2545
(A3)
MS (ESI) m/z calcd for C31H38NO9,
[M+H]+ 568.2547; Found: 568.2545
Counts vs. Mass-to-Charge (m/z)

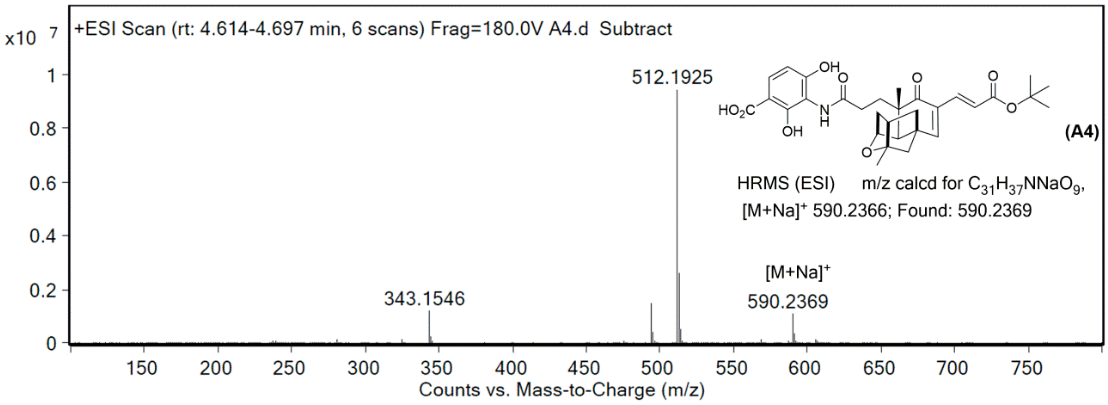
x10 7
+ESI Scan (rt: 4.614-4.697 min, 6 scans) Frag=180.0V A4.d Subtract
512.1925
(A4)
HRMS (ESI) m/z calcd for C31H37NNaO9,
[M+Na]+ 590.2366; Found: 590.2369
343.1546
[M+Na]+
590.2369
Counts vs. Mass-to-Charge (m/z)

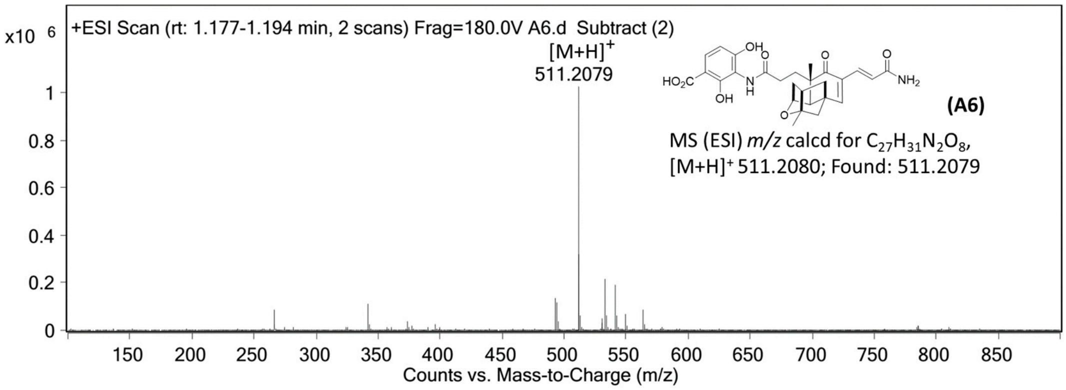
x10 6
+ESI Scan (rt: 1.177-1.194 min, 2 scans) Frag=180.0V A6.d Subtract (2)
[M+H]+
511.2079
(A6)
MS (ESI) m/z calcd for C27H31N2O8, [M+H]+ 511.2080; Found: 511.2079
Counts vs. Mass-to-Charge (m/z)

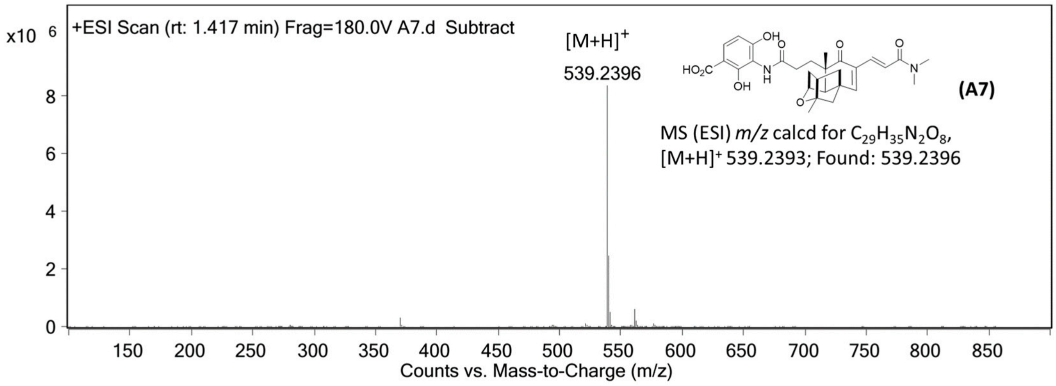
x10 6
+ESI Scan (rt: 1.417 min) Frag=180.0V A7.d Subtract
[M+H]+
539.2396
(A7)
MS (ESI) m/z calcd for C29H35N2O8, [M+H]+ 539.2393; Found: 539.2396
Counts vs. Mass-to-Charge (m/z)

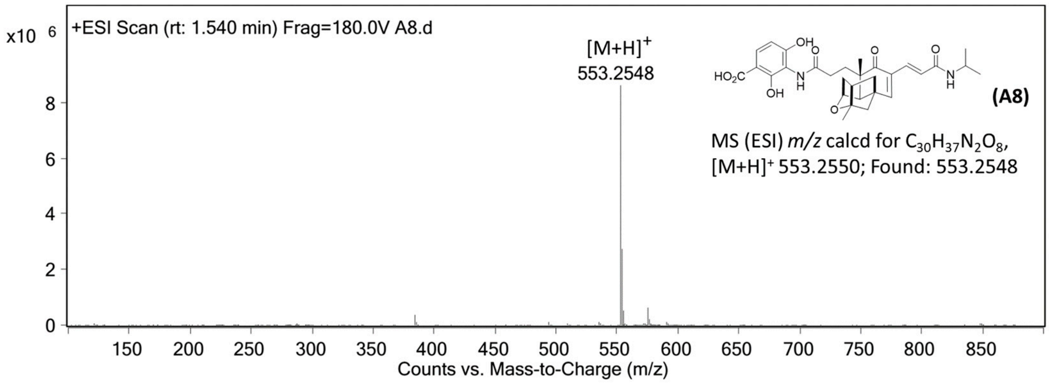
x10 6
+ESI Scan (rt: 1.540 min) Frag=180.0V A8.d
[M+H]+
553.2548
(A8)
MS (ESI) m/z calcd for C30H37N2O8, [M+H]+ 553.2550; Found: 553.2548
Counts vs. Mass-to-Charge (m/z)

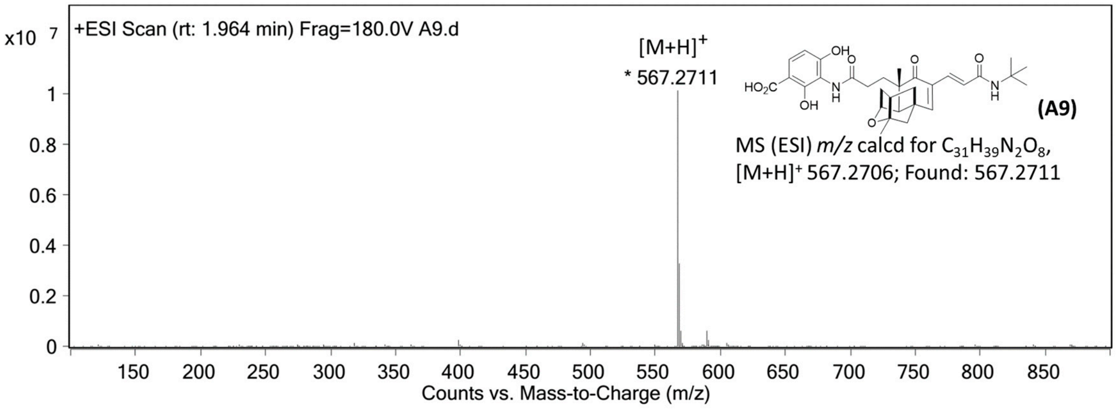
x10 7
+ESI Scan (rt: 1.964 min) Frag=180.0V A9.d
[M+H]+
* 567.2711
(A9)
MS (ESI) m/z calcd for C31H39N2O8, [M+H]+ 567.2706; Found: 567.2711
Counts vs. Mass-to-Charge (m/z)

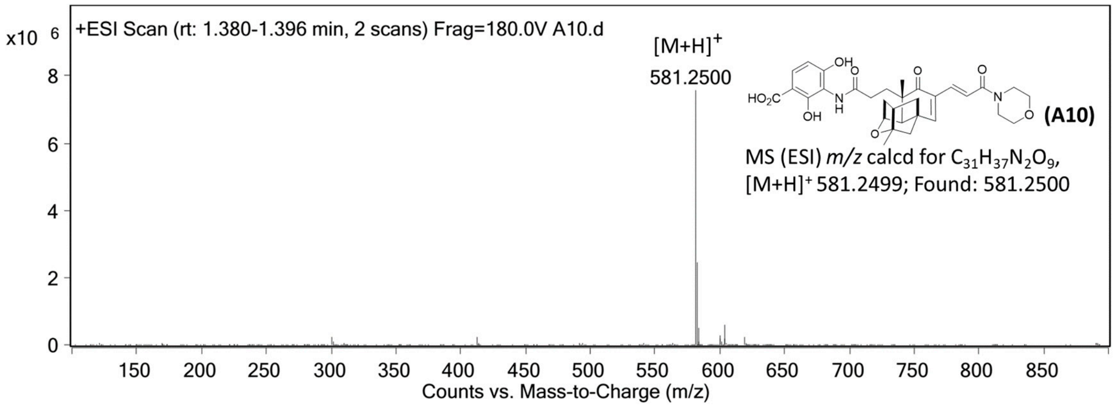

Figure S41. HRMS spectra of **A1 − A10**.

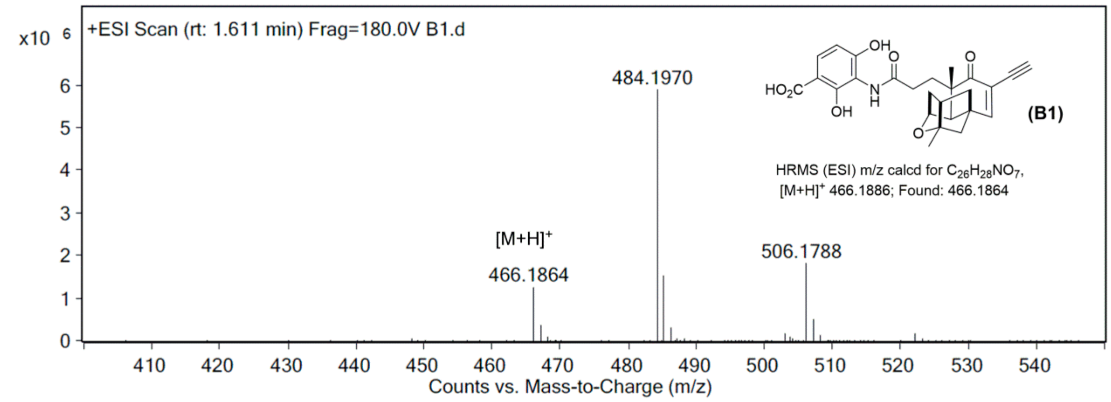
x10 6
+ESI Scan (rt: 1.611 min) Frag=180.0V B1.d
484.1970
[M+H]+
466.1864
506.1788
(B1)
HO2C
OH
OH
HRMS (ESI) m/z calcd for C26H28NO7,
[M+H]+ 466.1886; Found: 466.1864
Counts vs. Mass-to-Charge (m/z)

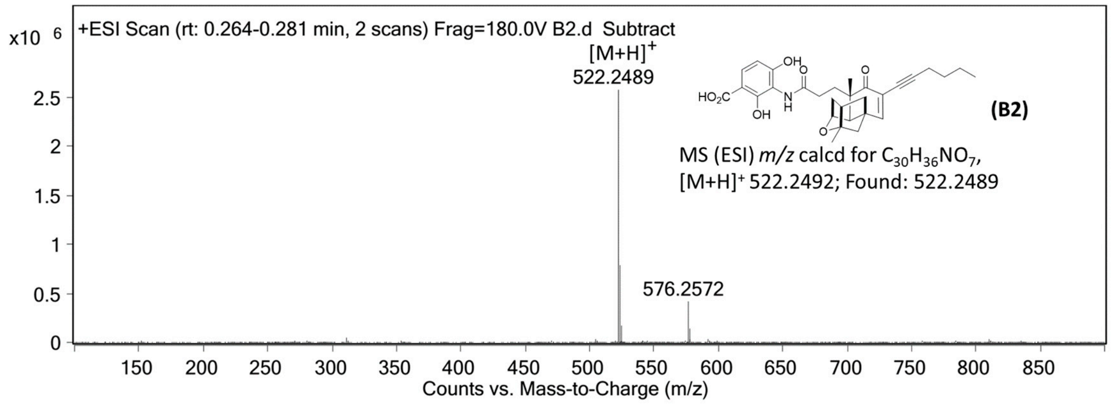
x10 6
+ESI Scan (rt: 0.264-0.281 min, 2 scans) Frag=180.0V B2.d Subtract
[M+H]+
522.2489
576.2572
(B2)
MS (ESI) m/z calcd for C30H36NO7,
[M+H]+ 522.2492; Found: 522.2489
Counts vs. Mass-to-Charge (m/z)

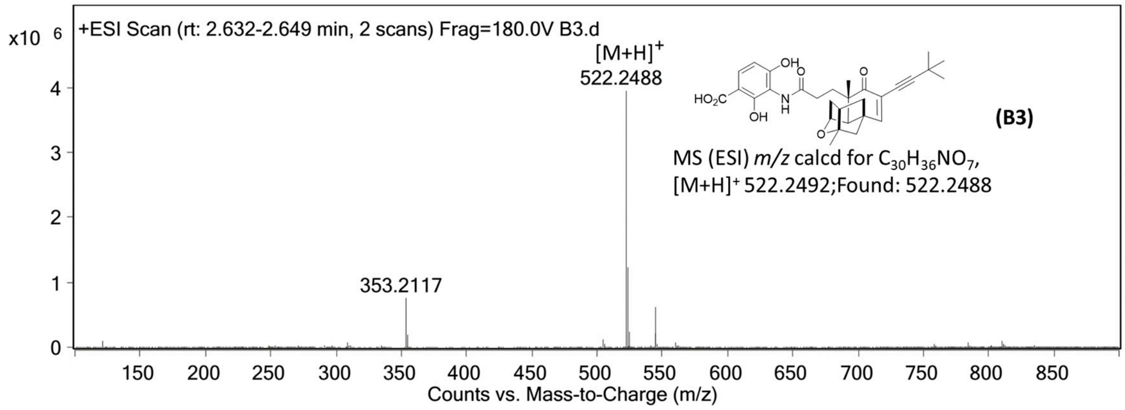
x10 6
+ESI Scan (rt: 2.632-2.649 min, 2 scans) Frag=180.0V B3.d
[M+H]+
522.2488
353.2117
(B3)
MS (ESI) m/z calcd for C30H36NO7,
[M+H]+ 522.2492;Found: 522.2488
Counts vs. Mass-to-Charge (m/z)

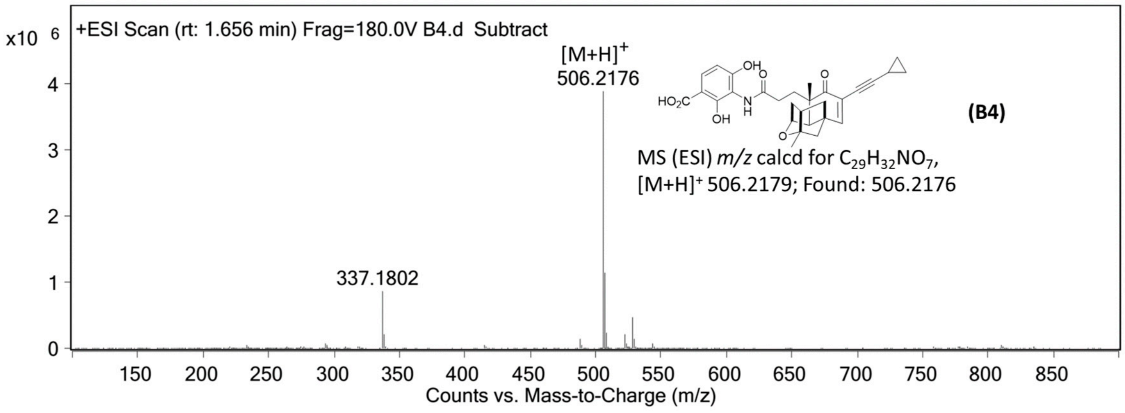
x10 6
+ESI Scan (rt: 1.656 min) Frag=180.0V B4.d Subtract
[M+H]+
506.2176
337.1802
(B4)
MS (ESI) m/z calcd for C29H32NO7,
[M+H]+ 506.2179; Found: 506.2176
Counts vs. Mass-to-Charge (m/z)

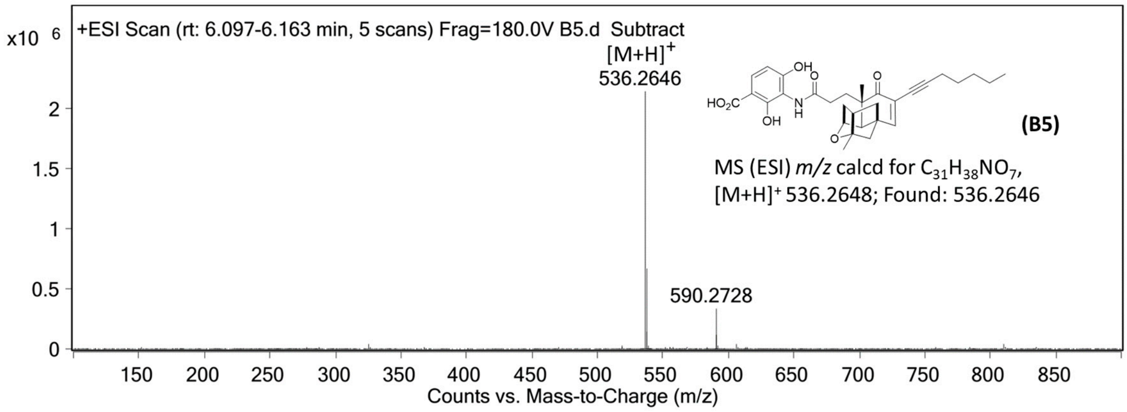
x10 6
+ESI Scan (rt: 6.097-6.163 min, 5 scans) Frag=180.0V B5.d Subtract
[M+H]+
536.2646
590.2728
(B5)
MS (ESI) m/z calcd for $C_{31}H_{38}NO_7$, [M+H]+ 536.2648; Found: 536.2646
Counts vs. Mass-to-Charge (m/z)

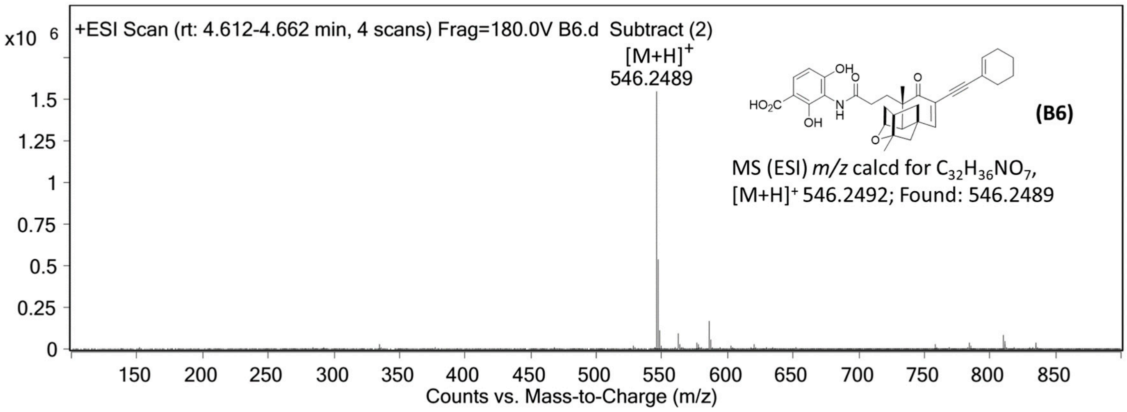
x10 6
+ESI Scan (rt: 4.612-4.662 min, 4 scans) Frag=180.0V B6.d Subtract (2)
[M+H]+
546.2489
(B6)
MS (ESI) m/z calcd for $C_{32}H_{36}NO_7$, [M+H]+ 546.2492; Found: 546.2489
Counts vs. Mass-to-Charge (m/z)

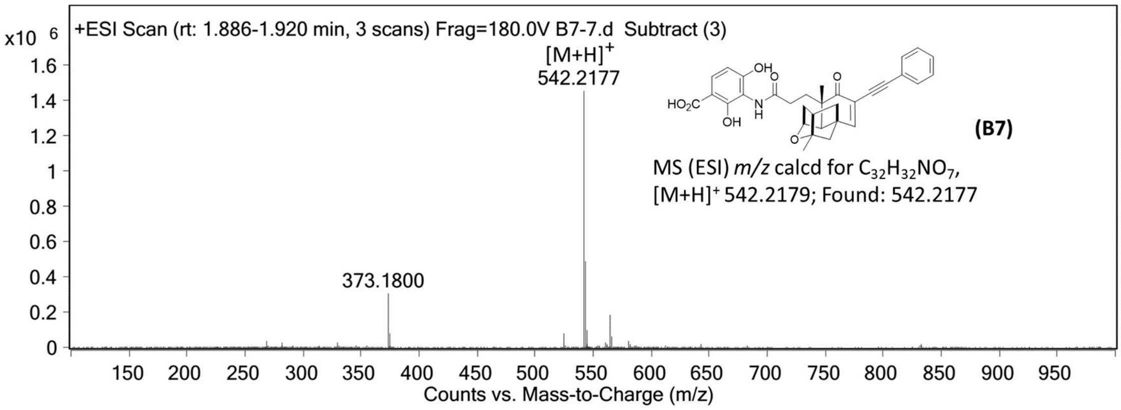
x10 6
+ESI Scan (rt: 1.886-1.920 min, 3 scans) Frag=180.0V B7-7.d Subtract (3)
[M+H]+
542.2177
373.1800
(B7)
MS (ESI) m/z calcd for $C_{32}H_{32}NO_7$, [M+H]+ 542.2179; Found: 542.2177
Counts vs. Mass-to-Charge (m/z)

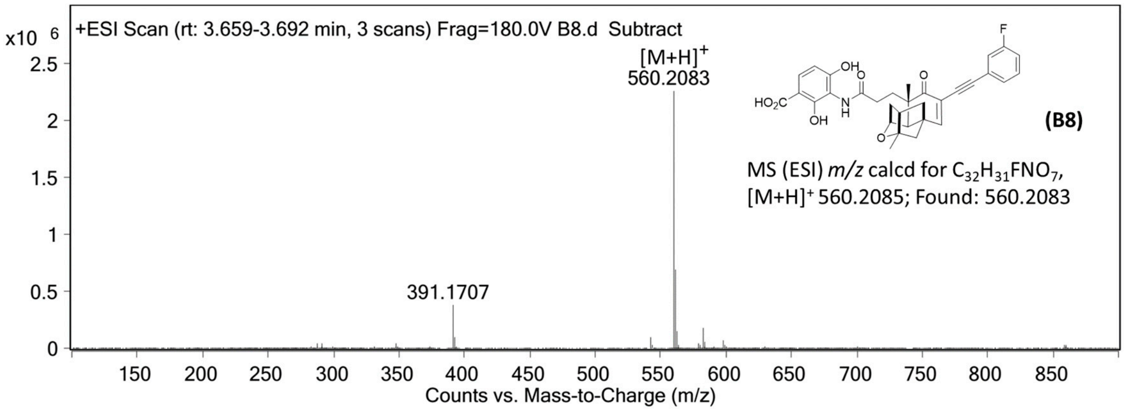
x10 6
+ESI Scan (rt: 3.659-3.692 min, 3 scans) Frag=180.0V B8.d Subtract
[M+H]+
560.2083
391.1707
(B8)
MS (ESI) m/z calcd for $C_{32}H_{31}FNO_7$, [M+H]+ 560.2085; Found: 560.2083
Counts vs. Mass-to-Charge (m/z)

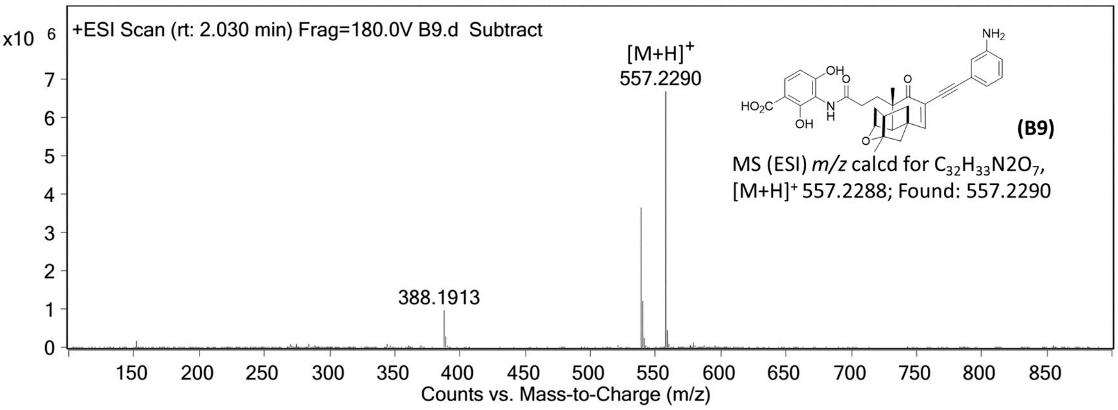
x10 6
+ESI Scan (rt: 2.030 min) Frag=180.0V B9.d Subtract
[M+H]+
557.2290
388.1913
Counts vs. Mass-to-Charge (m/z)
(B9)
MS (ESI) m/z calcd for $C_{32}H_{33}N2O_7$,
$[M+H]^+$ 557.2288; Found: 557.2290

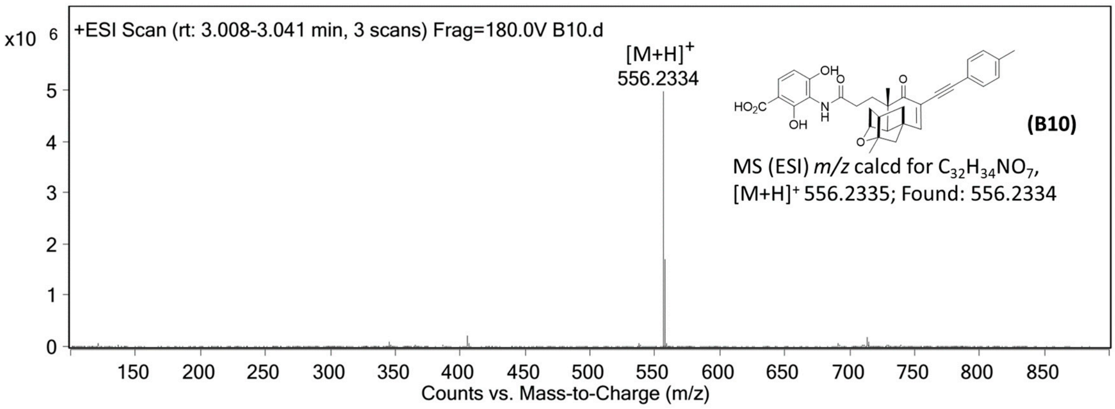
x10 6
+ESI Scan (rt: 3.008-3.041 min, 3 scans) Frag=180.0V B10.d
[M+H]+
556.2334
Counts vs. Mass-to-Charge (m/z)
(B10)
MS (ESI) m/z calcd for $C_{32}H_{34}NO_7$,
$[M+H]^+$ 556.2335; Found: 556.2334

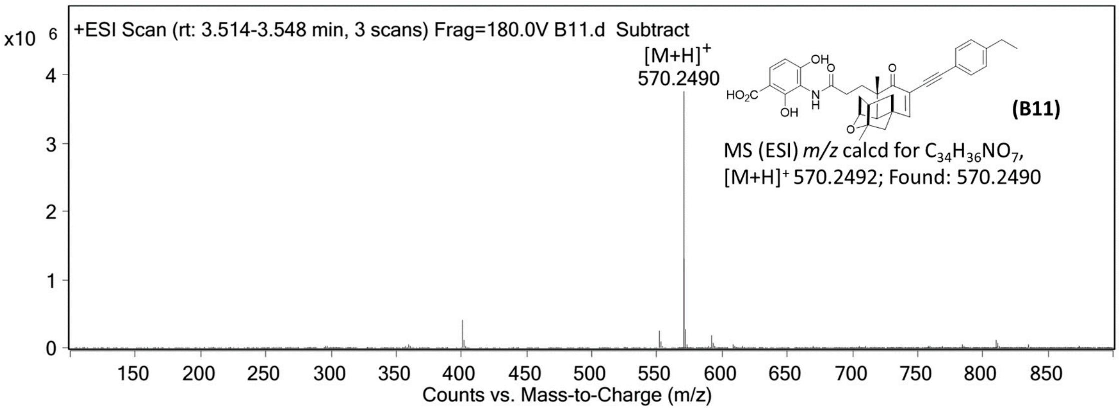
x10 6
+ESI Scan (rt: 3.514-3.548 min, 3 scans) Frag=180.0V B11.d Subtract
[M+H]+
570.2490
Counts vs. Mass-to-Charge (m/z)
(B11)
MS (ESI) m/z calcd for $C_{34}H_{36}NO_7$,
$[M+H]^+$ 570.2492; Found: 570.2490

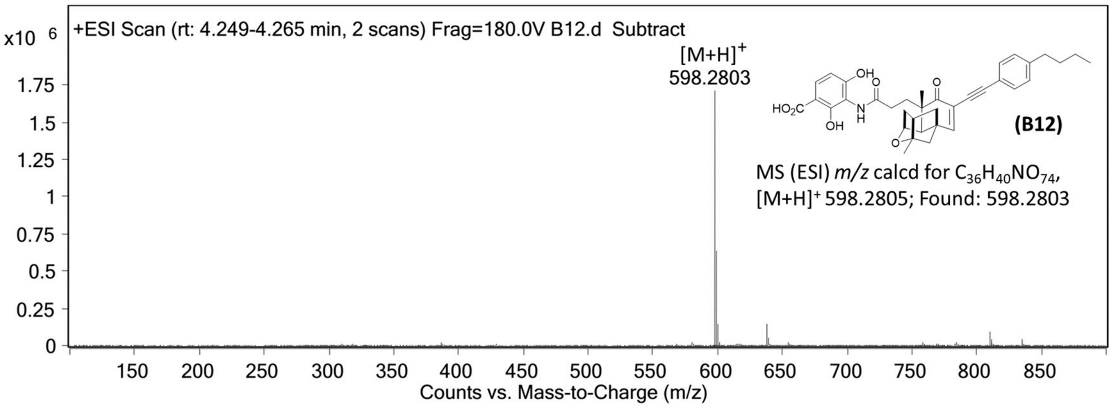
x10 6
+ESI Scan (rt: 4.249-4.265 min, 2 scans) Frag=180.0V B12.d Subtract
[M+H]+
598.2803
Counts vs. Mass-to-Charge (m/z)
(B12)
MS (ESI) m/z calcd for $C_{36}H_{40}NO_{74}$,
$[M+H]^+$ 598.2805; Found: 598.2803

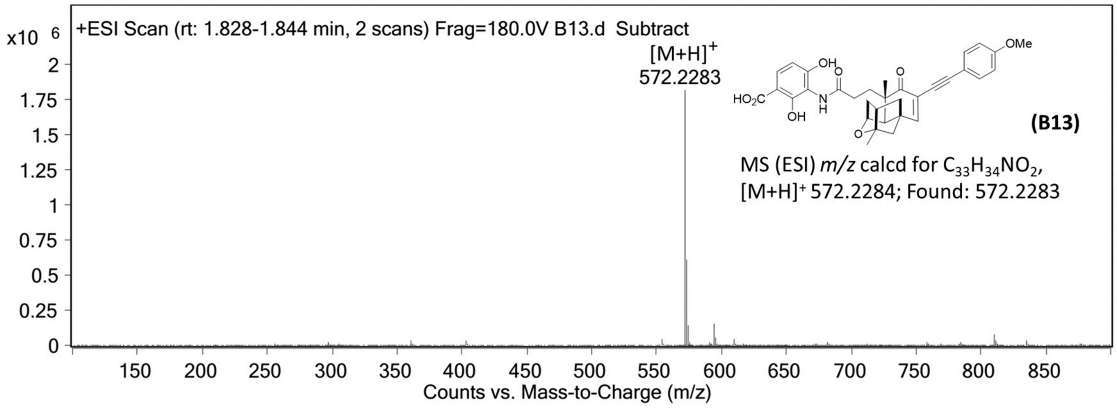

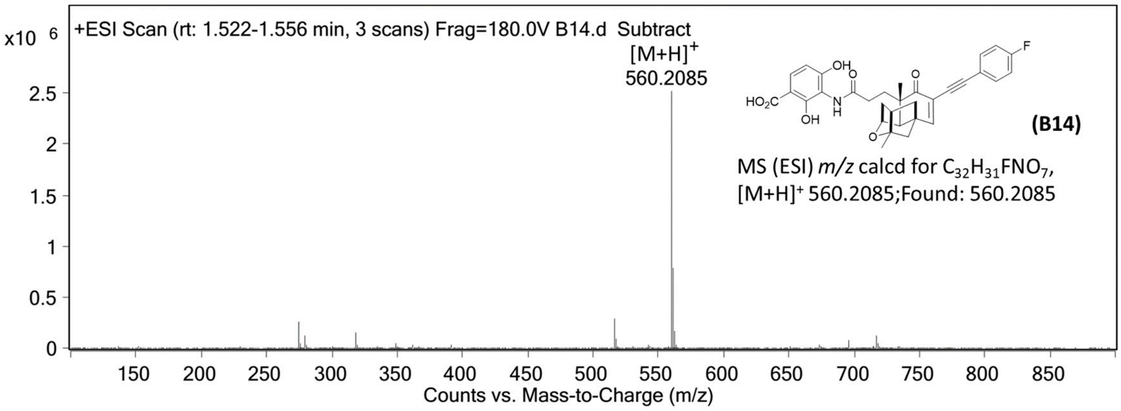

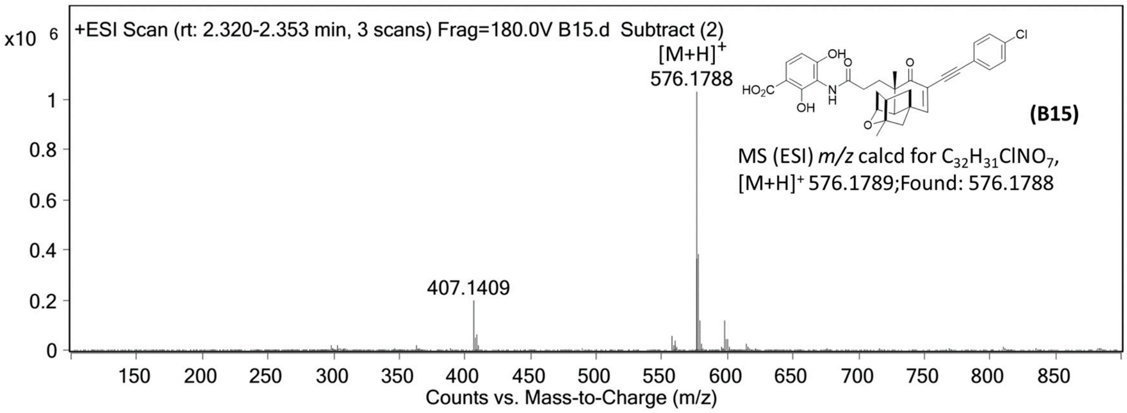

Figure S42. HRMS spectra of **B1 − B15**.

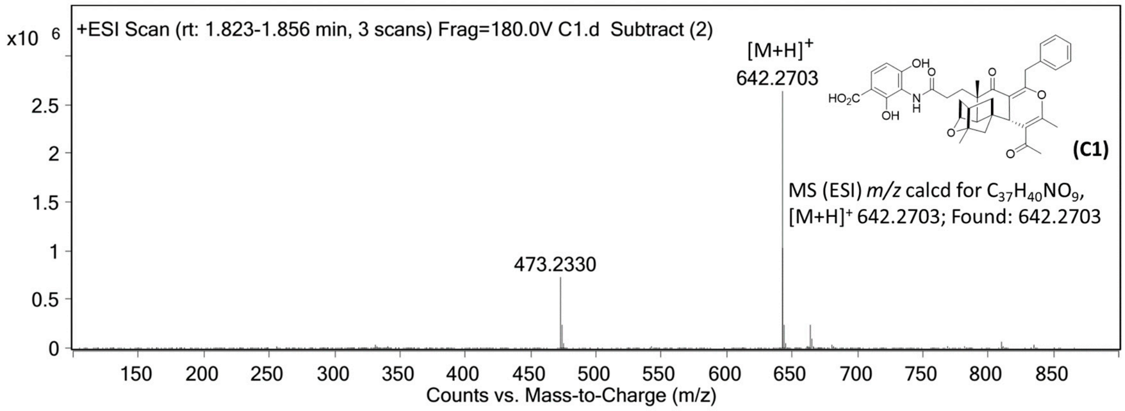
+ESI Scan (rt: 1.823-1.856 min, 3 scans) Frag=180.0V C1.d Subtract (2)
x10 6
[M+H]+
642.2703
473.2330
HO2C
OH
OH
N
H
O
O
O
O
O
(C1)
MS (ESI) m/z calcd for C37H40NO9,
[M+H]+ 642.2703; Found: 642.2703
Counts vs. Mass-to-Charge (m/z)

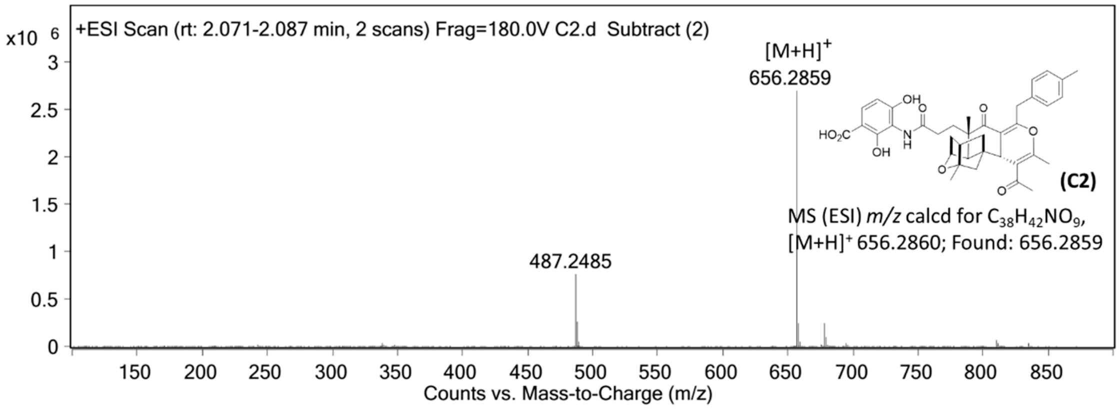
+ESI Scan (rt: 2.071-2.087 min, 2 scans) Frag=180.0V C2.d Subtract (2)
x10 6
[M+H]+
656.2859
487.2485
(C2)
MS (ESI) m/z calcd for C38H42NO9,
[M+H]+ 656.2860; Found: 656.2859
Counts vs. Mass-to-Charge (m/z)

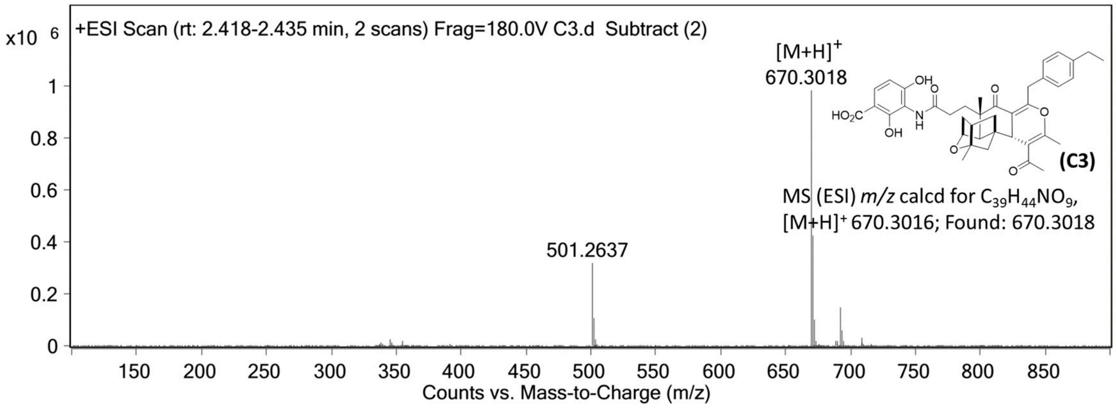
+ESI Scan (rt: 2.418-2.435 min, 2 scans) Frag=180.0V C3.d Subtract (2)
x10 6
[M+H]+
670.3018
501.2637
(C3)
MS (ESI) m/z calcd for C39H44NO9,
[M+H]+ 670.3016; Found: 670.3018
Counts vs. Mass-to-Charge (m/z)

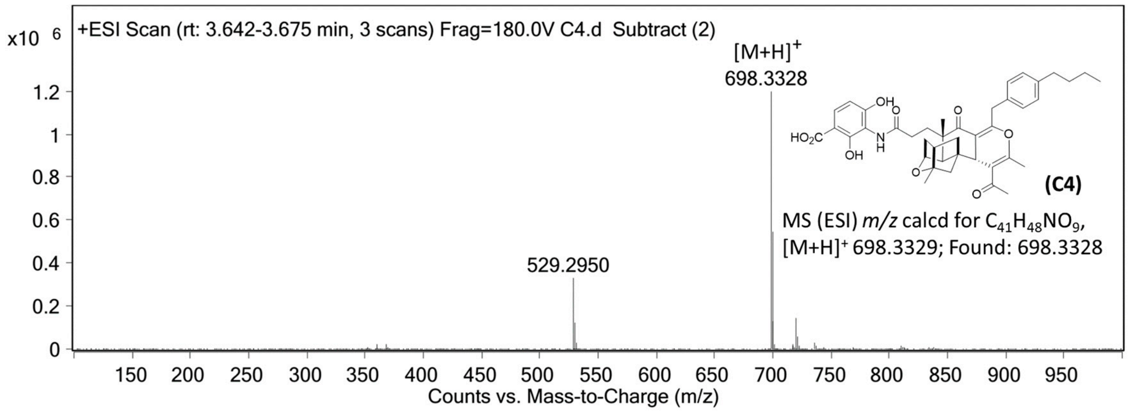
+ESI Scan (rt: 3.642-3.675 min, 3 scans) Frag=180.0V C4.d Subtract (2)
x10 6
[M+H]+
698.3328
529.2950
(C4)
MS (ESI) m/z calcd for C41H48NO9,
[M+H]+ 698.3329; Found: 698.3328
Counts vs. Mass-to-Charge (m/z)

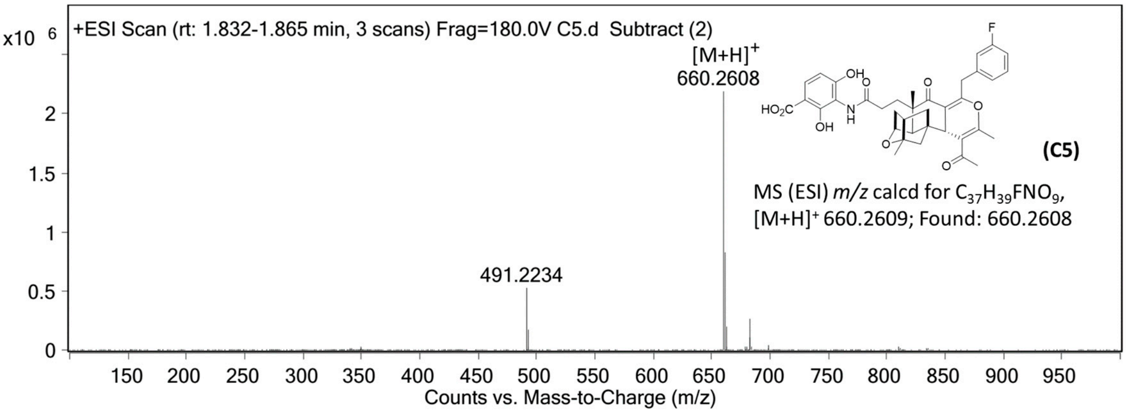

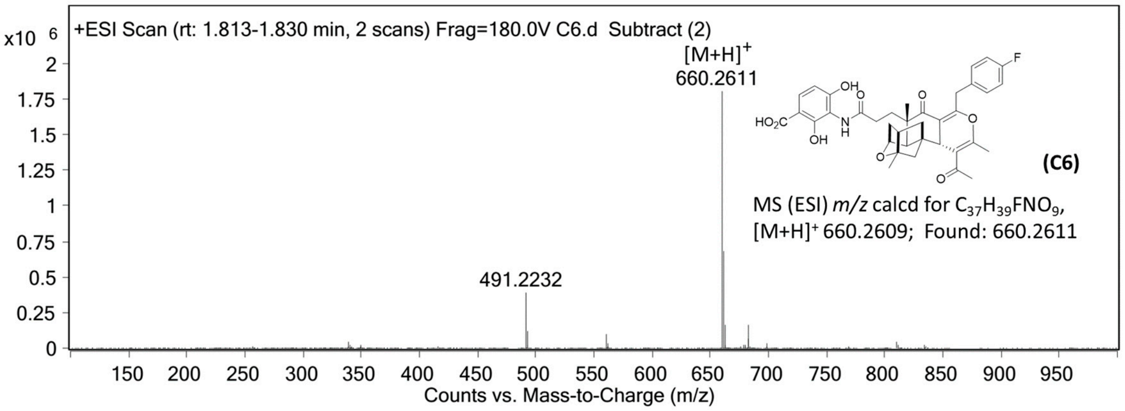

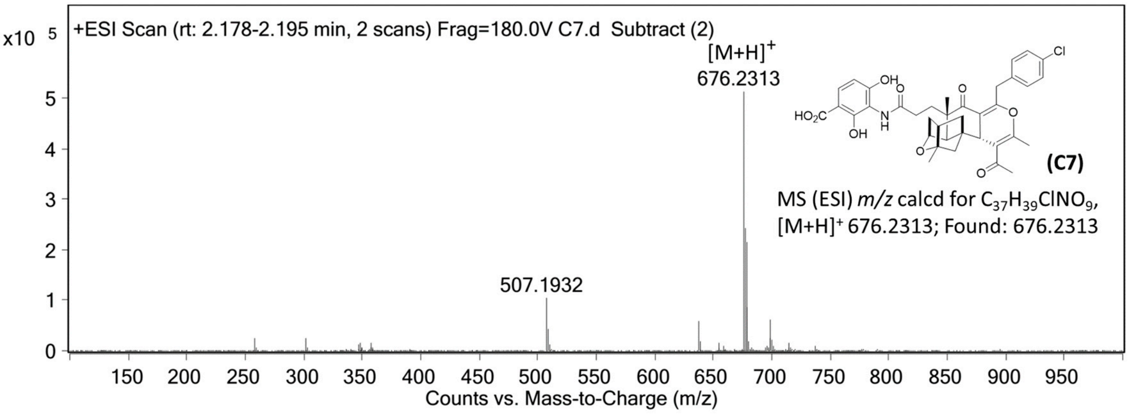

Figure S43. HRMS spectra of **C1 – C7**.

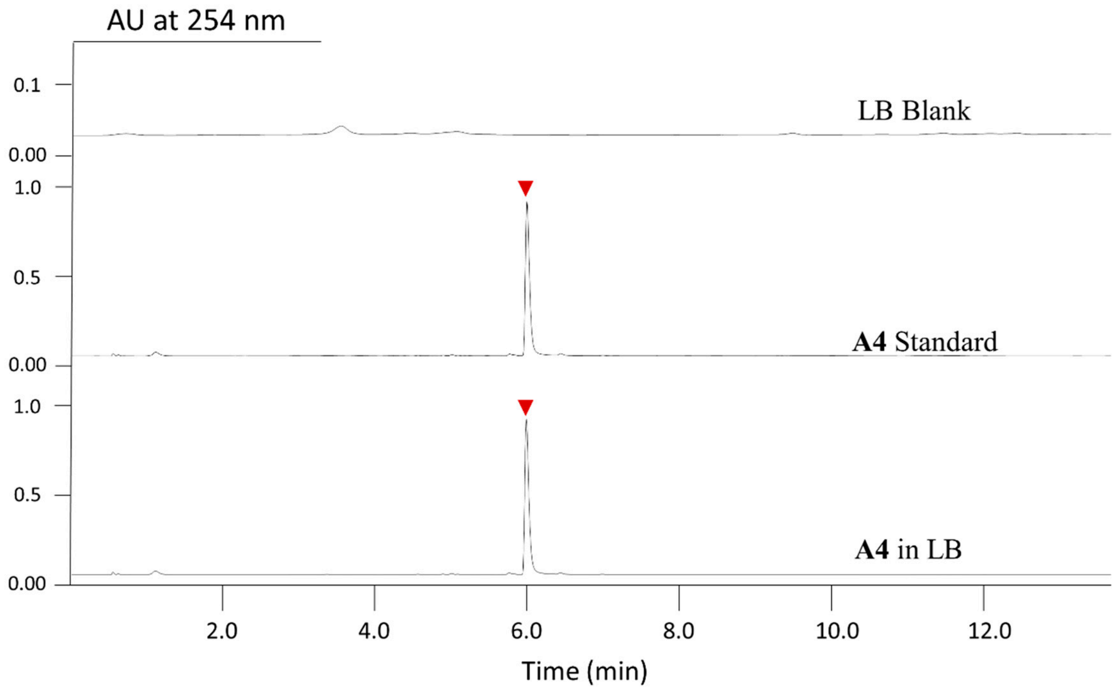

Figure S44. Analysis of the stability of **A4** from LB agar. Compound **A4** (0.1 mg mL^{-1}) were diluted in 5 mL of LB agar (1.5% v/v) to 8 μg mL^{-1}, and the LB agar plates were then incubated at 37 °C for 16 h. The LB agar containing **A4** was extracted by DCM (3 × 10 mL), dried with anhydrous Na_2SO_4 and concentrated in vacuum. The resulting extracts were analysed by Ultra-Performance Liquid Chromatography.

Computational Data

acetylacetone ion
(M06-2X/6-31+G(d)/CPCM (DMSO)

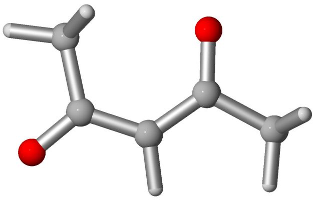

C	1.18855300	0.07580400	-0.00001000
C	0.03039400	-0.73556300	-0.00004900
C	-1.32065300	-0.29156100	0.00005900
C	-1.63377100	1.19530800	0.00002800
C	2.53390200	-0.64548200	0.00002000
O	1.22534500	1.33426000	-0.00002400
O	-2.28905700	-1.09267700	-0.00001300
H	0.16965300	-1.81385700	0.00002000
H	-1.19273200	1.68482300	0.87345000
H	-2.71819200	1.33080000	0.00011300
H	-1.19294500	1.68447200	-0.87370700
H	2.44024200	-1.73438800	-0.00026000
H	3.10640200	-0.33800200	0.88215100
H	3.10671200	-0.33755100	-0.88175100

0 imaginary frequency
Zero-point correction= 0.110798 (Hartree/Particle)
Thermal correction to Energy= 0.118525
Thermal correction to Enthalpy= 0.119469
Thermal correction to Gibbs Free Energy= 0.078685
Sum of electronic and zero-point Energies= -345.077016
Sum of electronic and thermal Energies= -345.069290
Sum of electronic and thermal Enthalpies= -345.068345
Sum of electronic and thermal Free Energies= -345.109129
M06-2X/6-31+G(d) solution-phase Gibbs free energy= -345.18781412

3a
(M06-2X/6-31+G(d)/CPCM (DMSO)

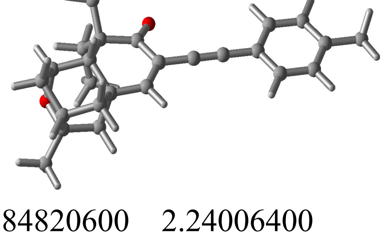

C	2.22721400	1.84820600	2.24006400
C	2.37038100	2.45354400	0.84256800
C	1.77797800	1.69721100	-0.37723700

C	0.28697100	1.46516200	-0.11169200
C	-0.29770200	0.09355300	-0.21826300
O	-0.43092100	2.40655300	0.18656100
C	0.49031100	-1.00135300	-0.29604400
C	2.49917400	0.40434300	-0.85564500
C	1.97370400	-0.95684200	-0.31776600
C	4.04034900	0.43836100	-0.73002500
O	4.43386300	0.06047500	0.58944500
C	4.07154700	-1.33433800	0.69823500
C	2.58175100	-1.34740900	1.05069700
C	4.67451300	-0.68225300	-1.55626500
C	2.63904100	-2.06660300	-1.16637700
C	4.12153800	-1.88452900	-0.78028200
C	4.99083300	-1.99775400	1.69703000
C	1.83025800	2.69192700	-1.56257100
C	-1.72456200	-0.01399200	-0.14912300
C	-2.93349800	-0.10572600	-0.09767500
C	-4.36060000	-0.22719100	-0.03653000
C	-4.96598100	-1.49036000	0.02788200
C	-6.35248700	-1.60137300	0.08669700
C	-7.17076100	-0.46717400	0.08366000
C	-6.55800300	0.79170400	0.01791800
C	-5.17590300	0.91748800	-0.04104300
C	-8.67150600	-0.58316600	0.15692800
H	2.34938500	2.63533500	2.99106700
H	2.98852600	1.09093200	2.43043800
H	1.23763000	1.40153600	2.39727200
H	3.42843600	2.65320400	0.63784300
H	1.87017800	3.42766800	0.84512300
H	0.02342800	-1.98741500	-0.28978600
H	2.28675700	0.35700100	-1.93339900
H	4.45075900	1.43097100	-0.93346600
H	2.34421800	-0.66926400	1.86848200
H	2.23908900	-2.35374600	1.32255900
H	4.38624900	-0.68387900	-2.61110800
H	5.76408400	-0.61019600	-1.48221100
H	2.46564100	-1.95010700	-2.24132600
H	2.25388000	-3.04626700	-0.86066700
H	4.71862700	-2.79428000	-0.87812100
H	4.91107500	-1.50702500	2.67309200
H	6.03025200	-1.93667700	1.35803700
H	4.72270300	-3.05212600	1.81625000
H	1.34025500	3.63218300	-1.29853800
H	1.33687900	2.27891000	-2.44888200
H	2.87323000	2.90439700	-1.82282800
H	-4.34524300	-2.38147400	0.03089900
H	-6.80580900	-2.58833800	0.13675800
H	-7.17620700	1.68651600	0.01254000
H	-4.71634500	1.90023500	-0.09369500
H	-8.99090100	-1.62733800	0.10840300

H	-9.14783100	-0.03984300	-0.66553000
H	-9.04984500	-0.15575000	1.09189700

0 imaginary frequency
Zero-point correction= 0.476078 (Hartree/Particle)
Thermal correction to Energy= 0.499837
Thermal correction to Enthalpy= 0.500781
Thermal correction to Gibbs Free Energy= 0.422741
Sum of electronic and zero-point Energies= -1118.999717
Sum of electronic and thermal Energies= -1118.975958
Sum of electronic and thermal Enthalpies= -1118.975014
Sum of electronic and thermal Free Energies= -1119.053054
M06-2X/6-31+G(d) solution-phase Gibbs free energy= -1119.47579501

3b
(M06-2X/6-31+G(d)/CPCM (DMSO)

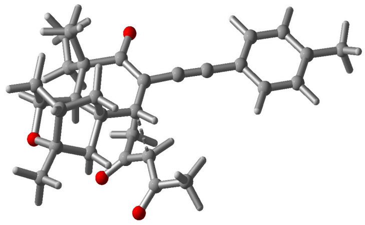

C	1.71675900	4.31183900	1.61345200
H	1.94395500	4.61790500	2.64055000
H	0.66633300	4.52684500	1.40916800
H	2.33112200	4.92624000	0.94736400
C	2.02517000	2.82397000	1.44933000
H	1.50017000	2.25649100	2.22844700
H	3.09574000	2.67624300	1.64457800
C	1.71097900	2.15018500	0.07680600
C	0.18652700	1.95249000	-0.03849800
C	-0.37140300	0.65715300	-0.33594100
O	-0.54041300	2.95511600	0.08221400
C	0.40104000	-0.51658000	-0.47174900
H	-0.05978900	-1.32113700	-1.04415900
C	2.49594400	0.80223300	0.08268100
H	2.49755900	0.47489800	1.13292300
C	1.87902100	-0.39184000	-0.69669500
C	4.00163000	0.91484400	-0.28743800
H	4.50277500	1.69649900	0.29071000
O	4.62496600	-0.33274200	0.04067900
C	4.06755600	-1.27438200	-0.90613500
C	2.69246400	-1.65368200	-0.35772100
H	2.27404400	-2.51720800	-0.89337200
H	2.72646600	-1.88708000	0.70882400
C	4.30104100	0.96223900	-1.79261300
H	3.83993100	1.77484200	-2.35262100
H	5.38733100	1.02132500	-1.91918900
C	2.21336500	-0.30645700	-2.20973400
H	1.75325600	-1.15747800	-2.72702600

H	1.85825400	0.60929400	-2.69159600
C	3.75062700	-0.41109700	-2.18555800
H	4.18192900	-0.82167300	-3.10227200
C	5.04777400	-2.40934800	-1.09031100
H	5.23691400	-2.91044100	-0.13476900
H	5.99869900	-2.03393300	-1.48328000
H	4.64538600	-3.14551500	-1.79331200
C	2.09269900	3.11768000	-1.05861300
H	3.14418100	3.41771300	-0.98867900
H	1.47698800	4.01671200	-0.98353700
H	1.92012200	2.68121000	-2.04641700
C	-1.78758300	0.54131700	-0.40162300
C	0.67547200	-1.08471000	2.41416800
C	0.00606600	-1.70878600	1.28352900
H	-1.03222200	-1.40069400	1.17614500
C	0.21712200	-3.10006500	0.90858600
C	-0.76319900	-3.66743300	-0.11003100
H	-1.66440100	-3.05896400	-0.22523000
H	-1.03925500	-4.68588800	0.17647100
H	-0.25997200	-3.72525400	-1.08431300
C	-0.08062600	0.07491800	3.03453100
H	-0.97882900	-0.30113000	3.53879200
H	-0.42186600	0.76895600	2.25583100
H	0.54780500	0.59904300	3.75811900
O	1.77598300	-1.41499700	2.87155700
O	1.12702300	-3.83154100	1.31202500
C	-2.99746700	0.39233800	-0.42536900
C	-4.42011900	0.23533700	-0.45529800
C	-5.00364600	-1.03248900	-0.63328300
C	-5.26825200	1.34444900	-0.30486800
C	-6.38583400	-1.17609900	-0.65688600
H	-4.36173000	-1.90099600	-0.75315300
C	-6.65129200	1.18613600	-0.33073400
H	-4.83452900	2.33115000	-0.16902600
C	-7.23570700	-0.07267700	-0.50445900
H	-6.81665900	-2.16537200	-0.79657400
H	-7.28888400	2.05970300	-0.21450700
C	-8.73299500	-0.24950600	-0.51294800
H	-9.07218700	-0.75315800	0.39941900
H	-9.24473900	0.71476300	-0.57406500
H	-9.05314800	-0.86247000	-1.36150800

1 imaginary frequency (-392.12)

Zero-point correction= 0.587900 (Hartree/Particle)
Thermal correction to Energy= 0.620199
Thermal correction to Enthalpy= 0.621143
Thermal correction to Gibbs Free Energy= 0.522089
Sum of electronic and zero-point Energies= -1464.066815
Sum of electronic and thermal Energies= -1464.034516
Sum of electronic and thermal Enthalpies= -1464.033572
Sum of electronic and thermal Free Energies= -1464.132626

M06-2X/6-31+G(d) solution-phase Gibbs free energy= -1464.65471501

3c

(M06-2X/6-31+G(d)/CPCM (DMSO)

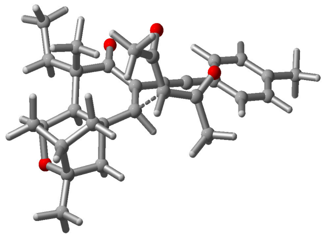

C	-2.21508700	4.38701500	1.15412700
H	-2.52232500	5.36832400	0.77616400
H	-1.16882400	4.44381600	1.45692900
H	-2.82458600	4.17759900	2.03934500
C	-2.43421000	3.33109500	0.06626200
H	-1.96216300	3.66164400	-0.86856900
H	-3.51262100	3.29712900	-0.12362100
C	-1.96111700	1.89035900	0.37155500
C	-0.41897200	1.81169600	0.32137200
C	0.18832600	0.53317700	0.01287600
O	0.26196300	2.83419100	0.49393100
C	-0.54808500	-0.65569600	-0.18336300
H	-0.03098500	-1.41294200	-0.76762000
C	-2.44440200	0.93512600	-0.75881100
H	-1.88665300	1.27064700	-1.64674900
C	-2.02675000	-0.56187700	-0.55846300
C	-3.92095800	1.00737300	-1.22775300
H	-4.22835500	2.02803200	-1.45738500
O	-4.01654400	0.26930500	-2.45427600
C	-3.79600800	-1.10132200	-2.06923200
C	-2.27737100	-1.26397500	-1.91585700
H	-1.99016700	-2.32181000	-1.84441500
H	-1.74159100	-0.80854100	-2.75578100
C	-4.89998600	0.22479500	-0.35612600
H	-4.90189600	0.50150900	0.70071700
H	-5.91103900	0.34800700	-0.75732400
C	-3.08240100	-1.34318500	0.26519800
H	-2.78637100	-2.39535600	0.32217600
H	-3.23570500	-0.98057200	1.28225800
C	-4.34336400	-1.18503900	-0.59475200
H	-5.07615200	-1.98300200	-0.45075300
C	-4.45571900	-2.00615900	-3.08388700
H	-4.03170400	-1.83899400	-4.07996000
H	-5.53268300	-1.81175500	-3.12446600
H	-4.29977300	-3.05532100	-2.81381800
C	-2.39200300	1.49651800	1.79184600
H	-3.47574900	1.59178700	1.92615900
H	-1.90351100	2.14993100	2.52190700

H	-2.09182700	0.47836300	2.02765100
C	1.59974300	0.51138700	-0.17007300
C	-0.59884100	-1.31291300	2.72036300
C	-0.28467100	-2.00741200	1.48541100
H	-1.01023500	-2.75475100	1.17677100
C	1.08793200	-2.44372200	1.23135400
C	1.29078900	-3.37436700	0.04335900
H	0.35458700	-3.75906100	-0.36946900
H	1.93007800	-4.20970800	0.34224000
H	1.81699900	-2.82122100	-0.74491000
C	-1.84181000	-1.73872500	3.49181100
H	-1.50488600	-2.28294500	4.38237000
H	-2.50034600	-2.39790500	2.92240500
H	-2.40030900	-0.86209800	3.83609000
O	0.10220800	-0.40929800	3.18894400
O	2.07475500	-2.12404100	1.89662100
C	2.80327100	0.49679200	-0.36345900
C	4.22024800	0.43083100	-0.54702700
C	4.85911000	-0.80608600	-0.74629600
C	5.01352100	1.59183200	-0.52341900
C	6.23845300	-0.87256200	-0.91490500
H	4.26363100	-1.71474300	-0.76150800
C	6.39202400	1.51079200	-0.69338400
H	4.53954100	2.55685100	-0.36706100
C	7.03070900	0.28145900	-0.89494800
H	6.71016100	-1.84164500	-1.06274700
H	6.98539800	2.42229400	-0.66799700
C	8.52120000	0.20552900	-1.10965300
H	9.03708700	1.02163400	-0.59563400
H	8.92583300	-0.74276200	-0.74423600
H	8.76666400	0.27988700	-2.17568300

1 imaginary frequency (-383.91)

Zero-point correction= 0.588492 (Hartree/Particle)
Thermal correction to Energy= 0.620616
Thermal correction to Enthalpy= 0.621560
Thermal correction to Gibbs Free Energy= 0.525616
Sum of electronic and zero-point Energies= -1464.053170
Sum of electronic and thermal Energies= -1464.021045
Sum of electronic and thermal Enthalpies= -1464.020101
Sum of electronic and thermal Free Energies= -1464.116046

M06-2X/6-31+G(d) solution-phase Gibbs free energy= -1464.64166114

3d

(M06-2X/6-31+G(d)/CPCM (DMSO)

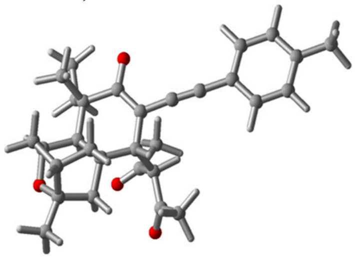

C	1.71463500	4.23150200	1.62808500
H	1.93844200	4.51860900	2.66185500
H	0.65950800	4.42380200	1.42681600
H	2.31259500	4.87578700	0.97478500
C	2.05532400	2.75621900	1.42187600
H	1.56018600	2.15559400	2.19886200
H	3.13472300	2.63040200	1.59162700
C	1.72430100	2.10110600	0.04336700
C	0.19346200	1.91552900	-0.06588400
C	-0.37098700	0.63955400	-0.20727500
O	-0.50425400	2.97601700	-0.03910000
C	0.38781700	-0.67002600	-0.18492100
H	-0.06021700	-1.32451500	-0.94736000
C	2.53082900	0.75955000	0.03930100
H	2.66829600	0.49902100	1.09387700
C	1.85619400	-0.49451900	-0.59587300
C	3.98593600	0.88588200	-0.49437600
H	4.51885900	1.70596200	-0.00360400
O	4.67809400	-0.32903600	-0.17277800
C	4.04826800	-1.32649800	-1.00614900
C	2.75332700	-1.71238400	-0.29093600
H	2.32565600	-2.61856700	-0.74201200
H	2.92276700	-1.88848200	0.77494900
C	4.12949600	0.86328100	-2.02073600
H	3.59564500	1.63893800	-2.56659400
H	5.19530900	0.93051000	-2.26554400
C	2.03212900	-0.45730600	-2.13043900
H	1.53839800	-1.33209200	-2.57426700
H	1.60869200	0.43854600	-2.59743000
C	3.56463000	-0.53462500	-2.28037100
H	3.89904900	-0.98572700	-3.21854200
C	5.03483700	-2.44520100	-1.24895800
H	5.34884400	-2.89305000	-0.29963900
H	5.92252900	-2.06509800	-1.76615900
H	4.57874300	-3.22606400	-1.86587300
C	2.10461200	3.08232300	-1.08043900
H	3.16791800	3.34751200	-1.05197600
H	1.51919000	3.99554600	-0.96163100
H	1.87319100	2.67397700	-2.06935800
C	-1.77521400	0.52506200	-0.28025300
C	0.62744000	-0.73243700	2.40524900

C	0.08536000	-1.43172700	1.16730500
H	-1.01332800	-1.37850000	1.22600700
C	0.35732900	-2.93135300	1.14183700
C	-0.29439900	-3.70533900	0.01292500
H	-1.28300900	-3.30302800	-0.22922900
H	-0.37379900	-4.75577000	0.29829200
H	0.31716000	-3.63572500	-0.89329900
C	-0.36160600	0.10711100	3.17330700
H	-1.15728400	-0.53762700	3.56592000
H	-0.83288000	0.82397600	2.48967300
H	0.13284700	0.62867900	3.99484500
O	1.78817900	-0.83387500	2.75679500
O	0.99051100	-3.50994600	2.00479100
C	-2.98709600	0.35077400	-0.33236500
C	-4.40202500	0.18056700	-0.42358000
C	-4.97802300	-1.09961200	-0.53335200
C	-5.26737000	1.29133700	-0.40545300
C	-6.35778600	-1.25365600	-0.62479500
H	-4.33099500	-1.97282200	-0.54361400
C	-6.64485200	1.12230800	-0.49831100
H	-4.84625200	2.28883800	-0.31420800
C	-7.21858600	-0.14969000	-0.61387600
H	-6.77608400	-2.25484300	-0.70720600
H	-7.28938500	1.99878300	-0.48014600
C	-8.71029800	-0.32227600	-0.75126000
H	-9.01217500	-0.30016400	-1.80520500
H	-9.03915400	-1.27940700	-0.33582100
H	-9.25033900	0.47898100	-0.23835800

0 imaginary frequency
Zero-point correction= 0.590273 (Hartree/Particle)
Thermal correction to Energy= 0.622467
Thermal correction to Enthalpy= 0.623411
Thermal correction to Gibbs Free Energy= 0.526898
Sum of electronic and zero-point Energies= -1464.086325
Sum of electronic and thermal Energies= -1464.054131
Sum of electronic and thermal Enthalpies= -1464.053187
Sum of electronic and thermal Free Energies= -1464.149700
M06-2X/6-31+G(d) solution-phase Gibbs free energy= -1464.67659820

3d′
(M06-2X/6-31+G(d)/CPCM (DMSO)

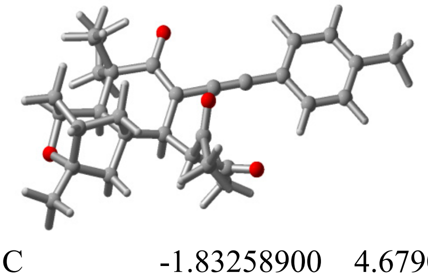

C	-1.83258900	4.67908200	-0.83367100
H	-1.94040300	5.27236300	-1.74883600

H	-0.80440500	4.76227000	-0.47616800
H	-2.49540300	5.11853100	-0.08078700
C	-2.19790200	3.22372300	-1.12288200
H	-1.61452100	2.86958700	-1.98482000
H	-3.25048900	3.19263700	-1.43984200
C	-2.02244000	2.17362200	0.02004200
C	-0.51010300	1.93274200	0.22149000
C	0.06945900	0.69438200	-0.09767600
O	0.15666200	2.92020700	0.66197800
C	-0.71379900	-0.50336600	-0.60950700
H	-0.78123200	-0.44688800	-1.71114100
C	-2.79610500	0.91055200	-0.48308900
H	-2.76861600	0.97106400	-1.58245700
C	-2.19087700	-0.49351300	-0.16700300
C	-4.31166400	0.92188600	-0.13930000
H	-4.78116100	1.86716800	-0.42743700
O	-4.94566300	-0.12042400	-0.89180400
C	-4.41847600	-1.33664400	-0.31893200
C	-3.03768700	-1.53141600	-0.93944100
H	-2.68122400	-2.55000900	-0.74011300
H	-3.05836200	-1.37398300	-2.02397100
C	-4.65182700	0.45191200	1.28153100
H	-4.20667600	1.01394600	2.10031800
H	-5.74135000	0.46960700	1.39410600
C	-2.56723100	-0.91327300	1.26807800
H	-2.16611000	-1.90671200	1.49667200
H	-2.21027100	-0.22578400	2.04187100
C	-4.10879100	-0.97358800	1.18350400
H	-4.55866400	-1.67192100	1.89430900
C	-5.41518500	-2.44972400	-0.54519900
H	-5.59900300	-2.58862500	-1.61628500
H	-6.36639600	-2.21582800	-0.05506600
H	-5.03325100	-3.38992700	-0.13498500
C	-2.55055600	2.77241500	1.33411800
H	-3.60529500	3.06171400	1.26110700
H	-1.96593800	3.66191200	1.57343000
H	-2.43577100	2.07566100	2.17017100
C	1.47358000	0.61449100	-0.01891500
C	0.19057200	-2.31202100	1.08826300
C	-0.02406700	-1.87104300	-0.36140000
H	-0.66805300	-2.63213600	-0.83004100
C	1.29029500	-2.08812700	-1.12125300
C	1.39517000	-1.54555700	-2.52466200
H	0.53885300	-1.87626700	-3.12332800
H	2.32349800	-1.89977600	-2.97615100
H	1.38763200	-0.45169700	-2.50846300
C	-0.05345400	-3.77605400	1.36651800
H	0.56419700	-4.38068100	0.69388100
H	-1.10099500	-4.02640400	1.15912400
H	0.18188200	-4.01094900	2.40588100

O	0.55073000	-1.54891700	1.96118800
O	2.17398700	-2.77707500	-0.64088000
C	2.69693800	0.55558400	0.02529500
C	4.12175500	0.48313300	0.06451100
C	4.79852000	-0.70037900	-0.29205000
C	4.89820400	1.59364800	0.44988800
C	6.18809300	-0.76088200	-0.25977800
H	4.21765500	-1.56740200	-0.59452900
C	6.28745000	1.51670400	0.47658200
H	4.39938500	2.51974500	0.72334100
C	6.96137700	0.34079400	0.12656400
H	6.68568100	-1.68658700	-0.54343700
H	6.86188300	2.39192800	0.77382700
C	8.46581100	0.25376700	0.18813600
H	8.79941000	-0.12455800	1.16182600
H	8.85517200	-0.42501600	-0.57669500
H	8.92577700	1.23558300	0.04226300

0 imaginary frequency
Zero-point correction= 0.589861 (Hartree/Particle)
Thermal correction to Energy= 0.621997
Thermal correction to Enthalpy= 0.622941
Thermal correction to Gibbs Free Energy= 0.526360
Sum of electronic and zero-point Energies= -1464.084603
Sum of electronic and thermal Energies= -1464.052466
Sum of electronic and thermal Enthalpies= -1464.051522
Sum of electronic and thermal Free Energies= -1464.148103
M06-2X/6-31+G(d) solution-phase Gibbs free energy= -1464.67446323

3e
(M06-2X/6-31+G(d)/CPCM (DMSO)

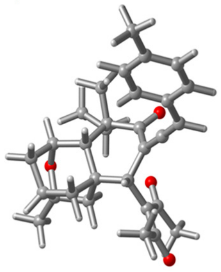

C	-2.63230300	-3.06900400	2.45444200
C	-2.06499800	-1.65639700	2.28931300
C	-0.78660500	-1.49311900	1.42379400
C	-0.04524900	-0.23485500	1.90566200
O	0.27428100	-0.10413800	3.07535400
C	-0.46701900	1.08734200	-0.32754100
C	-0.95819000	-1.45625100	-0.12446100
C	-1.39894900	-0.09314900	-0.75605100
C	-1.85772200	-2.59357800	-0.67131600
O	-3.23688100	-2.25845700	-0.50868500
C	-3.43946300	-1.13261000	-1.39067200

C	-2.92535000	0.09188000	-0.63065200
C	-1.76836100	-2.69720400	-2.19514000
C	-1.34139500	-0.26223200	-2.29362100
C	-2.39524200	-1.34566900	-2.55532100
C	-4.89468700	-1.09506100	-1.79685100
C	0.21239700	-2.62683900	1.75816100
C	-1.94627000	2.47504500	1.11838700
C	-1.16897200	2.41137300	-0.07541500
C	-1.00251900	3.51848700	-0.95580100
C	-0.02588900	3.40040200	-2.13122800
C	-2.70366700	3.72622400	1.52350900
O	-2.02031000	1.48369900	1.88959100
O	-1.59380900	4.62299500	-0.84375600
H	-3.46817300	-3.03495300	3.16229400
H	-1.89195900	-3.76407800	2.86352500
H	-3.01722900	-3.47987700	1.51916200
H	-1.79617600	-1.28903500	3.28682400
H	-2.84169200	-0.98870400	1.91465800
H	0.21975000	1.23108500	-1.16530200
H	0.05047100	-1.64965700	-0.52662300
H	-1.67872400	-3.54113000	-0.15748300
H	-3.31477600	0.12087100	0.38575500
H	-3.21430100	1.02132300	-1.13702100
H	-2.37691500	-3.54306700	-2.53137000
H	-0.75136200	-2.81347900	-2.58074000
H	-0.34364000	-0.54131500	-2.65507300
H	-1.61551800	0.69254700	-2.76105800
H	-2.84680400	-1.30959900	-3.55055200
H	-5.53650400	-0.99629100	-0.91422800
H	-5.16982400	-2.01278700	-2.32794200
H	-5.08103800	-0.24140200	-2.45637700
H	0.31936200	-2.73565300	2.84149500
H	1.20145300	-2.41047600	1.33590800
H	-0.12508600	-3.58270400	1.34646300
H	-0.31438400	2.60960100	-2.83160900
H	0.99247700	3.17888400	-1.79180000
H	-0.01999100	4.35327500	-2.66408000
H	-3.46906200	3.97419700	0.78186000
H	-2.04128800	4.59466200	1.57388800
H	-3.17063700	3.54623200	2.49535600
C	0.44164600	0.69758700	0.84184800
C	1.68988300	1.09228400	0.91336200
C	2.96035900	1.42826100	0.95240000
H	3.24371300	2.35561100	1.45198600
C	4.05703900	0.62832000	0.36592000
C	5.38127600	1.04900200	0.51877100
C	3.81425300	-0.55634600	-0.34714500
C	6.43378100	0.30691500	-0.01912300
H	5.59220500	1.96513800	1.06575500
C	4.86551700	-1.29022700	-0.88012800

H	2.78981700	-0.89820700	-0.48294300
C	6.19651600	-0.87397700	-0.72499100
H	7.45536800	0.65544400	0.11445700
H	4.65423000	-2.20532800	-1.42975800
C	7.32522000	-1.68715700	-1.30631000
H	7.35533100	-2.68894900	-0.86397200
H	7.20287600	-1.81323300	-2.38741300
H	8.29154500	-1.20843000	-1.12688200

0 imaginary frequency

Zero-point correction= 0.590218 (Hartree/Particle)
Thermal correction to Energy= 0.621971
Thermal correction to Enthalpy= 0.622915
Thermal correction to Gibbs Free Energy= 0.526890
Sum of electronic and zero-point Energies= -1464.099024
Sum of electronic and thermal Energies= -1464.067271
Sum of electronic and thermal Enthalpies= -1464.066327
Sum of electronic and thermal Free Energies= -1464.162352
M06-2X/6-31+G(d) solution-phase Gibbs free energy= -1464.68924204

3f
(M06-2X/6-31+G(d)/CPCM (DMSO)

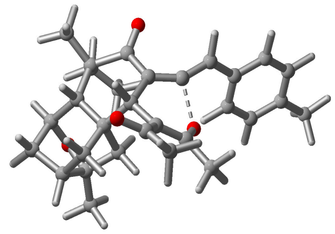

C	-0.05978800	-3.18298800	0.18418100
C	-1.16172100	-3.21595200	-0.87713000
C	-1.65007000	-1.87504700	-1.51239100
C	-0.35619900	-1.16709500	-1.96728600
C	-0.02271700	0.14288200	-1.41043000
O	0.38899800	-1.77632200	-2.73914900
C	-1.14141500	1.04445200	-0.92316000
C	-2.61384500	-0.98868700	-0.64988500
C	-2.00849400	0.25440800	0.07694900
C	-3.47184600	-1.80606400	0.34449800
O	-2.71179500	-2.09287900	1.51983500
C	-2.49877900	-0.80180500	2.13506300
C	-1.32574200	-0.15377000	1.38947600
C	-4.60362500	-0.96451500	0.93710300
C	-3.18834000	1.05106600	0.67160200
C	-3.75292300	0.05697600	1.70193300
C	-2.30653800	-1.00703200	3.62006900
C	-2.43811400	-2.26841700	-2.78017900
C	1.27912100	0.40539900	-1.20534900
O	1.27511200	1.41752800	0.43551800
C	0.50932600	2.44734700	0.33288600
C	-0.67520700	2.39307100	-0.40842800

C	2.55816000	0.24850900	-1.56859200
C	3.69562800	-0.05523300	-0.69782700
C	-1.51513200	3.51930000	-0.75220900
C	1.03337600	3.64590900	1.10388000
C	-1.15004700	4.96302500	-0.42806000
O	-2.58698600	3.35782800	-1.36844600
C	3.53750000	-0.49533000	0.63066000
C	4.64037200	-0.78815800	1.42345700
C	5.94941500	-0.67089200	0.93257600
C	6.10794500	-0.24969100	-0.38960700
C	5.00537500	0.05170500	-1.18966100
H	0.34350800	-4.19333900	0.31631000
H	-0.43874200	-2.84593400	1.14938000
H	0.77358700	-2.53523600	-0.11460000
H	-2.02824600	-3.75779800	-0.48288700
H	-0.78810500	-3.80811800	-1.71907200
H	-1.80092700	1.24982600	-1.77780900
H	-3.32458500	-0.55607400	-1.37024900
H	-3.81087000	-2.75420400	-0.08205600
H	-0.47950500	-0.83370400	1.27602000
H	-0.97539600	0.74757900	1.90966800
H	-5.20523700	-1.59314600	1.60193100
H	-5.25869000	-0.50041300	0.19403600
H	-3.92929300	1.34945400	-0.07719100
H	-2.80574300	1.96200500	1.14655700
H	-4.26515700	0.52391500	2.54790400
H	-1.44744000	-1.66137200	3.80726800
H	-3.19720300	-1.46395300	4.06515300
H	-2.12315000	-0.04707700	4.11332100
H	-2.74788900	-1.38023200	-3.34264600
H	-3.34233000	-2.82427700	-2.50187200
H	-1.83121600	-2.89875900	-3.43490600
H	2.76128100	0.34361900	-2.63470800
H	1.70652800	3.26612200	1.87583600
H	0.24971700	4.23694600	1.57721300
H	1.61845500	4.30288700	0.44964000
H	-1.38282000	5.19635000	0.61665900
H	-1.76684400	5.60447000	-1.06061700
H	-0.09769800	5.19408100	-0.60044600
H	2.53481700	-0.59853600	1.03680100
H	4.48592100	-1.12463800	2.44759100
H	7.10954400	-0.15215100	-0.80383600
H	5.16159200	0.38070900	-2.21523200
C	7.13335100	-1.00155300	1.80705600
H	8.07527900	-0.81481500	1.28354400
H	7.13018700	-0.40009900	2.72268600
H	7.11988800	-2.05424900	2.11138500

1 imaginary frequency (-420.07)

Zero-point correction= 0.589513 (Hartree/Particle)

Thermal correction to Energy= 0.620354

Thermal correction to Enthalpy= 0.621299
Thermal correction to Gibbs Free Energy= 0.528418
Sum of electronic and zero-point Energies= -1464.072661
Sum of electronic and thermal Energies= -1464.041820
Sum of electronic and thermal Enthalpies= -1464.040876
Sum of electronic and thermal Free Energies= -1464.133756
M06-2X/6-31+G(d) solution-phase Gibbs free energy= -1464.66217420

3g
(M06-2X/6-31+G(d)/CPCM (DMSO)

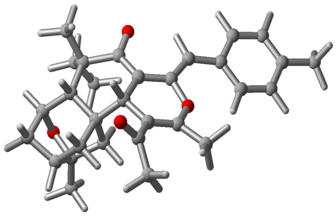

C	-1.09972700	-3.25819600	1.18070900
C	-1.82335100	-3.31122000	-0.16771600
C	-1.78571100	-2.06125500	-1.10302200
C	-0.28520800	-1.66340800	-1.19563800
C	0.11669600	-0.33880600	-0.93408200
O	0.50652200	-2.61970400	-1.43972700
C	-0.93467600	0.75694800	-0.97789700
C	-2.77590500	-0.89700300	-0.73523900
C	-2.14164500	0.37700900	-0.09948300
C	-3.99771800	-1.34356700	0.10154900
O	-3.62280900	-1.48265100	1.47442300
C	-3.30067400	-0.14079200	1.89717500
C	-1.87443200	0.14723000	1.39344900
C	-5.04624000	-0.23613400	0.20848500
C	-3.24594100	1.44927900	0.03139900
C	-4.22460800	0.78091700	1.00756100
C	-3.50208800	-0.04820800	3.39206400
C	-2.21765100	-2.56783600	-2.49539300
C	1.46786600	-0.03633500	-0.57191300
O	1.71985100	1.22591100	-0.02626800
C	0.88895500	2.28001500	-0.22190600
C	-0.36914500	2.12745800	-0.67928700
C	2.58277000	-0.83818300	-0.62011200
C	3.94115400	-0.58560400	-0.14620500
C	-1.18577300	3.29948200	-1.09170700
C	1.63962300	3.54264600	0.09510200
C	-1.27003500	4.54972000	-0.23350800
O	-1.85641300	3.24889900	-2.11664700
C	4.40037300	0.51595000	0.61121100
C	5.73430900	0.62061600	1.00811400
C	6.68657000	-0.34809600	0.68423500
C	6.24159100	-1.45133000	-0.05709400
C	4.91672000	-1.56475400	-0.45642200
H	-0.99604200	-4.27555900	1.57688500

H	-1.64956500	-2.66988000	1.91615700
H	-0.09115100	-2.84382100	1.07373000
H	-2.86513400	-3.60982200	-0.00574100
H	-1.36405800	-4.11668700	-0.74854900
H	-1.34126100	0.82960600	-2.00022300
H	-3.18811700	-0.54535100	-1.69360000
H	-4.40700200	-2.29785900	-0.23970100
H	-1.19602300	-0.67908600	1.61257600
H	-1.46913000	1.06468600	1.84450600
H	-5.90870200	-0.61208300	0.76884700
H	-5.38945700	0.15678800	-0.75293200
H	-3.71292900	1.72328700	-0.92103000
H	-2.82357400	2.35938600	0.47570900
H	-4.82450500	1.48246300	1.59402600
H	-2.85259200	-0.76288700	3.91019200
H	-4.54284600	-0.26812900	3.65366500
H	-3.25803800	0.95888000	3.74527200
H	-2.17017300	-1.76265900	-3.23862800
H	-3.25142600	-2.93849000	-2.46610700
H	-1.56632900	-3.38194400	-2.82559000
H	2.42042500	-1.80178000	-1.08164000
H	2.65949600	3.44508200	-0.28685300
H	1.70280600	3.68253700	1.18057800
H	1.19180900	4.43001300	-0.34583600
H	-0.83746600	4.42102800	0.75985000
H	-2.32826100	4.81061400	-0.13835000
H	-0.77556700	5.38527400	-0.74054500
H	3.71054000	1.29682300	0.90367600
H	6.03770000	1.48726600	1.59378000
H	6.94780400	-2.23625400	-0.32450100
H	4.61048500	-2.43835100	-1.02892200
C	8.13217800	-0.21307200	1.09296500
H	8.76207300	0.06747800	0.24024900
H	8.25512900	0.55507600	1.86220100
H	8.52422000	-1.15610800	1.48825200

0 imaginary frequency

Zero-point correction= 0.593077 (Hartree/Particle)
Thermal correction to Energy= 0.623610
Thermal correction to Enthalpy= 0.624554
Thermal correction to Gibbs Free Energy= 0.533405
Sum of electronic and zero-point Energies= -1464.105542
Sum of electronic and thermal Energies= -1464.075010
Sum of electronic and thermal Enthalpies= -1464.074065
Sum of electronic and thermal Free Energies= -1464.165214

M06-2X/6-31+G(d) solution-phase Gibbs free energy= -1464.69861925
